# Supplementary material for: Habitat use of loggerhead (Caretta caretta) and green (Chelonia mydas) turtles at the northern limit of their distribution range of the Northwest Pacific Ocean
Source: PLoS One. 2024 Apr 4;19(4):e0290202. doi: 10.1371/journal.pone.0290202 (PMC10994308; doi:10.1371/journal.pone.0290202)
Supplement: S1 Table — (DOCX) [file pone.0290202.s001.docx]

**S1 Table. Rescue and release data of tracked sea turtles.**

| ID | Rescue date | Rescue location (°) | | Release date | Release location (°) | | DRR (km) |
| --- | --- | --- | --- | --- | --- | --- | --- |
|  |  | Latitude | Longitude |  | Latitude | Longitude |  |
| Loggerhead turtle | | | | | | | |
| KOR0001 | 2015-06-25 | 34.6542 | 127.8060 | 2016-06-17 | 34.6306 | 127.7935 | 2.8 |
| KOR0008 | 2017-08-07 | 34.4950 | 127.8070 | 2017-09-28 | 33.2449 | 126.4128 | 189.4 |
| KOR0091 | 2017-11-21 | 37.2894 | 129.3170 | 2018-08-29 | 33.2449 | 126.4128 | 520.6 |
| KOR0092 | 2017-11-25 | 37.2894 | 129.3170 | 2018-08-29 | 33.2449 | 126.4128 | 520.6 |
| KOR0149 | 2019-10-24 | 36.8293 | 129.4517 | 2021-08-26 | 33.2449 | 126.4128 | 484.7 |
| KOR0151 | 2019-11-04 | 37.5294 | 129.1212 | 2021-08-26 | 33.2449 | 126.4128 | 535.3 |
| KOR0155 | 2021-06-17 | 36.5140 | 129.4578 | 2022-08-25 | 33.2449 | 126.4128 | 457.1 |
| Green turtle | | | | | | | |
| KOR-1 | 2014-11-11 | 34.9622 | 128.7301 | 2015-10-29 | 35.1587 | 129.1608 | 44.9 |
| KOR-2 | 2014-10-19 | 35.1728 | 129.2030 | 2015-10-29 | 35.1587 | 129.1608 | 4.1 |
| KOR0003 | 2010-08-15 | 33.3794 | 126.8832 | 2016-08-08 | 35.0755 | 129.0168 | 272.1 |
| KOR0004 | 2013-07-01 | 33.4460 | 126.2835 | 2016-09-02 | 33.3820 | 126.8812 | 56.3 |
| KOR0009 | 2017-09-04 | 34.6423 | 129.8047 | 2017-09-28 | 33.2449 | 126.4128 | 349.7 |
| KOR0104 | 2018-10-02 | 34.6805 | 127.7833 | 2018-10-04 | 34.5986 | 127.8010 | 9.2 |
| KOR0129 | 2019-07-27 | 34.4760 | 127.8161 | 2019-08-28 | 33.2449 | 126.4128 | 188.4 |
| KOR0148 | 2019-08-22 | 34.6038 | 127.7943 | 2021-08-26 | 33.2449 | 126.4128 | 484.7 |

DRR: Distance from the rescue location to the release location.
