# Supplementary material for: Habitat use of loggerhead (Caretta caretta) and green (Chelonia mydas) turtles at the northern limit of their distribution range of the Northwest Pacific Ocean
Source: PLoS One. 2024 Apr 4;19(4):e0290202. doi: 10.1371/journal.pone.0290202 (PMC10994308; doi:10.1371/journal.pone.0290202)
Supplement: S2 Table — (DOCX) [file pone.0290202.s002.docx]

**S2 Table. Comparison of the daily distance traveled (km/d) by sea turtles between 50 PVCs and outer areas.** For statistical comparison, independent t-test, One-way ANOVA with Bonferroni post hoc test, or Kruskal-Wallis test with Dunn post hoc test were conducted. Asterisk indicates significant differences (p-value < 0.05).

| ID | Statistical test | p-value | 50 PVC outer area | | 50 PVC 1 | | | 50 PVC 2 | | | 50 PVC 3 | | | 50 PVC 4 | | |
| --- | --- | --- | --- | --- | --- | --- | --- | --- | --- | --- | --- | --- | --- | --- | --- | --- |
|  |  |  | n | Mean | n | Mean | Post hoc  p-value | n | Mean | Post hoc  p-value | n | Mean | Post hoc  P-value | n | Mean | Post hoc  p-value |
| Loggerhead turtle | | | | | | | | | | | | | | | | |
| KOR0001 | ANOVA  F = 2.054 | 0.160 | 6 | 7.6  ± 4.2 | 2 | 6.2  ± 1.2 | 1.000 | 3 | 6.9  ± 3.8 | 1.000 | 5 | 2.7  ± 2.4 | 0.209 |  |  |  |
| KOR0008 | t-test  t = 6.526 | 0.000* | 38 | 55.1  ± 34.3 | 19 | 3.5  ± 2.7 |  |  |  |  |  |  |  |  |  |  |
| KOR0091 | Kruskal-Wallis | 0.032* | 20 | 29.9  ± 15.6 | 2 | 17.9  ± 2.6 | 1.000 | 14 | 21.4  ± 10.6 | 0.415 | 6 | 12.5  ± 8.5 | 0.023* |  |  |  |
| KOR0092 | ANOVA  F = 14.230 | 0.000* | 125 | 24.2  ± 18.6 | 61 | 18.2  ± 15.3 | 0.031* | 165 | 14.7  ± 11.6 | 0.000* |  |  |  |  |  |  |
| KOR0149 | t-test  t = 1.714 | 0.090* | 40 | 20.8  ± 14.3 | 55 | 16.7  ± 8.5 |  |  |  |  |  |  |  |  |  |  |
| KOR0151 | t-test  t = 1.505 | 0.030* | 235 | 23.1  ± 15.5 | 159 | 20.8  ± 12.6 |  |  |  |  |  |  |  |  |  |  |
| KOR0155 | Kruskal-Wallis | 0.057 | 11 | 24.2  ± 11.6 | 15 | 15.0  ± 7.1 |  | 3 | 17.8  ± 2.9 |  | 4 | 13.2  ± 3.7 |  |  |  |  |
| Green turtle | | | | | | | | | | | | | | | | |
| KOR-1 | Kruskal-Wallis | 0.063 | 4 | 57.3  ± 20.1 | 5 | 28.7  ± 16.5 |  | 4 | 13.2  ± 12.8 |  | 2 | 33.0  ± 24.4 |  |  |  |  |
| KOR-2 | t-test  t = 12.165 | 0.000* | 23 | 27.8  ± 18.4 | 179 | 3.5  ± 7.0 |  |  |  |  |  |  |  |  |  |  |
| KOR0003 | t-test  t = 3.657 | 0.000* | 30 | 33.2  ± 25.3 | 12 | 5.6  ± 9.4 |  |  |  |  |  |  |  |  |  |  |
| KOR0004 | Kruskal-Wallis | 0.108 | 15 | 35.0  ± 20.1 | 15 | 21.5  ± 13.3 |  | 9 | 20.0  ± 13.9 |  |  |  |  |  |  |  |
| KOR0009 | ANOVA  F = 21.664 | 0.000* | 170 | 25.1  ± 19.8 | 70 | 7.9  ± 6.7 | 0.000* | 60 | 14.6  ± 11.2 | 0.000* | 31 | 15.4  ± 14.1 | 0.012* |  |  |  |
| KOR0104 | t-test  t = 7.320 | 0.000* | 144 | 32.6  ± 21.3 | 33 | 5.3  ± 5.3 |  |  |  |  |  |  |  |  |  |  |
| KOR0129 | Kruskal-Wallis | 0.022* | 23 | 55.4  ± 30.0 | 3 | 19.1  ± 13.5 | 0.256 | 2 | 18.4  ± 4.0 | 0.554 | 15 | 35.9  ± 26.2 | 0.383 | 11 | 45.1  ± 45.6 | 0.200 |
| KOR0148 | ANOVA  F = 31.534 | 0.000* | 42 | 21.0  ± 20.7 | 84 | 3.1  ± 2.6 | 0.000* | 61 | 6.2  ± 12.2 | 0.000* |  |  |  |  |  |  |
