# Supplementary material for: Habitat use of loggerhead (Caretta caretta) and green (Chelonia mydas) turtles at the northern limit of their distribution range of the Northwest Pacific Ocean
Source: PLoS One. 2024 Apr 4;19(4):e0290202. doi: 10.1371/journal.pone.0290202 (PMC10994308; doi:10.1371/journal.pone.0290202)
Supplement: Attachment — Submitted filename: PONE-D-23-24778_R1Lee_Cooper_edited.pdf [file pone.0290202.s005.pdf]

PLOS ONE

Habitat use of loggerhead (Caretta caretta) and green (Chelonia mydas) turtles in the northern limit of their distribution range of the Northwest Pacific Ocean

--Manuscript Draft--

Manuscript Number:	PONE-D-23-24778R1
Article Type:	Research Article
Full Title:	Habitat use of loggerhead (Caretta caretta) and green (Chelonia mydas) turtles in the northern limit of their distribution range of the Northwest Pacific Ocean
Short Title:	Habitat use of loggerhead and green turtles in the Northwest Pacific Ocean
Corresponding Author:	Yong-Rock An, Dr.<br>National Marine Biodiversity Institute of Korea<br>Seocheon-gun, KOREA, REPUBLIC OF
Keywords:	sea turtle, Satellite tracking, loop behavior, habitat environment, foraging area
Abstract:	Verifying habitats, including the foraging and nesting areas for sea turtles, enables an understanding of their spatial ecology and successful planning of their conservation and management strategies. Recently, the observation frequency and bycatch of loggerhead (Caretta caretta) and green (Chelonia mydas) turtles have increased in the northern limit of their distribution range, in the northern part of the East China Sea and East (Japan) Sea. We conducted satellite tracking to investigate the habitat use of seven loggerhead and eight green turtles from June 2016 to August 2022 in this area, where little is known about their spatial ecology. We applied a 50 percent volume contour method to determine their main foraging areas and analyzed 6 environmental variables to characterize their habitats. Loggerhead turtles mainly stayed in and used the East China Sea as a foraging area during the tracking period, while two individuals among them also used the East Sea as a seasonal foraging area. Most green turtles also used the East China Sea as a foraging area, near South Korea and Japan, with one individual among them using the lower area of the East Sea as a seasonal foraging area. Notably, one green turtle traveled to Hainan Island in the South China Sea, a historical nesting area. Our results showed that the two sea turtle species included the East Sea as a seasonal foraging area, possibly owing to the abundance of food sources available, despite its relatively lower sea temperature. Considering that loggerhead and green sea turtles were observed using the northern part of the East China Sea and East Sea more frequently than previously known and that the sea temperature gradually increases due to climate change, conservation and management activities are required for sea turtles in these areas.
Order of Authors:	Il-Hun Kim, Dr.
	Il-Kook Park, Dr.
	Daesik Park, Dr.
	Min-Seop Kim, Dr.
	In-Young Cho, master
	Dongwoo Yang, Dr.
	Dong-Jin Han
	Eunvit Cho
	Won Joon Shim, Dr.
	Sang Hee Hong, Dr.
	Yong-Rock An, Dr.
Opposed Reviewers:	Yoshimasa Matsuzawa<br>Sea turtle Association of Japan<br>ymatsu0909@gmail.com<br>He is the president of the Japan Sea Turtle Association and a member of the IUCN's

Powered by Editorial Manager® and ProduXion Manager® from Aries Systems Corporation

	marine turtle specialist group. He has conducted a variety of studies on sea turtles in the North Pacific.
	Dae Yeon Moon, Dr.<br>president, Ocean and Fish Research<br>dymoon1001@hotmail.com<br>He is a PhD in sea turtle physiology. He has conducted numerous studies on the physiology and ecology of sea turtles. He is a former high-ranking government official in South Korea, where he established policies to protect sea turtles.
	George H. Balazs, Dr.<br>president, Pacific Islands Fisheries Science Center<br>ItsaHonuWorldinHawaii@hatmail.com<br>Golden Honu Services of Oceania<br>China/Hawaii Aloha Alliance
	David P. Robinson, Dr.<br>Jumeirah Group(Dubai)<br>snarkwatcharabia@gmail.com<br>This researcher has conducted mane study on sea turtle using satellite telemetry method. and published many valuable research about sea turtle on Plos One.
	Hideo Hatase, Dr.<br>The University of Tokyo Atmosphere and Ocean Research Institute: Tokyo Daigaku Taiki Kaiyo Kenkyujo<br>hatase@ori.u-tokyo.ac.jp<br>This researcher had conducted nany study on sea turtles. including satellite tracking, in Northwest Pacific Ocean. Which is the study site in this menuscript.
**Response to Reviewers:**	Thank you for your loyal review. We've implemented most of the suggestions made by both reviewers, and are responding to some of their comments. We look forward to your review again.
**Additional Information:**	
**Question**	**Response**
**Financial Disclosure**<br><br>Enter a financial disclosure statement that describes the sources of funding for the work included in this submission. Review the submission guidelines for detailed requirements. View published research articles from *PLOS ONE* for specific examples.<br><br>This statement is required for submission and **will appear in the published article** if the submission is accepted. Please make sure it is accurate.	This study was supported by grants from the National Marine Biodiversity Institute of Korea (2023M00300 and 2023E00300) and the 'Development of Technology for Impact Assessment of Marine Plastic Debris on Marine Ecosystem (EA0114)" funded by the Korea Institute of Ocean Science and Technology.<br><br>Author Contributions<br>Conceptualization: Il-Hun Kim, Il-Kook Park.<br>Data Curation: Il-Hun Kim, Il-Kook Park.<br>Formal Analysis: Il-Kook Park, Daesik Park.<br>Funding Acquisition: Yong Rock An. Min-Seop Kim, Won Joon Shim<br>Investigation: Il-Hun Kim, Min-Seop Kim, In-Young Cho, Dongwoo Yang, Dong-Jin Han, Eunvit Cho, Won Joon Shim, Sang Hee Hong<br>Methodology: Il-Kook Park, Daesik Park.<br>Project Administration: Yong Rock An.<br>Resources: Il-Hun Kim. Dong-Jin Han, Eunvit Cho<br>Software: Il-Kook Park.<br>Supervision: Daesik Park, Yong Rock An.<br>Validation: Daesik Park.<br>Visualization: Il-Kook Park.<br>Writing - Original Draft: Il-Hun Kim, Il-Kook Park, Daesik Park.<br>Writing – Review & Editing: Il-Hun Kim, Il-Kook Park, Daesik Park.<br><br>The three directors who secured the Funders participated in the project administration, supervision and investigation. They contributed to the secure and release of sea turtles and data management.

Powered by Editorial Manager® and ProduXion Manager® from Aries Systems Corporation

**Unfunded studies**<br>Enter: *The author(s) received no specific funding for this work.*<br><br>**Funded studies**<br>Enter a statement with the following details:<br>• Initials of the authors who received each award<br>• Grant numbers awarded to each author<br>• The full name of each funder<br>• URL of each funder website<br>• Did the sponsors or funders play any role in the study design, data collection and analysis, decision to publish, or preparation of the manuscript?<br>• **NO** - Include this sentence at the end of your statement: *The funders had no role in study design, data collection and analysis, decision to publish, or preparation of the manuscript.*<br>• **YES** - Specify the role(s) played.<br><br>* typeset	
**Competing Interests**<br><br>Use the instructions below to enter a competing interest statement for this submission. On behalf of all authors, disclose any competing interests that could be perceived to bias this work—acknowledging all financial support and any other relevant financial or non-financial competing interests.<br><br>This statement is required for submission and **will appear in the published article** if the submission is accepted. Please make sure it is accurate and that any funding sources listed in your Funding Information later in the submission form are also declared in your Financial Disclosure statement.<br><br>View published research articles from *PLOS ONE* for specific examples.	The authors have declared that no competing interests exist.

**NO authors have competing interests**<br><br>Enter: *The authors have declared that no competing interests exist.*<br><br>**Authors with competing interests**<br><br>Enter competing interest details beginning with this statement:<br><br>*I have read the journal's policy and the authors of this manuscript have the following competing interests: [insert competing interests here]*<br><br>* typeset	
**Ethics Statement**<br><br>Enter an ethics statement for this submission. This statement is required if the study involved:<br><br>• Human participants<br>• Human specimens or tissue<br>• Vertebrate animals or cephalopods<br>• Vertebrate embryos or tissues<br>• Field research<br><br>Write "N/A" if the submission does not require an ethics statement.<br><br>General guidance is provided below. Consult the submission guidelines for detailed instructions. **Make sure that all information entered here is included in the Methods section of the manuscript.**	This is an opinion on the ethics of animal testing research.<br>According to the Act on Laboratory Animals, a laboratory animal is a "vertebrate animal used or kept for the purpose of animal experimentation.<br><br>Since this study focused on sea turtles that were rescued from incidental catches by fishing gears in Korean watersand treated, it is not applicable to the animal experiment following the Laboratory Animal Act. That the advance review of the Laboratory Animal Management Committee is not obtained.<br><br>However, the two species of sea turtles were managed by "institutions specialized in rescuing and treating marine animals (Aqua Planet Yeosu, Korea National Maritime Museum, etc.)" designated for post-rescue treatment under the Act on the Conservation and Management of Marine Ecosystems.<br><br>We hereby attach the written resolution of the Marine Animal Protection Committee regarding.<br>Committee(2017 ~ 2022). We have attached a translation of the 2018 letter for your review.<br>* In 2016, sea turtles were not designated as a protected species in South Korea.

Powered by Editorial Manager® and ProduXion Manager® from Aries Systems Corporation

**Format for specific study types**<br><br>**Human Subject Research (involving human participants and/or tissue)**<br>• Give the name of the institutional review board or ethics committee that approved the study<br>• Include the approval number and/or a statement indicating approval of this research<br>• Indicate the form of consent obtained (written/oral) or the reason that consent was not obtained (e.g. the data were analyzed anonymously)<br><br>**Animal Research (involving vertebrate animals, embryos or tissues)**<br>• Provide the name of the Institutional Animal Care and Use Committee (IACUC) or other relevant ethics board that reviewed the study protocol, and indicate whether they approved this research or granted a formal waiver of ethical approval<br>• Include an approval number if one was obtained<br>• If the study involved *non-human primates*, add *additional details* about animal welfare and steps taken to ameliorate suffering<br>• If anesthesia, euthanasia, or any kind of animal sacrifice is part of the study, include briefly which substances and/or methods were applied<br><br>**Field Research**<br><br>Include the following details if this study involves the collection of plant, animal, or other materials from a natural setting:<br>• Field permit number<br>• Name of the institution or relevant body that granted permission	
**Data Availability**<br><br>Authors are required to make all data underlying the findings described fully available, without restriction, and from the time of publication. PLOS allows rare exceptions to address legal and ethical concerns. See the PLOS Data Policy and FAQ for detailed information.	Yes - all data are fully available without restriction

A Data Availability Statement describing where the data can be found is required at submission. Your answers to this question constitute the Data Availability Statement and **will be published in the article**, if accepted.<br><br>**Important:** Stating 'data available on request from the author' is not sufficient. If your data are only available upon request, select 'No' for the first question and explain your exceptional situation in the text box.<br><br>Do the authors confirm that all data underlying the findings described in their manuscript are fully available without restriction?	
**Describe where the data may be found in full sentences. If you are copying our sample text, replace any instances of XXX with the appropriate details.**<br><br>• If the data are **held or will be held in a public repository**, include URLs, accession numbers or DOIs. If this information will only be available after acceptance, indicate this by ticking the box below. For example: *All XXX files are available from the XXX database (accession number(s) XXX, XXX.).*<br>• If the data are all contained **within the manuscript and/or Supporting Information files**, enter the following: *All relevant data are within the manuscript and its Supporting Information files.*<br>• If neither of these applies but you are able to provide **details of access elsewhere**, with or without limitations, please do so. For example:<br><br>*Data cannot be shared publicly because of [XXX]. Data are available from the XXX Institutional Data Access / Ethics Committee (contact via XXX) for researchers who meet the criteria for access to confidential data.*<br><br>*The data underlying the results presented in the study are available from (include the name of the third party*	We have uploaded the sea turtle migration data to movebank as requested. We will compress the relevant files into a ZIP file and submit it to the Supporting Information - Compressed/ZIP File Archive section.<br><br>https://www.movebank.org/movebank/#page=studies,path=study3030282201

and contact information or URL).<br>• This text is appropriate if the data are owned by a third party and authors do not have permission to share the data.<br><br>* typeset	
Additional data availability information:	Tick here if the URLs/accession numbers/DOIs will be available only after acceptance of the manuscript for publication so that we can ensure their inclusion before publication.

Powered by Editorial Manager® and ProduXion Manager® from Aries Systems Corporation

[04 August 2023, 02 Nov 2023]

Miquel Vall-llosera Camps
Senior Editor
PLOS ONE

Dear Editor:

I wish to submit a research article for publication in *PLOS ONE*, titled "Habitat use of loggerhead (*Caretta caretta*) and green (*Chelonia mydas*) turtles in the Northwest Pacific Ocean, the northern limit of their distribution range." The paper was coauthored by Il-Hun Kim, Il-Kook Park, Daesik Park, Min-Seop Kim, In-Young Cho, Dongwoo Yang, Dong-Jin Han, Eunvit Cho, Won Joon Shim, and Sang Hee Hong.

This study investigates the travel patterns and home range of two species, the loggerhead (*Caretta caretta*) and green (*Chelonia mydas*) sea turtles, in the Northwest Pacific Ocean. We used satellite telemetry and the minimum convex polygon and 95 and 50 percent volume contour methods to identify the foraging, travel, and nesting areas of seven and eight loggerhead and green turtles, respectively. We observed that the two sea turtle species included the East Sea as a seasonal foraging area, possibly owing to the abundance of food sources available, despite its relatively lower water temperature.

We believe that our study makes a significant contribution to the literature because its results suggest that, promoted by a rise in sea temperatures due to climate change, the population of sea turtles that uses the East Sea during warm summers and autumns will increase in the following years. This prediction could be essential for the implementation of management strategies related to the conservation or restoration of sea turtles, which are endangered worldwide, in the North Pacific Ocean area.

This manuscript has not been published or presented elsewhere in part or in entirety and is not under consideration by another journal. We have read and understood your journal's policies, and we believe that neither the manuscript nor the study violates any of these. There are no conflicts of interest to declare.

The three directors who secured the Funders participated in the project administration, supervision and investigation. They contributed to the secure and release of sea turtles and data management.

Thank you for your consideration. I look forward to hearing from you.

Sincerely,
Yong-Rock An
Department of Ecology and Conservation, National Marine Biodiversity Institute of Korea, Seocheon, Chungcheongnam-do, Republic of Korea
rock@mabik.re.kr

Manuscript

Click here to access/download;Manuscript;(clean) manuscript_Habitat_use_of_two_sea_turtle_species.docx

Habitat use of loggerhead (*Caretta caretta*) and green (*Chelonia mydas*) turtles in the northern limit of their distribution range of the Northwest Pacific Ocean

Short title: Habitat use of loggerhead and green turtles in the Northwest Pacific Ocean

Il-Hun Kim[1¶], Il-Kook Park[2¶], Daesik Park[2], Min-Seop Kim[1], In-Young Cho[1], Dongwoo Yang[1], Dong-Jin Han[3], Eunvit Cho[3], Won Joon Shim[4], Sang Hee Hong[4,5], Yong-Rock An[1*]

[1] Department of Ecology and Conservation, National Marine Biodiversity Institute of Korea, Seocheon, Chungcheongnam-do, Republic of Korea

[2] Division of Science Education, Kangwon National University, Chuncheon, Gangwon-do, Republic of Korea

[3] Aqua Team, Aqua Planet Yeosu, Yeosu, Jeollanam-do, Republic of Korea

[4] Ecological Risk Research Department, Korea Institute of Ocean Science and Technology, Geoje, Gyeongsangnam-do, Republic of Korea

[5] Department of Ocean Science, University of Science and Technology, Daejeon, Republic of Korea

*Corresponding author

E-mail: rock@mabik.re.kr (Y-RA)

[¶]These authors contributed equally to this work.

Abstract

Verifying habitats, including the foraging and nesting areas for sea turtles, enables an understanding of their spatial ecology and successful planning of their conservation and management strategies. Recently, the observation frequency and bycatch of loggerhead (*Caretta caretta*) and green (*Chelonia mydas*) turtles have increased in the northern limit of their distribution range, in the northern part of the East China Sea and East (Japan) Sea. We conducted satellite tracking to investigate the habitat use of seven loggerhead and eight green turtles from June 2016 to August 2022 in this area, where little is known about their spatial ecology. We applied a 50 percent volume contour method to determine their main foraging areas and analyzed 6 environmental variables to characterize their habitats. Loggerhead turtles mainly stayed in and used the East China Sea as a foraging area during the tracking period, while two individuals among them also used the East Sea as a seasonal foraging area. Most green turtles also used the East China Sea as a foraging area, near South Korea and Japan, with one individual among them using the lower area of the East Sea as a seasonal foraging area. Notably, one green turtle traveled to Hainan Island in the South China Sea, a historical nesting area. Our results showed that the two sea turtle species included the East Sea as a seasonal foraging area, possibly owing to the abundance of food sources available, despite its relatively lower sea temperature. Considering that loggerhead and green sea turtles were observed using the northern part of the East China Sea and East Sea more frequently than previously known and that the sea temperature gradually increases due to climate change, conservation and management activities are required for sea turtles in these areas.

Introduction

Although many regional sea turtle populations are increasing thanks to conservation efforts [1,2], still six out of seven known sea turtle species are threatened worldwide, which has promoted conservation and restoration efforts from many countries and global organizations, such as the Convention on International Trade in Endangered Species of Wild Fauna and Flora (CITES) and the International Union for Conservation of Nature (IUCN), are trying to conserve and restore them. Understanding the spatial and foraging ecology of sea turtles is essential for establishing successful conservation and management strategies [2-5]. However, because sea turtles have a wide distribution range, diverse habitat types, and a complex life cycle, it is necessary to invest considerable time and effort to thoroughly understand their ecology [6-8]. Currently, considering that the distribution range of marine creatures, including sea turtles, is expanding polewardnorth owing to climate change [9-12], research on the northern edge of the sea turtle range is urgently needed. but currently insufficient. Satellite telemetry is a useful method for investigating the spatial ecology and habitat use of sea turtles, applied in various regions and species [6,13-16]. Although various molecular ecology techniques have been recently used to study population genetic diversity and determine the studying origin of sea turtles [17-21], satellite telemetry remains a powerful study method, particularly for the distribution boundary of sea turtles, which have very low population densities [5,14,16,22]. Because sea turtles show seasonal differences in habitat use, such as for foraging and nesting, and high habitat fidelity [23-26], satellite telemetry can effectively identify the major areas of these habitats [14,27,28]. Furthermore, analyzing these environmental characteristics of the identified habitats could reveal the factors affecting the habitat preferences of sea turtles, leading to proper conservation and management strategies against climate change and anthropogenic developments [29,30]. Overall, the habitat information and environmental characteristics identified by satellite telemetry could contribute to the effective protection and restoration of sea turtle populations [31].

In the Northwest Pacific Ocean, Loggerhead (*Caretta caretta*) and green (*Chelonia mydas*) turtles are the most commonly observed sea turtles and use it as foraging and nesting regions [32-34]. Sea turtle research in the Northwest Pacific Ocean has focused on the southern coastline of Japan and the southern Ryukyu Islands, where the main foraging and nesting sites of loggerhead and green turtles are located [19,35-37]. Recently, the nesting frequencies of loggerhead and green turtles are declining as a consequence of coastal development and other unknown factors in these areas [18,24,25]. Nevertheless, the observation frequency of loggerhead and green

turtles has increased in their northern distribution boundary region, including the northern parts of the East China Sea and East (Japan) Sea, near the coastline of the Korean Peninsula [34,38,39]. Despite the increased observation of loggerhead and green turtles at these distribution margins, studies on their habitat use are lacking. Several studies have shown that juvenile loggerhead turtles use the East Sea near South Korea and Japan as a route to the open Pacific Ocean, but few studies have been conducted on adult specimens [5,16,40]. In green turtles, some research showed that adults use the sea near Jeju Island [41], whereas juveniles use the Yellow Sea west of the Korean Peninsula [16]. However, the detailed patterns of habitat use by adults have not been studied yet.

In the present study, we aimed to reveal the travel patterns, habitat use, and environmental characteristics of the habitats of subadult and adult loggerhead and green turtles using satellite telemetry. Considering that the habitat use patterns of the two sea turtle species at the northern boundary of their distribution range are not well known, this study is expected to provide essential information about their spatial biology, which can be useful for the effective management and conservation of both sea turtle species in the Northwest Pacific Ocean.

Materials and methods

Satellite telemetry

From October 2015 to October 2022, we tracked 15 rehabilitated sea turtles (7 loggerhead and 8 green turtles), which were caught incidentally and rescued from fishing nets near the coastal area of the Republic of Korea (Table 1; S1 Table). Permissions for the sea turtle conservation program were given by the Marine Animal Protection Committee of Ministry of Oceans and Fisheries with whom this work was conducted. We measured the curved carapace length (CCL) and body weight (BW) of each sea turtle. Considering the minimum CCL of sexually mature sea turtles is known as 73 cm in loggerhead turtles and 67 cm in green turtles [42,43], 12 turtles were identified as adults, and three turtles as potential subadults (KOR-1, KOR-2, and KOR0155). Other than KOR0149, a male loggerhead turtle, the remaining 14 turtles were all female. The turtles were released between June and October at the coasts of Jeju Island (N 33.2449°, E 126.4128°), Yeosu (N 34.6306°, E 127.7935°), and Busan (N 35.1587°, E 129.1608°) in the Republic of Korea, where the threat of collision with ships was relatively low and the water temperature was relatively warm (16 - 28°C in SST).

To track sea turtles, we used the Argos satellite transmitters (Wildlife Computers Inc., Redmond, WA, USA), SPOT-352A (before September 2019, lifespan: approximately 920 days; 72 × 56 × 22 mm; 129 g), and SPOT-375A (after August 2021, lifespan: 915 days; 99 × 55 × 21 mm; 152 g). The Argos transmitter has been widely used for satellite tracking of sea turtles until recently [15, 16, 22, 44], although it could overestimate space use of sea turtles compared to more accurate approaches such as Fastloc-GPS [45,46]. We attached transmitters to the carapaces of each sea turtle using Aqua Mend Epoxy (PSI-Polymer Systems, NC, USA) and instant mix epoxy (Loctite, CT, USA). Before attaching transmitters, we cleaned the carapace using abrasive sandpaper to remove debris, wiped the carapace with isopropyl alcohol to remove greases [47,48]. To confirm the attachment stability of the transmitter to sea turtles, individuals were monitored for 24 h in an aquarium before release. The tracking period was defined as the period from the first tracking day after the release date to the last day of if the transmitter signal is not observed for two months from the date of the last signal received. Tracking data were downloaded from the ARGOS system (CLS America, http://www.clsamerica.com). The accuracy estimation of the ARGOS system was classified into six categories: class 0, with an error radius > 1,500 m; class 1, with > 1,000 m; class 2, with > 500 m; class 3, with > 150 m; and classes A and B, with no estimated accuracy. We only used the higher accuracy data for a given day among the recorded daily points, excluding classes A and B. We excluded location data on land without a distinct signal from the transmitter or long-distance travel of over 100 km in a single day, which is impossible for sea turtles to travel [15,16]. Although LC 0, which has lower location quality, could overestimate home range size, the reduction in the number of location points due to the exclusion of LC 0 obscures the understanding the space use patterns of sea turtles [46]. Because the aim of this study was to focus on space use patterns in little-known areas rather than determining the detailed home range size of sea turtles, we included LC 0 for analysis. Additionally, reassessment of location accuracy is necessary considering studies showing that LC A has higher accuracy than LC 0 [45,49].

Travel pattern and home range

We measured the distance that sea turtles traveled as the shortest straight line between two consecutively tracked location points in order to study their travel habits. The daily distance traveled was calculated as the distance between the given point and the point on the previous day at the most similar time as possible. In

addition, the total distance traveled by each turtle was calculated as the sum of the total distances traveled during the entire tracking period.

To define the home range size of individual sea turtles during the tracking periods, we used the minimum convex polygon (MCP) and 95 and 50 percentage volume contours (PVC) based on kernel density estimation, often used to calculate the home range of sea turtles [14,16,41,50]. The MCP and 95 PVC represent general home range sizes, while the 50 PVC indicates the core area of each individual. The distances traveled and home ranges were calculated, after which tracked turtle travel maps were generated using ArcGIS v. 10.1 (ESRI, Redlands, California, USA).

Habitat use

We compared the DDT of location data inside and outside 50 PVC, and defined the 50 PVC as a foraging area if the DDT inside was significantly smaller, based on the slow travel speed of sea turtles in their foraging areas [51,52]. On the other hand, we defined all areas outside 50 PVC and where DDT was not significantly small as transitional areas. We also defined 50 PVC nesting areas for adult turtles, that stayed near areas where nesting was previously recorded during their known breeding period. We identified all location points as either foraging, nesting, or transitional areas.

To understand the environmental characteristics of the habitat used by sea turtles, we collected and analyzed data regarding six environmental variables at each location, that are often used for sea turtle studies [14,50,53]: distance to shoreline (DTS, km), bathymetry (m), sea surface temperature (SST, °C), sea surface salinity (SSS, ‰), chlorophyll-a (Chla, mg/m^3), and particulate organic carbon (POC, mol/m^3) (Table 2). Distance to shore and bathymetry represent the topographical characteristics of the location where turtles were present, whereas SST and SSS are related to sea surface conditions at each location. Chlorophyll-a and POC, as indicators of the food source such as seagrass, are related to food abundance [29,54]. Also, we presented the whole tracking data and following environmental variable changes together on the four longest-tracked sea turtles (two loggerhead and two green turtles) and on the one farthest-traveled green turtle to show the relationship between environmental variables along the travel and habitat use of sea turtles. The six environmental variables were extracted using SeaDAS v. 8.3.0 (https://seadas.gsfc.nasa.gov/) and ArcGIS v. 10.1 (ESRI, Redlands, California, USA). Graphs

were created using the ggplot2 package [55] in R v. 4.0.5 software [56].

Statistical analysis

To distinguish between the foraging or nesting area and the transitional area, we used an independent t-test, a one-way ANOVA test, and the Kruskal-Wallis test. First, we conducted the Shapiro-Wilkinson test with DDT value of each sea turtle for normality. When only one 50 PVC was generated in a sea turtle individual, the inside and outside DDT were compared using a t-test. If a sea turtle has multiple 50 PVCs, we conducted one-way ANOVA or Kruskal-Wallis test on the inside and outside of each 50 PVC. Whenever significant differences were detected, we defined only 50 PVC that had significantly small DDT compared to the outside as the foraging or nesting area by the Bonferroni or Dunn post hoc test.

Based on the environmental variables obtained for the 50 PVC areas, we identified the preferred foraging environment characteristics of both sea turtle species. Additionally, we used an independent sample t-test to compare the interspecific differences in the variables between different foraging areas and the differences in variables between the foraging and transitional areas of loggerhead and green turtles. We considered it statistically significant when the p-value was under 0.05. Statistical analyses were conducted using SPSS v. 23.0 (IBM Corp., Armonk, NY, USA).

Results

Satellite telemetry

During the tracking period, 7 loggerhead turtles (6 adults and 1 subadult), with a mean CCL of 76.9 ± 6.9 cm and BW of 52.6 ± 13.8 kg, were tracked for 149.5 ± 154.2 days, traveling between 25.1° N, 123.9° E to 39.3° N, 135.5° E (Table 1). The mean daily distance traveled by loggerhead turtles was 20.7 ± 9.5 km, while the total distance traveled was 3,083.4 ± 3,297.3 km (range: 129.8 – 8,964.8). The mean home range size of loggerhead turtles was 181,342 ± 205,355 km^2 by MCP and 838 ± 118,402 km^2 by 95 PVC, while the core habitat area was 11,703 ±

11,725 km^2 by 50 PVC. The mean values of the six environmental variables in the foraging areas of the loggerhead turtles were 54.3 km of DTS, 236.6 m of bathymetry, 22.0°C of SST, 33.2‰ of SSS, 0.64 mg/m^3 of Chla, and 138.4 mol/m^3 of POC (Table 3).

During the tracking duration, 8 green turtles (6 adults and 2 subadults), with a mean CCL of 74.2 ± 10.9 cm and BW of 59.7 ± 26.7 kg, were tracked for 187.2 ± 148.4 days, with movements ranging from 15.4° N, 106.9° E to 37.5° N, 131.2° E (Table 1). The mean daily distance traveled by green sea turtles was 23.5 ± 12.5 km, while the total distance traveled was 2,691.3 ± 2,004.0 km (range: 1,291.7 – 6,484.3). The mean home range size of green turtles was 243,329 ± 336,756 km^2 by MCP and 112,031 ± 96,626 km^2 by 95 PVC, and the core habitat area was 12,323 ± 10,466 km^2 by 50 PVC. The mean values of the environmental variables in the foraging areas of green turtles were 7.8 km of DTS, 54.8 m of bathymetry, 22.8°C of SST, 33.1‰ of SSS, 0.59 mg/m^3 of Chla, and 104.6 mol/m^3 of POC (Table 3).

Seasonal travel pattern

Seven loggerhead turtles were released in South Korea at 33.2° N – 34.6° N during summer and autumn, from June to September (Table 1). Five loggerhead turtles traveled southward from the release location and stayed below 35° N throughout the tracking period, whereas KOR0092 and KOR0151 traveled up to the East Sea at 39°N during the summer and autumn, between July and early December (Fig 1). Eight green turtles were released in South Korea at 33.2° N – 35.2° N during summer and autumn, from August to October (Table 1). Similar to the loggerhead turtles, seven green turtles moved southward from the release location and stayed below 35° N. Only KOR0009 traveled to the East Sea up to 37.5° N during the spring and summer, between April and July (Fig 1).

Habitat use and environmental variables

Loggerhead turtles used areas in the sea near Okinawa Island (KOR0008), Tsushima Islands (KOR0092), the East China Sea close to Jeju Island (KOR0091 and KOR0092), and the east coastal sea of the Gangwon Province in South Korea (KOR0151) for foraging (Fig 2; S2 Table). No loggerhead turtles were presumed to have used the

nesting areas during the tracking period because the areas they used had no previous nesting records. Okinawa Island, which is the only known nesting area, was only used during the non-breeding season. In foraging areas, loggerhead turtles traveled shorter distance per day (p-value < 0.05) and stayed closer to the shore than during transitional periods (p-value < 0.001). In addition, the foraging sites of loggerhead turtles had lower SST (p-value < 0.05), higher SSS (p-value < 0.05), and lower POC (p-value < 0.05) values than of transitional areas (Table 3).

The major foraging areas for four green turtles were identified. KOR-2 used the sea close to Tanega Island, while KOR0003 used the sea close to Jeju Island. KOR0148 used the sea near Jeju Island in South Korea and Uji Island, and KOR0009 used three foraging areas in the sea near Yeosu and Ulsan, and the East China Sea, west of Kyushu in Japan (Fig 3; S2 Table). Furthermore, the nesting areas of KOR0104 were located near Hainan Island, a historical nesting site for green turtles [57,58]. The daily distance traveled (p-value < 0.05) and DTS (p-value <0.05) of green turtles were larger in their transitional areas than those in their foraging areas (Table 3). Considering the bathymetry values, green turtles used shallower areas for foraging than that used for travel (p-value < 0.05). There was no significant difference in SST between the transitional and foraging areas (p-value < 0.05), whereas SSS was higher in transitional areas than that in foraging areas (p-value < 0.05). Both Chla (p-value < 0.05) and POC (p-value < 0.05) were higher during travel than during foraging.

In the foraging areas used by loggerhead (n = 408) and green turtles (n = 545), the observed variables corresponding to loggerhead turtles showed a longer average daily distance traveled (p-value < 0.05) and larger DTS (p-value < 0.05), deeper bathymetry (p-value < 0.05), lower SST (p-value < 0.05), lower SSS (p-value < 0.05), and higher Chla (p-value < 0.05) and POC (p-value < 0.05; Table 4) than those corresponding to green turtles (Table 3). Also, we represented the monthly changes in foraging areas of the six variables for both sea turtles (Fig. 4).

Representative habitat use pattern

KOR0092 (329 days) and KOR0151 (401 days), which were the longest-tracked loggerhead turtles, gave us the most detailed information about the environment in the foraging area (Fig. 5). Loggerhead turtle KOR0092 first stayed in the sea south of Jeju Island, between December 10, 2018 and April 17, 2019. After release, KOR0092 moved to and stayed at the sea near Tsushima Island, from April 23, 2019 to May 23, 2019, and from June 7, 2019 to July 22, 2019. Its first major foraging area was located in the open East China Sea, more than 100 km away

from land during winter, and with an average of 0.54 mg/m^3 of Chla and 129.2 mol/m^3 of POC. The second major foraging area of KOR0092 was located near Tsushima Island, mainly during the spring and summer (Fig 5). Immediately after its release, KOR0151 moved to the coast sea east of the Gangwon Province between autumn and early winter. At the start of winter, KOR0151 traveled southward toward Kago Island and used the sea in southern Japan until the end of winter. KOR0151 stayed near the eastern sea of Jeju Island for two months in spring, after which it returned to the eastern coast sea of the Gangwon Province and used it for three months from summer until the end of tracking in autumn.

Compared to loggerhead turtles, green turtles showed wider travel ranges in the sea around South Korea, Japan, China, and Vietnam (Fig 1). Among the eight green turtles, KOR0009 was tracked for the longest period (365 days) and showed three seasonal habitats. Green turtle KOR0009 used three core areas: the coastal sea of Yeosu in South Korea, in autumn (from October 7, 2017 to October 30, 2017 and later from August 4, 2018 to September 28, 2018), the East China Sea in winter (from November 7, 2017 to December 8, 2017 and later from January 15, 2018 to February 22, 2018), and the coastal sea near Ulsan, Gyeongnam Province in South Korea, in spring and summer (from March 24, 2018 to April 10, 2018, from May 24, 2018 to June 5, 2018, and from July 9, 2018 to July 25, 2018). After its release at Jeju Island, KOR0009 used the coastal sea near Yeosu in autumn and traveled to the East China Sea, located at the lowest latitude for overwintering and where the Chla (0.28 mg/m^3) and POC (76.9 mol/m^3) were the lowest. After winter, KOR0009 traveled to the coastal sea of Ulsan, which had 1.26 mg/m^3 of Chla and 217.8 mol/m^3 of POC, where it stayed between spring and summer. Finally, KOR0009 returned to the coastal sea of Yeosu in autumn, where it stayed until the end of the tracking.

KOR0104 was tracked for 206 days and showed a distinct travel pattern reaching Vietnam and Hainan Island, which represents the farthest distance traveled during this study (Fig 6). Upon release in Yeosu, KOR0104 continuously traveled to the South Central Coast of Vietnam for 100 days until January 13, 2019, after which it traveled backward and arrived at Hainan Island, China. The core habitat of KOR0104 was located near the northwest coast of Hainan Island, where it stayed from February 26, 2019, to April 3, 2019. Its core habitat had 7.2 m of bathymetry, 26°C of SST, 2.30 mg/m^3 of Chla, and 202.0 mol/m^3 of POC, and an average distance from the shoreline of 1.2 km (Fig 6).

Discussion

Habitat use of loggerhead turtle

In this study, loggerhead turtles used the open East China Sea, the coastal sea adjacent to the southern Korean Peninsula and western Japan, and the East (Japan) Sea as their main foraging areas, including the coastal seas of Okinawa Island, Jeju Island, Tsushima Island, and Gangwon Province. Japan has the largest nesting area for loggerhead turtles in the North Pacific Ocean. Major foraging areas adult turtles are located in the seas around Japan, including the East China Sea, the sea west of the Ryukyu Islands, the open North Pacific Ocean, and the southern coastal area of the Japanese Archipelago [24,59]. However, in the present study, the seven tracked loggerhead turtles mainly used the northern part of the East China Sea as foraging areas, which have higher latitudes than those of previously reported areas, such as those near Jeju Island, Tsushima Island, and the east coast of South Korea. These results indicate that the foraging range of loggerhead turtles in the western Northwest Pacific Ocean is wider than previously reported.

All seven loggerhead turtles mainly used the northern part of the East China Sea, which is a suitable habitat for the species owing to being shallow, with a sea depth of less than 100 m and having an annual SST over 20 °C [5,60]. Additionally, in this region, small islands such as Jeju Island, Tsushima Island, and Okinawa Island provide core foraging areas for loggerhead turtles [5,22,61,62]. Furthermore, despite the few records of loggerhead turtles in the East Sea, KOR0092 and KOR0151 periodically used the East Sea in this study. These two turtles showed a foraging loop behavior, using the East Sea in summer and autumn when the SST was higher and the southern East China Sea in winter when the SST was lower. KOR0092 and KOR0151 traveled south in late November and early December and back to the East Sea in the summer. Despite the East Sea having a depth of less than 1,000 m and a relatively low SST, its Chla and POC contents were high, possibly because of an abundance of food sources. Considering such environmental conditions, KOR0092 and KOR0151 might consume enough food in the East Sea during the warm summer and early autumn seasons and then travel to the southern East China Sea for overwintering in the cold winter when the SST drops. Loggerhead turtles use the East Sea near the eastern shoreline of South Korea to forage various seaweeds, jellyfish, and shellfish [63]. In a previous report, over 90% of the loggerhead turtles studied near the Korean Peninsula, with a higher latitude and a lower SST than those of southern Japan, were observed between summer and autumn, between June and November [34]. The main reason

for the observed pattern is thought to be related to low sea temperatures. The coastal sea of South Korea has an SST below 15 °C from December to May [64]; as loggerhead turtles are vulnerable to this sea temperature, they are rarely observed [22,36,65]. Currently, with the sea temperature of the Pacific Ocean continuously rising largely due to climate change, the amount of food sources in the traditional foraging areas of loggerhead turtles is decreasing, forcing them to exploit cooler foraging areas that have more available food sources [9]. As sea temperatures in the Northwest Pacific Ocean increase further, more loggerhead turtles are expected to stay longer in the East Sea and use it as a foraging area.

Habitat use of green turtles

Except for KOR0104, which traveled to Hainan Island, green turtles mainly used the East China Sea between South Korea and Japan, the coastal sea of the Korean Peninsula, and small islands in southern Japan as foraging areas. The major foraging areas of green turtles in the Northwest Pacific Ocean are the coastal sea of southern Japan and the Ryukyu Islands [37]; however, in this study, among the eight turtles, only KOR-1 and KOR0004 traveled to the Ryukyu Islands. The remaining five green turtles, except for KOR0104, used the northern East China Sea as their major foraging area. Tanega Island is a traditional foraging or nesting area for green turtles [60], which was used in the present study by the subadult KOR-2 as an immature foraging area. Jeju Island, which is also a major foraging area for green turtles [34,41], was used by two green turtles, KOR0003 and KOR0148, as their major foraging area in this study. Moreover, they seemed to also use it as an overwintering area considering they stayed there in autumn and winter. Comparable to our results, five of eight green turtles studied by Jang et al. (2018) mainly stayed near Jeju Island during the tracking period, including during winter [41]. These results suggest that green turtles use the upper East China Sea, including the seas near Jeju Island and the southern Korean Peninsula, as both foraging and overwintering areas.

Adult green turtles can have high habitat fidelity for more than one foraging area [4,26,66]. In particular, KOR0009 showed typical foraging loop behavior in three seasonal habitats. Immediately after release, KOR0009 stayed in the sea neighboring of Yeosu in South Korea, in autumn, used the East China Sea at the lowest latitude during winter, used the sea around Ulsan, Gyeongnam Province in South Korea, at the highest latitude during summer, and returned to Yeosu in autumn. Rather than the loggerhead turtles, green turtles have also been reported to use the East Sea near the eastern shoreline of the Korean Peninsula mainly to forage seaweeds [63]. Among the

three habitats, food-related Chla and POC were higher in the sea near Yeosu and Ulsan at high latitudes and relatively low in the East China Sea. Therefore, like loggerhead turtles KOR0092 and KOR0151, green turtle KOR0009 also maintained in the coastal area of South Korea, which provides abundant food sources despite its relatively low sea temperatures, and traveled to the southern East China Sea before winter, when its thermoregulation is limited. Additionally, green turtles consume animal matter more than seagrass at high latitudes or low SST areas under 25°C [67]. However, although green turtles generally used areas below SST of 23°C in our study, the most abundant food item was seaweed according to previous research about stomach contents [34]. This supports that green turtles use waters off South Korea as foraging areas in mainly warm season and travel to lower latitudes in the cold season.

KOR0104 was the only turtle that was presumed to have traveled to a nesting area, even though its destination, Hainan Island, has had no records of green turtle nesting in the past 37 years owing to coastal development [57,58]. Nevertheless, KOR0104 visited 12 of the 13 historical nesting areas on Hainan Island [58] and appeared to aim to nest regardless of its success. The Yaeyama and Ogasawara Islands, which are closer to the release site, are the largest nesting sites for green turtles in the Northwest Pacific Ocean [68], so the long travel of KOR0104 to Hainan Island suggests that this turtle may have originated in the southwestern Pacific. Previous genetic analyses using mtDNA have shown that green turtles in the Northwest Pacific Ocean are intermixed with turtles from Southeast Asian colonies [19,20,68]. Other studies have also reported the Taiwan Strait being used by other green turtles for travel [14,69]. Therefore, this route could be a major passage for green turtles, connecting the northern and southern parts of the western Pacific Ocean.

Comparison of the two sea turtle species

Both the loggerhead and green turtles mainly stayed in the East China Sea, while a few individuals used the East Sea, mainly in summer and autumn. Green turtles showed a wider distribution and more diverse travel patterns than loggerhead turtles, with one green turtle travelling to the South China Sea. Additionally, differences in the environmental variables between the foraging habitats of the two species were identified. Throughout the year, green turtles stayed in foraging areas within 26 km of the shoreline and used the areas with depths of less than 100 m, while loggerhead turtles used areas with depths of approximately 150 m from winter to spring and over

1,000 m in summer and autumn in the East Sea. In addition, loggerhead turtles stayed approximately 50 km away from the shoreline during winter and summer. Both adult loggerhead and green turtles are known to forage mainly in shallow coastal waters, where the depths reach between 20 and 50 m [50,66], although some turtles prefer open oceanic habitats [4,70]. In Korean waters, more loggerhead turtles are observed in the East Sea, whereas more green turtles are observed near Jeju Island [5,34,41]. Because two loggerhead turtles, KOR0092 and KOR0151, stayed in the East Sea for long periods, the values of Chla and POC in the foraging areas of loggerhead turtles were greater than those of green turtles. These interspecific differences suggest that loggerhead turtles are relatively better adapted to deeper waters and cooler sea temperatures than green turtles [52].

Conservation strategy

The information obtained through the tracked sea turtles expands our horizons on the travel patterns and habitat use of them in the north part of the Northwest Pacific Ocean, where little is known about the spatial ecology of sea turtles. The home range of sea turtles could be overestimated considering the adaptation to the sea and migration distances to suitable habitats caused by their rehabilitation period in artificial facilities. Nevertheless, the periodic behavior of tracked sea turtles and their response to environmental variables, especially SST, Chla, and POC, showed that the rescued turtles were rehabilitated well and are adapting to the sea. Our results revealed patterns of habitat use different from those reported in previous results [24,37,59]. Contrary to what has been previously reported, adult sea turtles did not migrate to the open Central Pacific Ocean in our study [19,41], and instead stayed in and used the East China Sea and East Sea around South Korea and Japan as their main foraging areas. Notably, this study marks the first time the East Sea (east coast of the Gangwon Province in South Korea) has been identified as a sea turtle foraging area. The low sea temperatures in the northern part of the East China Sea and East Sea have been reported to limit the habitat use of loggerhead and green sea turtles [34,37]. However, recent climate change causes a continuous increase in sea temperatures in the North Pacific Ocean, which could extend northward into the habitat [9,10,71]. During the past 50 years, the sea temperature around South Korea has increased by approximately 1.5°C, which is near three times greater than the global average increase [72,73]. Although some sea turtles were observed using the East Sea during warm summers and autumns in this study, the number of sea turtles that do so is expected to rapidly increase in following years due to climate change. The appearance of sea turtles in the coastal sea of South Korea and the steady increase in the number of fishery bycatch

records over the last 15 years [34,38,39] support this prediction. Therefore, it is necessary to continuously update the occurrence pattern of sea turtles in these areas and identify potential problems that sea turtles will face following population increases. Given that fishery bycatch is responsible for a global decline of sea turtles [7,74,75], protection policies for sea turtles and monitoring plans for fishing vessels should be prepared and applied at a national level. In conclusion, the increased interest in and implementation of management policies for sea turtle habitats in the northern part of the East China Sea and the East Sea near the Korean Peninsula and Japan could play a critical role in the conservation of sea turtles in the near future.

Acknowledgments

We thank Min-Woo Park and Hyerim Kwon for their assistance with data management.

References

1. Hudgins JA, Hudgins EJ, Köhnk S, Mohamed Riyad E, Stelfox MR. A brighter future? Stable and growing sea turtle populations in the Republic of Maldives. PLoS One 2023;18: e0283973. doi: 10.1371/journal.pone.0283973

2. Mazaris AD, Schofield G, Gkazinou C, Almpanidou V, Hays GC. Global sea turtle conservation successes. Sci Adv. 2017;3: e1600730. doi: 10.1126/sciadv.1600730.

3. Hamann M, Godfrey MH, Seminoff JA, Arthur K, Barata PCR, Bjorndal KA, et al. Global research priorities for sea turtles: informing management and conservation in the 21st century. Endang Species Res. 2010;11: 245-269. doi: 10.3354/esr00279.

4. Shimada T, Aoki S, Kameda K, Hazel J, Reich K, Kamezaki N. Site fidelity, ontogenetic shift and diet composition of green turtles Chelonia mydas in Japan inferred from stable isotope analysis. Endang Species Res. 2014;25: 151-164. doi: 10.3354/esr00616.

5. Oki K, Hamabata T, Arata T, Parker DM, Ng CKY, Balazs GH. Inferred adult foraging grounds of two marine turtle species nesting at Amami-Oshima, Japan. Chelonian Conserv Biol. 2019;18: 91-97. doi: 10.2744/CCB-1337.1.

6. Hatase H, Sato K, Yamaguchi M, Takahashi K, Tsukamoto K. Individual variation in feeding habitat use by adult female green sea turtles (*Chelonia mydas*): are they obligately neritic herbivores? Oecologia. 2006;149: 52-64. doi: 10.1007/s00442-006-0431-2.

7. Lewison RL, Crowder LB, Wallace BP, Moore JE, Cox T, Zydelis R, et al. Global patterns of marine mammal, seabird, and sea turtle bycatch reveal taxa-specific and cumulative megafauna hotspots. Proc Natl Acad Sci U S A. 2014;111: 5271-5276. doi: 10.1073/pnas.1318960111.

8. Putman NF, Hawkins J, Gallaway BJ. Managing fisheries in a world with more sea turtles. Proc Biol Sci. 2020;287: 20200220. doi: 10.1098/rspb.2020.0220.

9. Chaloupka M, Kamezaki N, Limpus C. Is climate change affecting the population dynamics of the endangered Pacific loggerhead sea turtle? J Exp Mar Biol Ecol. 2008;356: 136-143. doi: 10.1016/j.jembe.2007.12.009.

10. Abraham JP, Baringer M, Bindoff NL, Boyer T, Cheng LJ, Church JA, et al. A review of global ocean temperature observations: implications for ocean heat content estimates and climate change. Rev Geophys. 2013;51: 450-483. doi: 10.1002/rog.20022.

11. Garcia RA, Cabeza M, Rahbek C, Araújo MB. Multiple dimensions of climate change and their implications for biodiversity. Science. 2014;344: 1247579. doi: 10.1126/science.1247579.

12. Butt N, Whiting S, Dethmers K. Identifying future sea turtle conservation areas under climate change. Biol Conserv. 2016;204: 189-196. doi: 10.1016/j.biocon.2016.10.012.

13. Hays GC, Hawkes LA. Satellite tracking sea turtles: Opportunities and challenges to address key questions. Front Mar Sci. 2018;5: 432. doi: 10.3389/fmars.2018.00432.

14. Evans DR, Carthy RR, Ceriani SA. Migration routes, foraging behavior, and site fidelity of loggerhead sea turtles (*Caretta caretta*) satellite tracked from a globally important rookery. Mar Biol. 2019;166: 134. doi: 10.1007/s00227-019-3583-4.

15. Robinson DP, Hyland K, Beukes G, Vettan A, Mabadikate A, Jabado RW, et al. Satellite tracking of rehabilitated sea turtles suggests a high rate of short-term survival following release. PLoS One. 2021;16: e0246241. doi: 10.1371/journal.pone.0246241.

16. Kim IH, Park IK, Han DJ, Kim MS, Park D, Moon DY, et al. Movement patterns of juvenile loggerhead turtles (*Caretta caretta* L. 1758) and green turtles (*Chelonia mydas* L. 1758) hatched in captivity and released in the Korean Waters. Animals (Basel). 2022;12: 2157. doi: 10.3390/ani12162157.

17. Bowen BW. Tracking marine turtles with genetic markers. BioScience. 1995;45: 528-534. doi: 10.2307/1312697.

18. Hamabata T, Nishida S, Kamezaki NI, Koike H 2009. Genetic structure of populations of the green turtle (*Chelonia mydas*) in Japan using mtDNA control region sequences. *Bulletin of the graduate school of social and cultural studies. Kyushu University*, 15, 35-50.

19. Nishizawa H, Naito Y, Suganuma H, Abe O, Okuyama J, Hirate K, et al. Composition of green turtle feeding aggregations along the Japanese archipelago: implications for changes in composition with current flow. Mar Biol. 2013;160: 2671-2685. doi: 10.1007/s00227-013-2261-1.

20. Yang W, Wang Y, Chen M. Genetic structure and diversity of green sea turtle (Chelonia mydas) from South China Sea inferred by mtDNA control region sequence. Biochem Syst Ecol. 2015;60: 95-98. doi: 10.1016/j.bse.2015.04.007.

21. Loisier A, Savelli MP, Arnal V, Claro F, Gambaiani D, Sénégas JB, et al. Genetic composition, origin and conservation of loggerhead sea turtles (*Caretta caretta*) frequenting the French Mediterranean coasts. Mar Biol. 2021;168: 1-15. doi: 10.1007/s00227-020-03815-6.

22. Saito T, Kurita M, Okamoto H, Uchida I, Parker D, Balazs G. Tracking male loggerhead turtle migrations around southwestern Japan using satellite telemetry. Chelonian Conserv Biol. 2015;14: 82-87. doi: 10.2744/ccab-14-01-82-87.1.

23. Sato K. Decline of the loggerhead turtle, Caretta caretta, nesting on Senri Beach in Minabe, Wakayama, Japan. Chelonian Conserv Biol. 1997;2: 600-603.

24. Hatase H, Takai N, Matsuzawa Y, Sakamoto W, Omuta K, Goto K, et al. Size-related differences in feeding habitat use of adult female loggerhead turtles *Caretta caretta* around Japan determined by stable isotope analyses and satellite telemetry. Mar Ecol Prog Ser. 2002;233: 273-281. doi: 10.3354/meps233273.

25. Shimada T, Jones R, Limpus C, Groom R, Hamann M. Long-term and seasonal patterns of sea turtle home ranges in warm coastal foraging habitats: implications for conservation. Mar Ecol Prog Ser. 2016;562: 163-179. doi: 10.3354/meps11972.

26. Balazs GH, Parker DM, Rice MR. Ocean pathways and residential foraging locations for satellite tracked green turtles breeding at French Frigate Shoals in the Hawaiian Islands. Micronesica. 2017;4: 1-19.

27. Polovina J, Uchida I, Balazs G, Howell EA, Parker D, Dutton P. The Kuroshio Extension Bifurcation Region: a pelagic hotspot for juvenile loggerhead sea turtles. Deep Sea Res II. 2006;53: 326-339. doi: 10.1016/j.dsr2.2006.01.006.

28. Kobayashi DR, Polovina JJ, Parker DM, Kamezaki N, Cheng IJ, Uchida I, et al. Pelagic habitat characterization of loggerhead sea turtles, *Caretta caretta*, in the North Pacific Ocean (1997–2006): insights from satellite tag tracking and remotely sensed data. J Exp Mar Biol Ecol. 2008;356: 96-114. doi: 10.1016/j.jembe.2007.12.019.

29. McCarthy AL, Heppell S, Royer F, Freitas C, Dellinger T. Identification of likely foraging habitat of pelagic loggerhead sea turtles (*Caretta caretta*) in the North Atlantic through analysis of telemetry track sinuosity. Prog Oceanogr. 2010;86: 224-231. doi: 10.1016/j.pocean.2010.04.009.

30. Mencacci R, De Bernardi E, Sale A, Lutjeharms JRE, Luschi P. Influence of oceanic factors on long-distance movements of loggerhead sea turtles displaced in the southwest Indian Ocean. Mar Biol. 2010;157: 339-349. doi: 10.1007/s00227-009-1321-z.

31. Hays GC, Bailey H, Bograd SJ, Bowen WD, Campagna C, Carmichael RH, et al. Translating marine animal tracking data into conservation policy and management. Trends Ecol. Evol. 2019;34: 459-473. doi: 10.1016/j.tree.2019.01.009.

32. Goris RC. Guide to the amphibian and reptiles of Japan. Florida: Krieger Publishing Company; 2004. pp. 131-136.

33. Chan SKF, Cheng IJ, Zhou T, Wang HJ, Gu HX, Song XJ. A comprehensive overview of the population and conservation status of sea turtles in China. Chelonian Conserv Biol. 2007;6: 185-198. doi: 10.2744/1071-8443(2007)6[185:ACOOTP]2.0.CO;2.

34. Kim IH, Moon DY, Cho IY, Kim MS, An YR, Han D, et al. Occurrence of sea turtles in the Korean Waters and the morphological characteristics of two major species. Korean J Fish Aquat Sci. 2017;50: 311-318. Doi: 10.5657/KFAS.2017.0311.

35. Hatase H, Matsuzawa Y, Sakamoto W, Baba N, Miyawaki I. Pelagic habitat use of an adult Japanese male loggerhead turtle *Caretta caretta* examined by the Argos satellite system. Fisheries Sci. 2002a;68: 945-947. doi: 10.1046/j.1444-2906.2002.00515.x.

36. Narazaki T, Sato K, Miyazaki N. Summer migration to temperate foraging habitats and active winter diving of juvenile loggerhead turtles *Caretta caretta* in the western North Pacific. Mar Biol. 2015;162: 1251-1263. doi: 10.1007/s00227-015-2666-0.

37. Hamabata T, Nishizawa H, Kawazu I, Kameda K, Kamezaki N, Hikida T. Stock composition of green turtles *Chelonia mydas* foraging in the Ryukyu Archipelago differs with size class. Mar Ecol Prog Ser. 2018;600: 151-163. doi: 10.3354/meps12657.

38. Moon DY, Jung MM, An YR, Choi SG, Oh BS, Kim ZG, et al. Distribution and strandings of endangered sea turtles in Korean Waters. Korean J Fish Aquat Sci. 2009;42: 657-663. doi: 10.5657/kfas.2009.42.6.657.

39. Jung MM, Moon DY, Kim SH, Kim HS, Kim JW. Observation and record of sea turtles in bycatch and stranding from Jeju Island of Korea. J Fish Mar Sci Educ. 2012;24: 662-669. doi: 10.13000/JFMSE.2012.24.5.662.

40. Saito T, Kurita M, Okamoto H, Kakizoe Y, Parker D, Briscoe D, et al. Satellite tracking immature loggerhead turtles in temperate and subarctic ocean habitats around the Sea of Japan. Micronesica. 2018;3: 1-20.

41. Jang S, Balazs GH, Parker DM, Kim BY, Kim MY, Ng CKY, et al. Movements of green turtles (*Chelonia mydas*) rescued from pound nets near Jeju Island, Republic of Korea. Chelonian Conserv Biol. 2018;17: 236-244. doi: 10.2744/CCB-1279.1.

42. Green D. Growth rates of wild immature green turtles in the Galapagos Islands, Ecuador. J Herpetol. 1993;27: 338-341. doi: 10.2307/1565159.

43. Ishihara T, Kamezaki N. Size at maturity and tail elongation of loggerhead turtles (Caretta caretta) in the North Pacific. Chelonian Conserv Biol. 2011;10: 281-287. doi: 10.2744/CCB-0893.1.

44. Robinson DP, Jabado RW, Rohner CA, Pierce SJ, Hyland KP, Baverstock WR. Satellite tagging of rehabilitated green sea turtles *Chelonia mydas* from the United Arab Emirates, including the longest tracked journey for the species. PLoS One. 2017;12: e0184286. doi: 10.1371/journal.pone.0184286.

45. Witt MJ, Åkesson S, Broderick AC, Coyne MS, Ellick J, Formia A, et al. Assessing accuracy and utility of satellite-tracking data using Argos-linked Fastloc-GPS. Anim Behav. 2010;80: 571. doi: 10.1016/j.anbehav2010.05.022.

46. Thomson JA, Börger L, Christianen MJA, Esteban N, Laloë JO, Hays GC. Implications of location accuracy and data volume for home range estimation and fine-scale movement analysis: comparing Argos and Fastloc-GPS tracking data. Mar Biol. 2017;164: 1-9. doi: 10.1007/BF00397131.

47. Balazs GH, Miya RK, Beavers S. Procedures to attach a satellite transmitter to the carapace of an adult green turtle, *Chelonia mydas*. NOAA Tech. Memorandum NMFS-SEFSC; 1996. pp. 21–26.

48. Seney, EE, Higgins BM & Landry Jr AM. Satellite transmitter attachment techniques for small juvenile sea turtles. J Exp Mar Biol Ecol. 2010;384: 61-67. doi: 10.1016/j.jembe.2010.01.002.

49. Hays GC, Åkesson S, Godley BJ, Luschi P, Santidrian P. The implications of location accuracy for the interpretation of satellite-tracking data. Anim Behav. 2001;61: 1035-1040.

50. Hart KM, Lamont MM, Fujisaki I, Tucker AD, Carthy RR. Common coastal foraging areas for loggerheads in the Gulf of Mexico: opportunities for marine conservation. Biol Conserv. 2012;145: 185-194. doi: 10.1016/j.biocon.2011.10.030.

51. Zbinden JA, Aebischer A, Margaritoulis D, Arlettaz R. Important areas at sea for adult loggerhead sea turtles in the Mediterranean Sea: satellite tracking corroborates findings from potentially biased sources. Mar Biol. 2008;153: 899-906. doi: 10.1007/s00227-007-0862-2.

52. Schofield G, Hobson VJ, Fossette S, Lilley MK, Katselidis KA, Hays GC. Fidelity to foraging sites, consistency of migration routes and habitat modulation of home range by sea turtles. Divers Distrib. 2010;16: 840-853. doi: 10.1111/j.1472-4642.2010.00694.x.

53. Coyne MS, Godley BJ. Satellite Tracking and Analysis Tool (STAT): an integrated system for archiving, analyzing and mapping animal tracking data. Mar Ecol Prog Ser. 2005;301: 1-7. doi: 10.3354/meps301001.

54. Evrard V, Kiswara W, Bouma TJ, Middelburg JJ. Nutrient dynamics of seagrass ecosystems: 15N evidence for the importance of particulate organic matter and root systems. Mar Ecol Prog Ser. 2005;295: 49-55. doi: 10.3354/meps295049.

55. Wickham H. ggplot2: elegant graphics for data analysis. Springer; 2016.

56. R Core Team. R: A language and environment for statistical computing. R Foundation for Statistical Computing; 2021.

57. Wang YM. Achievement and perspective of the researches on South China Sea turtle resources and protection in China. Chin J Ecol. 1993;12: 60-61.

58. Zhang T, Lin L, Li M, Kong L, Wang J, Shi HT. Investigation on beach debris on the historical nesting grounds of green turtles (*Chelonia mydas*) in Hainan Island, South China. Heliyon. 2023;9: e13400. doi: 10.1016/j.heliyon.2023.e13400.

59. Okuyama J, Watabe A, Takuma S, Tanaka K, Shirai K, Murakami- Sugihara N, et al. Latitudinal cline in the foraging dichotomy of loggerhead sea turtles reveals the importance of East China Sea for priority conservation. Divers Distrib. 2022;28: 1568-1581. doi: 10.1111/ddi.13531.

60. Kameda K, Wakatsuki M, Takase M, Nakanishi Y, Kamezaki N. Apparent survival probability and abundance of juvenile green turtles in the foraging ground at Kuroshima Island, Ryukyu Archipelago. Endang Species Res. 2023;50: 209-215. doi: 10.3354/esr01228.

61. Kikukawa A, Kamezaki N, Ota H. Current status of the sea turtles nesting on Okinawajima and adjacent islands of the central Ryukyus, Japan. Biol Conserv. 1999;87: 149-153. doi: 10.1016/S0006-3207(98)00047-0.

62. Kawazu I, Okabe H, Kobayashi N. Direct observation of mating behavior involving one female and two male loggerhead turtles in the wild. Curr Herpetol. 2017;36: 69-72. doi: 10.5358/hsj.36.69.

63. Kim J, Kim IH, Kim MS, Lee HR, Kim YJ, Park S, et al. Occurrence and diet analysis of sea turtles in Korean shore. J Ecol Environ. 2021;45: 1-15. doi: 10.1186/s41610-021-00206-w.

64. KHOA (Korea Hydrographic and Ocenographic Agency). Analysis of sea surface temperature in the coast of Korean Peninsula using satellite images; 2016. [Cited June 22, 2023]. Available from: http://www.khoa.go.kr/eng/on.

65. Kinoshita C, Fukuoka T, Niizuma Y, Narazaki T, Sato K. High resting metabolic rates with low thermal dependence induce active dives in overwintering Pacific juvenile loggerhead turtles. J Exp Biol. 2018;221: jeb175836. doi: 10.1242/jeb.175836.

66. Pilcher NJ, Antonopoulou MA, Rodriguez-Zarate CJ, Mateos-Molina D, Das HS, Bugla I, et al. Movements of green turtles from foraging areas of the United Arab Emirates: regional habitat connectivity and use of marine protected areas. Mar Biol. 2021;168: 1-15. doi: 10.1007/s00227-020-03815-6.

67. Esteban N, Mortimer JA, Stokes HJ, Laloë JO, Unsworth RK, Hays GC. A global review of green turtle diet: sea surface temperature as a potential driver of omnivory levels. Mar Biol 2020;167: 1-17. Doi: 10.1007/s00227-020-03786-8.

68. Nishizawa H, Abe O, Okuyama J, Kobayashi M, Arai N. Population genetic structure and implications for natal philopatry of nesting green turtles Chelonia mydas in the Yaeyama Islands, Japan. Endang Species Res. 2011;14: 141-148. doi: 10.3354/esr00355.

69. Ng CKY, Dutton PH, Gu HX, Li TH, Ye MB, Xia ZR, et al. Regional conservation implications of green turtle (*Chelonia mydas*) genetic stock composition in China. Chelonian Conserv Biol. 2017;16: 139-150. doi: 10.2744/CCB-1253.1.

70. McClellan CM, Read AJ. Complexity and variation in loggerhead sea turtle life history. Biol Lett. 2007;3: 592-594. doi: 10.1098/rsbl.2007.0355.

71. Overland JE, Wang M. Future climate of the north Pacific Ocean. Eos Trans AGU. 2007;88: 178-182. doi: 10.1029/2007EO160003.

72. Kim S, Woo S, Kim B, Hur S. Trends in sea surface temperature (SST) change near the Korean peninsula for the past 130 years. Ocean Polar Res. 2011;33: 281-290. doi: 10.4217/OPR.2011.33.3.281.

73. Han IS, Lee JS. Change the annual amplitude of sea surface temperature due to climate change in a recent decade around the Korean Peninsula. J Korean Soc Mar Environ Saf. 2020;26: 233-241. doi: 10.7837/kosomes.2020.26.3.233.

74. Finkbeiner EM, Wallace BP, Moore JE, Lewison RL, Crowder LB, Read AJ. Cumulative estimates of sea turtle bycatch and mortality in USA fisheries between 1990 and 2007. Biol Conserv. 2011;144: 2719-2727. doi: 10.1016/j.biocon.2011.07.033.

75. Wallace BP, Kot CY, DiMatteo AD, Lee T, Crowder LB, Lewison RL. Impacts of fisheries bycatch on marine turtle populations worldwide: toward conservation and research priorities. Ecosphere. 2013;4: 1-49. doi: 10.1890/ES12-00388.1.

Table 1. Physical characteristics and tracking details of the tracked loggerhead and green turtles tracked.

ID	CCL (cm)	BW (kg)	Tracking duration (days)	Distance traveled (km)		Home range (1,000 km^2)		
				Total	Daily	MCP	95 PVC	50 PVC
Loggerhead turtle								
KOR0001	74.0	55.0	18/06/2016 - 23/07/2016 (35)	129.8	5.8 ± 3.8	0.4	0.4	0.0
KOR0008	76.0	56.0	28/09/2017 - 24/12/2017 (87)	2,862.2	37.9 ± 37.2	417.7	15.4	1.4
KOR0091	79.9	52.6	29/08/2018 - 13/10/2018 (45)	1,055.2	24.0 ± 14.1	36.2	41.5	5.7
KOR0092	91.3	85.2	17/10/2018 - 11/09/2019 (329)	6,217.0	18.8 ± 15.7	253.9	164.3	30.7
KOR0149	73.2	82.2	26/08/2021 - 12/12/2021 (108)	1,756.5	18.4 ± 11.4	50.4	85.4	15.3
KOR0151	73.0	77.8	26/08/2021 - 09/10/2022 (409)	8,964.8	22.2 ± 14.4	489.9	333.5	23.2
KOR0155	71.0	69.7	25/08/2022 - 27/09/2022 (33)	598.5	18.1 ± 9.3	20.9	30.3	5.5
Mean	76.9 ± 6.9	68.4 ± 13.8	(149.5 ± 154.2)	3,083.4 ± 3,297.3	20.7 ± 9.5	181.3 ± 205.4	95.8 ± 118.4	11.7 ± 11.7
Green turtle								
KOR-1	65.6	31.8	29/10/2015 – 15/12/2015 (47)	1,601.5	32.8 ± 23.0	133.1	30.7	5.3
KOR-2	60.0	22.5	29/10/2015 - 29/10/2016 (366)	1,461.3	6.2 ± 11.9	41.8	15.8	6.0
KOR0003	95.8	103.0	12/08/2016 - 17/10/2016 (66)	1,291.7	25.5 ± 26.0	75.1	25.1	7.1
KOR0004	68.0	41.4	02/09/2016 - 26/10/2016 (54)	1,452.3	26.3 ± 17.4	162.1	145.4	20.7
KOR0009	78.8	58.9	28/09/2017 - 28/09/2018 (365)	6,484.3	18.7 ± 17.3	221.7	140.3	14.3
KOR0104	77.6	69.8	07/10/2018 - 01/05/2019 (206)	5,203.5	27.5 ± 22.1	1,056.8	246.9	4.3
KOR0129	77.3	70.9	28/08/2019 - 26/10/2019 (59)	2,412.4	43.5 ± 33.0	221.2	244.8	34.2
KOR0148	70.4	79.2	26/08/2021 - 26/07/2022 (334)	1,623.0	7.5 ± 12.5	34.7	47.3	6.6
Mean	74.2 ± 10.9	59.7 ± 26.7	(187.2 ± 148.4)	2,691.3 ± 2,004.0	23.5 ± 12.5	243.3 ± 336.8	112.0 ± 96.6	12.3 ± 10.5

CCL = curved carapace length; BW = body weight; MCP = minimum convex polygon; PVC = percentage volume contour.

Table 2. Summary of the six environmental variables measured in this study.

Environmental variable	Resolution	Frequency	Source
Distance to shore (km)			
Bathymetry (m)	~ 1 km		GEBCO (https://www.gebco.net/)
Sea surface temperature (°C)	~ 4 km	8-day mean	NASA OceanColor Web (https://oceancolor.gsfc.nasa.gov)
Sea surface salinity (‰)	~ 40 km	8-day mean	EarthData (https://earthdata.nasa.gov)
Chlorophyll-a (mg/m^3)	~ 4 km	8-day mean	NASA OceanColor Web (https://oceancolor.gsfc.nasa.gov)
Particulate organic carbon (mol/m^3)	~ 4 km	8-day mean	NASA OceanColor Web (https://oceancolor.gsfc.nasa.gov)

Table 3. Mean value ± standard deviation of the six environmental variables measured at the transitional and foraging areas of each sea turtle.

ID	Type of area	Distance to shore (km)	Bathymetry (m)	SST (°C)	SSS (‰)	Chla (mg/m^3)	POC (mol/m^3)
Loggerhead turtle							
KOR0001	T	3.2 ± 3.3	21.2 ± 13.8	22.8 ± 1.2	31.4 ± 1.7	1.76 ± 0.94	230.8 ± 76.4
	F	-	-	-	-	-	-
KOR0008	T	129.4 ± 78.0	1,795.4 ± 2,110.3	26.4 ±1.7	33.5 ± 1.4	0.49 ± 0.7	112.7 ± 165.4
	F	2.0 ± 1.3	13.6 ± 18.3	24.0 ± 1.1	34.8 ±1.0	0.22 ±0.10	77.4 ± 25.0
KOR0091	T	43.8 ± 49.1	85.6 ± 22.3	24.6 ± 1.8	32.5 ± 1.4	0.77 ± 0.37	140.4 ± 41.5
	F	-	-	-	-	-	-
KOR0092	T	58.6 ± 38.3	363.6 ± 549.7	21.4 ± 2.6	33.1 ± 1.4	0.52 ± 0.41	113.1 ± 66.8
	F	83.6 ± 55.5	119.3 ± 30.2	19.0 ± 2.1	34.0 ± 1.3	0.49 ± 0.25	118.0 ± 46.6
KOR0149	T	124.7 ± 74.7	65.1 ± 19.8	21.4 ±3.4	31.2 ± 0.9	1.62 ± 0.70	232.1 ± 78.0
	F	89.1 ± 22.9	79.5 ± 9.7	22.6 ± 2.9	31.5 ± 0.8	1.18 ± 0.63	195.1 ± 79.6
KOR0151	T	32.4 ± 30.1	304.1 ± 502.3	20.0 ± 4.1	33.1 ± 2.1	0.44 ± 0.44	101.6 ± 61.1
	F	42.4 ± 38.1	733.9 ± 557.0	22.4 ± 3.2	32.5 ± 1.6	0.69 ± 0.46	163.1 ± 112.4
KOR0155	T	85.1 ± 67.7	87.8 ± 33.1	26.8 ± 2.2	31.5 ± 1.1	0.64 ± 0.47	122.8 ± 53.2
	F	-	-	-	-	-	-
Mean	T	68.2 ± 47.3	389.1 ± 633.5	23.4 ± 2.7	32.3 ± 0.9	0.89 ± 0.56	150.5 ± 56.5
	F	54.3 ± 40.6	236.6 ± 334.4	22.0 ± 2.1	33.2 ± 1.5	0.64 ± 0.41	138.4 ± 51.5
Green turtle							
KOR-1	T	9.7 ± 13.6	58.4 ± 42.8	21.1 ± 1.4	31.5 ± 3.1	1.60 ± 1.89	167.1 ± 81.8
	F	-	-	-	-	-	-
KOR-2	T	6.4 ± 8.2	71.3 ± 80.9	20.2 ± 1.5	33.0 ± 1.1	0.95 ± 0.88	166.0 ± 103.1
	F	1.5 ± 1.8	23.5 ± 20.1	23.9 ± 3.7	33.6 ± 1.4	0.26 ± 0.11	69.1 ± 22.6
KOR0003	T	9.0 ± 15.9	57.2 ± 47.2	26.9 ± 2.2	31.6 ± 3.1	0.7 ± 0.4	124.9 ± 39.5
	F	1.3 ± 1.2	27.9 ± 17.7	23.8 ± 1.0	31.7 ± 0.4	0.63 ± 0.15	121.9 ± 27.6
KOR0004	T	131.2 ± 93.0	189.7 ± 278.0	26.6 ± 0.9	32.4 ± 1.6	0.58 ± 0.34	104.8 ± 33.7
	F	-	-	-	-	-	-
KOR0009	T	58.0 ± 46.0	465.1 ± 563.8	19.6 ± 3.8	33.6 ± 1.3	0.68 ± 0.81	123.0 ± 97.1
	F	26.9 ± 27.4	84.7 ± 80.9	21.7 ± 4.3	33.8 ± 1.4	0.95 ± 0.74	167.6 ± 94.2
KOR0104	T	32.2 ± 41.4	33.0 ± 27.9	23.5 ± 2.2	32.9 ± 1.6	1.59 ± 1.02	211.4 ± 89.0
	F	-	-	-	-	-	-
KOR0129	T	81.0 ± 67.4	52.1 ± 40.1	25.2 ± 1.5	30.6 ± 2.9	1.84 ± 1.08	230.9 ±91.5
	F	-	-	-	-	-	-
KOR0148	T	17.1 ± 21.0	191.6 ± 207.2	18.5 ± 1.5	32.4 ± 1.6	0.46 ± 0.24	105.3 ± 36.7
	F	1.5 ± 1.5	82.7 ± 156.4	21.8 ± 4.1	33.4 ± 1.6	0.50 ± 0.51	111.0 ± 59.9
Mean	T	43.1 ± 44.5	139.8 ± 145.5	22.7 ± 3.3	32.3 ± 1.0	1.06 ± 0.54	154.2 ± 48.0
	F	7.8 ± 12.7	54.8 ± 33.6	22.8 ± 1.2	33.1 ± 1.0	0.59 ± 0.29	104.6 ± 50.1

SST = sea surface temperature; SSS = sea surface salinity; Chla = Chlorophyll-a; POC = particulate organic carbon; T = transitional area; F = foraging area.

Table 4. Comparison of the six environmental variables within and between species. Interspecific differences were only compared in foraging areas corresponding to both species, while the columns with each species title indicate differences in the variables between the foraging and travel areas using t-test. Asterisk indicates significant differences (p-value < 0.05).

Variables	Interspecific foraging use	Loggerhead turtle	Green turtles
DDT	t = 15.314<br>p < 0.001*	t = 6.997<br>p < 0.01*	t = 21.294<br>p < 0.001*
DTS	t = 24.622<br>p < 0.001*	t = -2.104<br>p < 0.05*	t = 16.249<br>p < 0.001*
Bathy	t = -14.767<br>p < 0.001*	t = -0.587<br>p > 0.05	t = -8.840<br>p < 0.001*
SST	t = -6.281<br>p < 0.001*	t = 2.878<br>p < 0.01*	t = -1.516<br>p > 0.05
SSS	t = -4.208<br>p < 0.001*	t = -2.718<br>p < 0.01*	t = -8.013<br>p < 0.001*
Chla	t = 3.300<br>p = 0.001*	t = -0.210<br>p > 0.05	t = 11.307<br>p < 0.001*
POC	t = 6.411<br>p < 0.001*	t = - 3.479<br>p < 0.01*	t = 9.751<br>p < 0.001*

Fig 1. Summarized travel patterns of the sea turtles released at the coastal seas of South Korea. White circles and squares with each color indicate start and end points, respectively. This map was generated based on GADM data (https://dadm.org/).

Fig 2. Travel patterns and core habitat of the seven satellite-tracked loggerhead turtles. Green zones indicate foraging areas calculated by 50 PVC. Dotted line areas indicate 50 PVC zones that were excluded from the core habitat. Dates marked with asterisks correspond to the year following the year of release. The red and blue arrows indicate the start and end dates of the tracking period, respectively. **This map was generated based on GADM data (https://dadm.org/).**

Fig 3. Travel patterns and core habitat of the eight satellite-tracked green turtles. Green and red zones indicate foraging and nesting areas calculated by 50 PVC, respectively. Dotted line areas indicate 50 PVC zones that were excluded from the core habitat. Dates marked with asterisks correspond to the year following the year of release. The red and blue arrows indicate the start and end dates of the tracking period, respectively. **This map was generated based on GADM data (https://dadm.org/).**

Fig 4. Monthly changes in latitude, daily distance traveled, and six environmental variables (mean ± standard error) measured in the foraging areas of the both sea turtle species.

Fig 5. Travel routes and environmental variables on the travel route of two representative loggerhead turtles. Green zones on the maps and the dotted sections in the graphs indicate core areas by 50 PVC. POC = particulate organic carbon. **This map was generated based on GADM data (https://dadm.org/).**

Fig 6. Travel routes and environmental variables on the travel route of two representative green turtles. Green and pink zones on the maps and the dotted sections in the graphs indicate core areas by 50 PVC. POC = particulate organic carbon. **This map was generated based on GADM data (https://dadm.org/).**

Click here to access/download;Figure;Fig3.docx

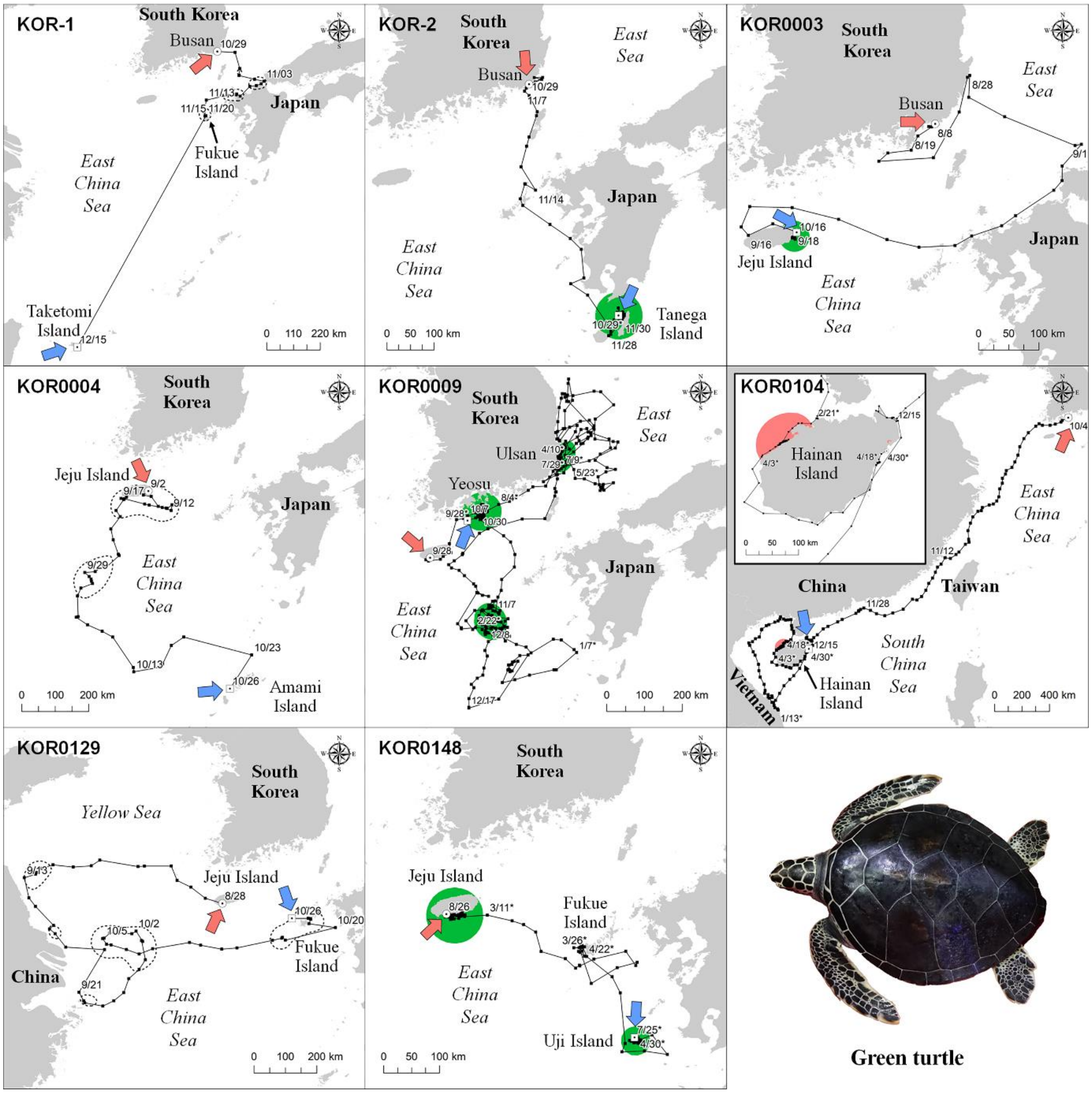

Click here to access/download;Figure;Fig4.docx

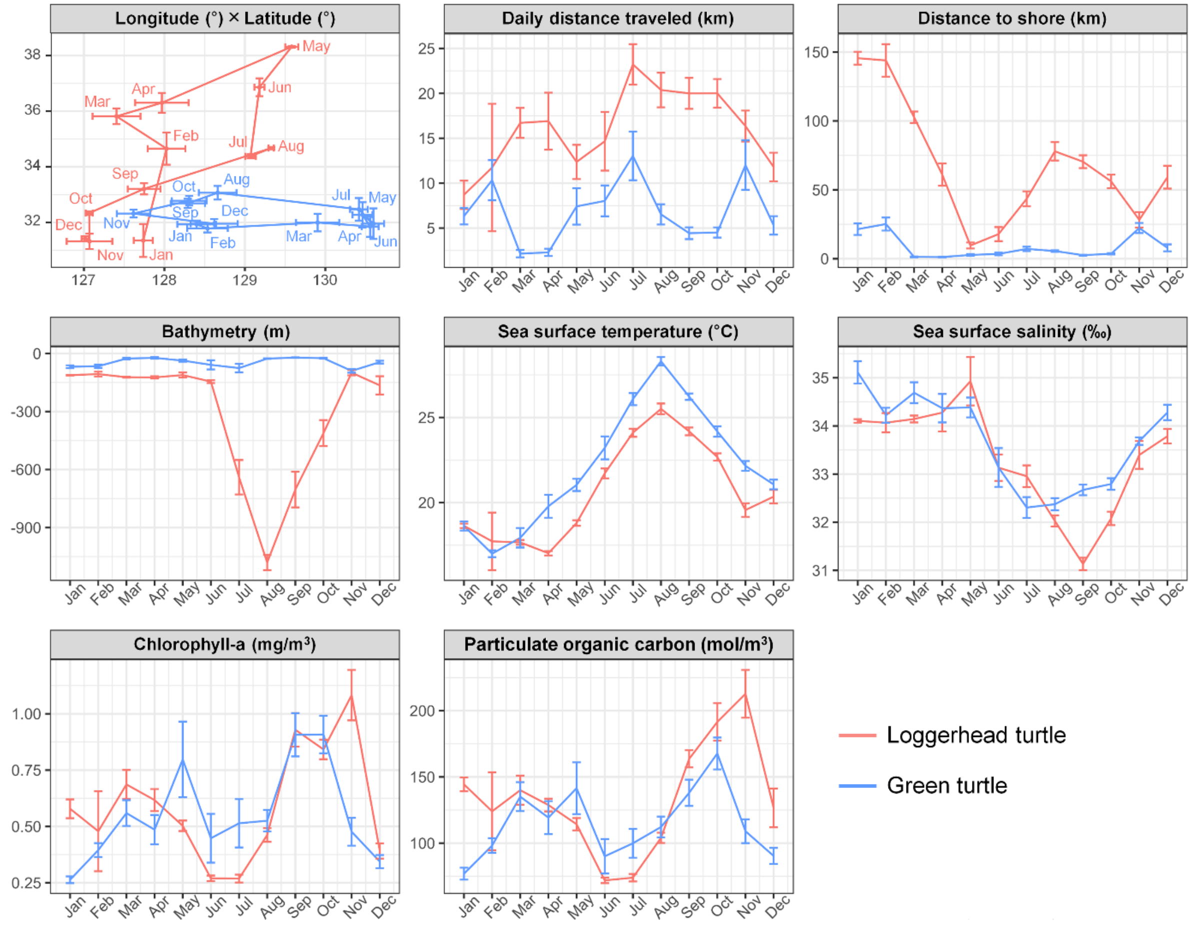

Click here to access/download;Figure;Fig5.docx

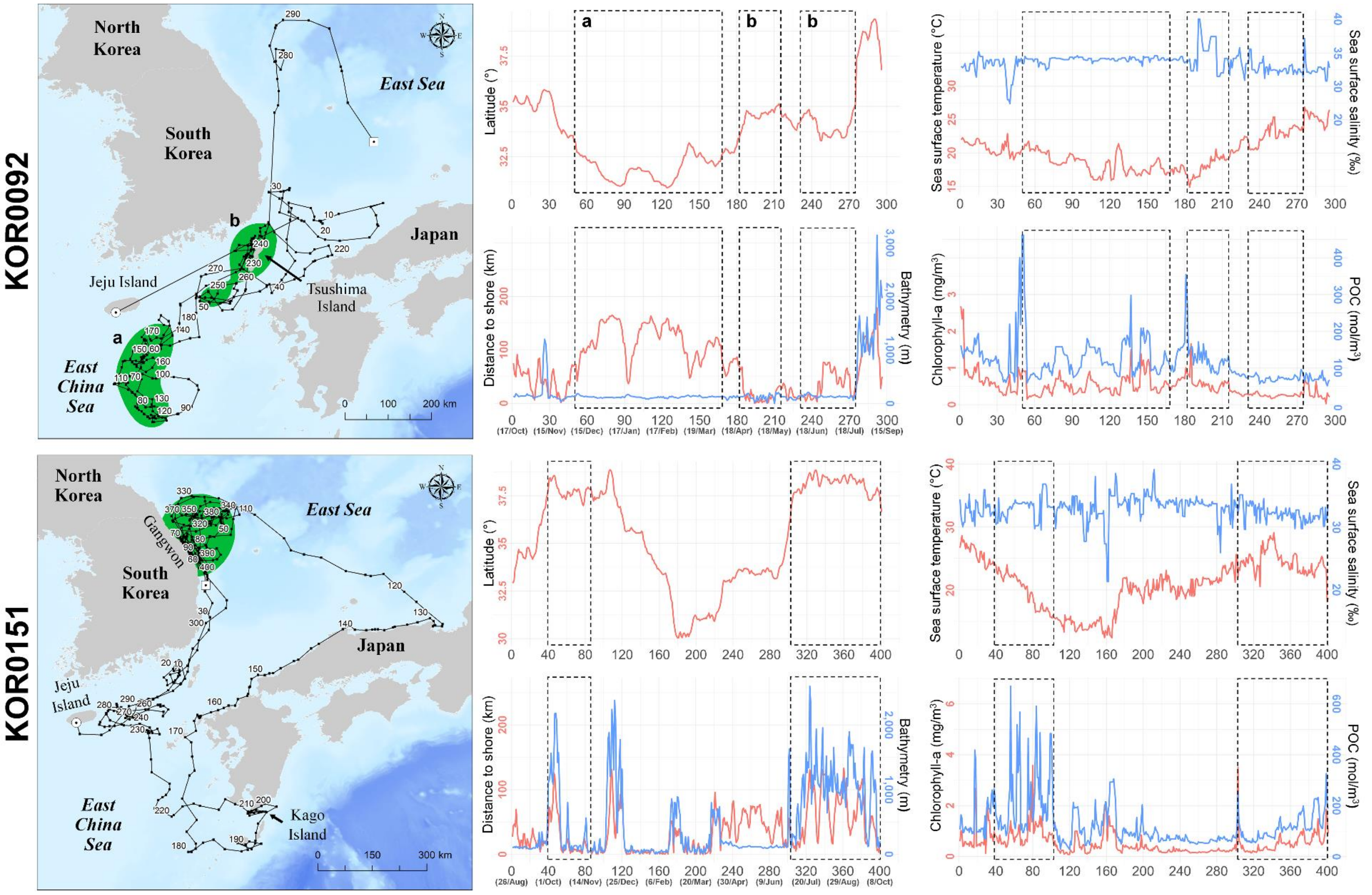

Click here to access/download;Figure;Fig6.docx

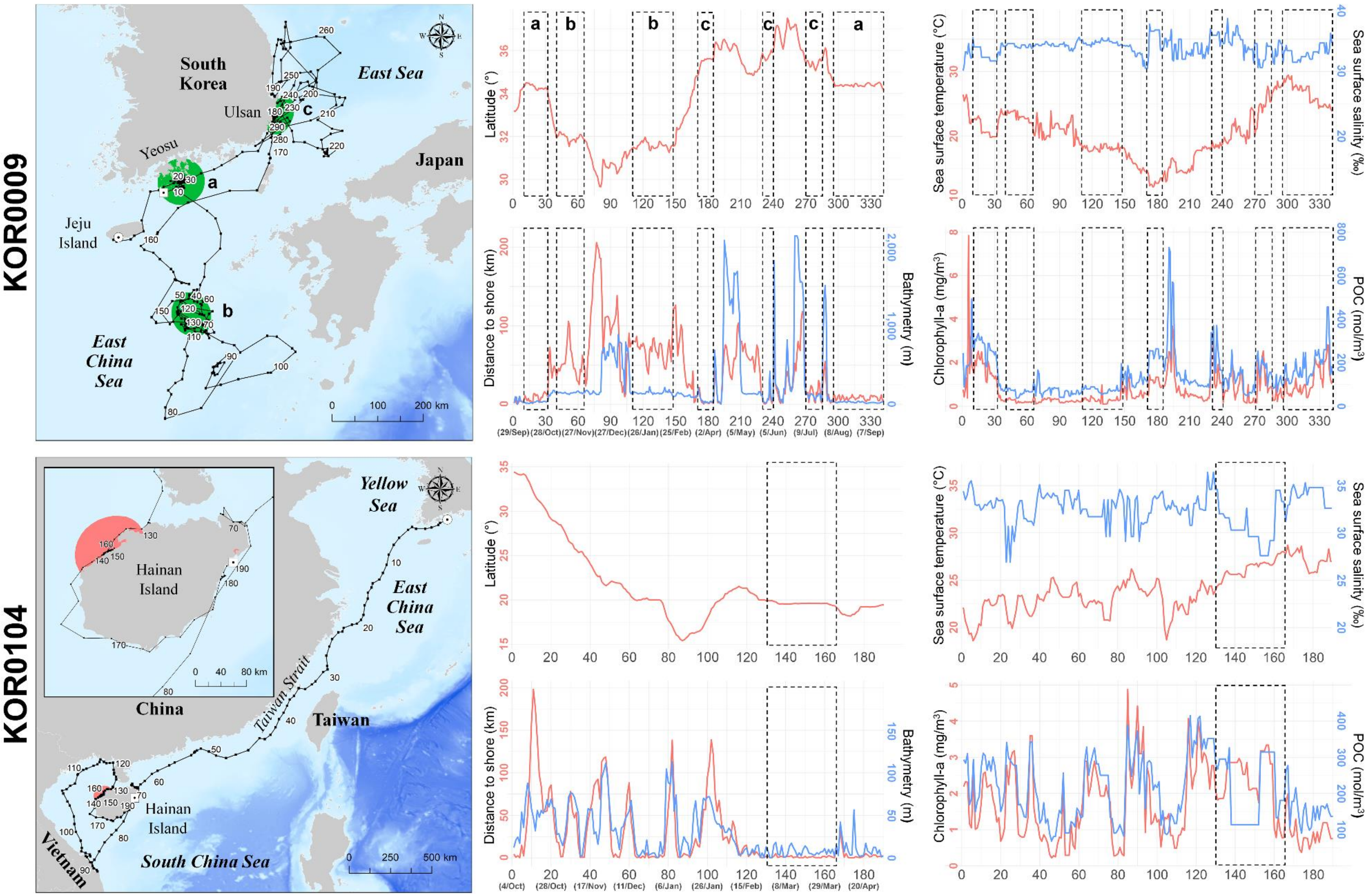

Figure Click here to access/download;Figure;Fig1_revised.docx

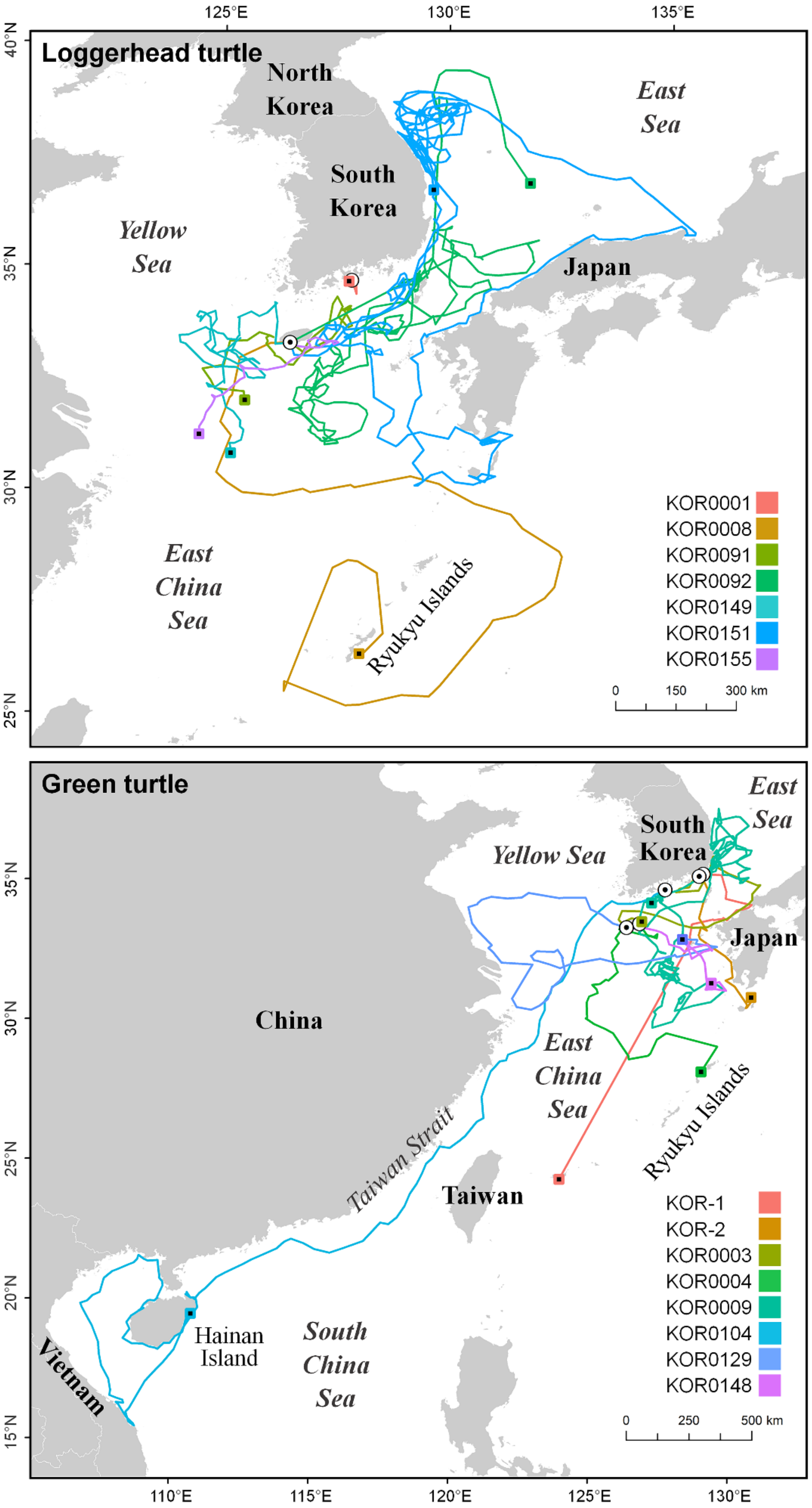

Figure
Click here to access/download;Figure;Fig2_revised.docx

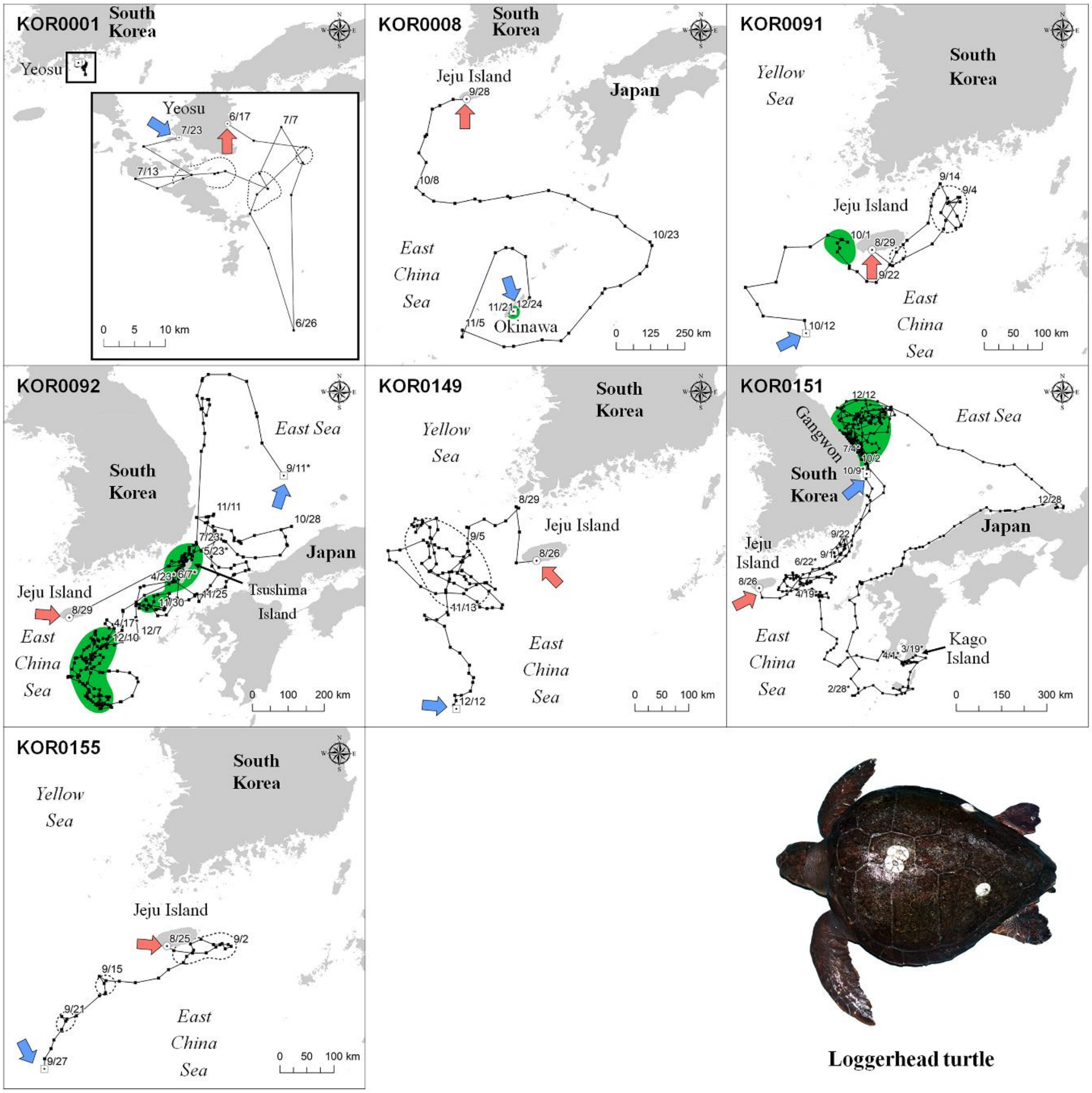

Click here to access/download
Supporting Information
Supporting Information.docx

Click here to access/download
Supporting Information
Comments on animal research ethics.doc

Click here to access/download

Supporting Information

official paper_animal research ethics(2018).pdf

Click here to access/download

Supporting Information - Compressed/ZIP File Archive

2017-2022_Marine Animal Protection Committee.zip

Click here to access/download

Supporting Information - Compressed/ZIP File Archive

sea turtle movebank file.zip

Habitat use of loggerhead (*Caretta caretta*) and green (*Chelonia mydas*) turtles [illegible] the northern limit of their distribution range of the Northwest Pacific Ocean~~, the northern limit of their distribution range~~

Short title: Habitat use of loggerhead and green turtles in the Northwest Pacific Ocean

Il-Hun Kim[1¶], Il-Kook Park[2¶], Daesik Park[2], Min-Seop Kim[1], In-Young Cho[1], Dongwoo Yang[1], Dong-Jin Han[3], Eunvit Cho[3], Won Joon Shim[4], Sang Hee Hong[4,5], Yong-Rock An[1*]

[1] Department of Ecology and Conservation, National Marine Biodiversity Institute of Korea, Seocheon, Chungcheongnam-do, Republic of Korea

[2] Division of Science Education, Kangwon National University, Chuncheon, Gangwon-do, Republic of Korea

[3] Aqua Team, Aqua Planet Yeosu, Yeosu, Jeollanam-do, Republic of Korea

[4] Ecological Risk Research Department, Korea Institute of Ocean Science and Technology, Geoje, Gyeongsangnam-do, Republic of Korea

[5] Department of Ocean Science, University of Science and Technology, Daejeon, Republic of Korea

*Corresponding author

E-mail: rock@mabik.re.kr (Y-RA)

[¶]These authors contributed equally to this work.

Abstract

Verifying habitats, including the foraging and nesting areas for sea turtles, enables an understanding of their spatial ecology and successful planning of their conservation and management strategies. Recently, the observation frequency and bycatch of loggerhead (*Caretta caretta*) and green (*Chelonia mydas*) turtles have increased in the northern limit of their distribution range, in the northern part of the East China Sea and East (Japan) Sea. We conducted satellite tracking to investigate the habitat use of seven loggerhead and eight green turtles from June 2016 to August 2022 in this area, where little is known about their spatial ecology. We applied a 50 percent volume contour method to determine their main foraging areas and analyzed 6 environmental variables to characterize their habitats. Loggerhead turtles mainly stayed in and used the East China Sea as a foraging area during the tracking period, while two individuals among them also used the East Sea as a seasonal foraging area. Most green turtles also used the East China Sea as a foraging area, near South Korea and Japan, with one individual among them using the lower area of the East Sea as a seasonal foraging area. Notably, one green turtle traveled to Hainan Island in the South China Sea, a historical nesting area. Our results showed that the two sea turtle species included the East Sea as a seasonal foraging area, possibly owing to the abundance of food sources available, despite its relatively lower sea temperature. Considering that loggerhead and green sea turtles were observed using the northern part of the East China Sea and East Sea more frequently than previously known and that the sea temperature gradually increases due to climate change, conservation and management activities are required for sea turtles in these areas.

Introduction

Although many regional sea turtle populations are increasing thanks to conservation efforts [1,2], still six out of All seven known sea turtle species are threatenedendangered worldwide, which has promoted conservation and restoration efforts from many countries and global organizations, such as the Convention on International Trade in Endangered Species of Wild Fauna and Flora (CITES) and the International Union for Conservation of Nature (IUCN), are trying to conserve and restore them. Understanding the spatial and foraging ecology of sea turtles is essential for establishing successful conservation and management strategies [2-5]. However, becausesince sea turtles have a wide distribution range, diverse habitat types, and a complex life cycle, it is necessary to invest considerable time and effort to thoroughly understand their ecology [6-8]. Currently, considering that the distribution range of marine creatures, including sea turtles, is expanding polewardnorth owing to climate change [9-12], research on the northern edge of the sea turtle range is urgently needed. but currently insufficient. Satellite telemetry is a useful method for investigating the spatial ecology and habitat use of sea turtles, applied in various regions and species [6,13-16]. Although various molecular ecology techniques have been recently used to study population genetic diversity and determine the studying origin of sea turtles [17-21], satellite telemetry remains a powerful study method, particularly for the distribution boundary of sea turtles, which have very low population densities [5,14,16,22]. Because sea turtles show seasonal differences in habitat use, such as for foraging and nesting, and high habitat fidelity [23-26], satellite telemetry can effectively identify the major areas of these habitats [14,27,28]. Furthermore, analyzing these environmental characteristics of the identified habitats could reveal the factors affecting the habitat preferences of sea turtles, leading to proper conservation and management strategies against climate change and anthropogenic developments [29,30]. Overall, the habitat information and environmental characteristics identified by satellite telemetry could contribute to the effective protection and restoration of sea turtle populations [31].

In the Northwest Pacific Ocean, Loggerhead (*Caretta caretta*) and green (*Chelonia mydas*) turtles are the most commonly observed sea turtles and use it as foraging and nesting regionsFive sea turtle species are distributed in the Northwest Pacific Ocean: the loggerhead turtle (*Caretta caretta*), green turtle (*Chelonia mydas*), hawksbill turtle (*Eretmochelys imbricata*), olive ridley sea turtle (*Lepidochelys olivacea*), and leatherback sea turtle (*Dermochelys coriacea*) [32-34]. Sea turtle research in the Northwest Pacific Ocean has focused on the southern coastline of Japan and the southern Ryukyu Islands, where the main foraging and

nesting sites of loggerhead and green turtles are located [19,35-37]. Recently, the nesting frequencies of loggerhead and green turtles are declining as a consequence of coastal development and other unknown factors in these areas [18,24,25]. Nevertheless~~On the other hand~~, the observation frequency of loggerhead and green turtles has increased in their northern distribution boundary region, including the northern parts of the East China Sea and East (Japan) Sea, near the coastline of the Korean Peninsula [34,38,39]. Despite the increased observation of loggerhead and green turtles~~sea turtles~~ at these distribution margins, studies on their ~~sea turtle~~ habitat use are lacking. Several studies have shown that juvenile loggerhead turtles use the East Sea near South Korea and Japan as a route to the open Pacific Ocean, but few studies have been conducted on adult specimens [5,16,40]. In green turtles, some research showed that adults use the sea near Jeju Island [41], whereas juveniles use the Yellow Sea west of the Korean Peninsula [16]. However, the detailed patterns of habitat use by adults have not been studied yet.

In the present study, we aimed to reveal the travel patterns, habitat use, and environmental characteristics of the habitats of subadult and adult loggerhead and green turtles using satellite telemetry. Considering that the habitat use patterns of the two sea turtle species at the northern boundary of their distribution range are not well known, this study is expected to provide essential information about their spatial biology, which can be useful for the effective management and conservation of both sea turtle species in the Northwest Pacific Ocean.

Materials and methods

Satellite telemetry

From October 2015 to October 2022, we tracked 15 rehabilitated sea turtles (7 loggerhead and 8 green turtles), which were caught incidentally and rescued from fishing nets near the coastal area of the Republic of Korea (Table 1; S1 Table). Permissions for the sea turtle conservation program were given by the Marine Animal Protection Committee of Ministry of Oceans and Fisheries with whom this work was conducted. We measured the curved carapace length (CCL) and body weight (BW) of each sea turtle. Considering the minimum CCL of sexually mature sea turtles is known as 73 cm in loggerhead turtles and 67 cm in green turtles [42,43], 12 turtles were identified as adults, and three turtles as ~~except for three~~ potential subadults (KOR-1, KOR-2, and KOR0155). Other than KOR0149, a male loggerhead turtle, the remaining 14 turtles were all female. The turtles were released

between June and October at the coasts of Jeju Island (N 33.2449°, E 126.4128°), Yeosu (N 34.6306°, E 127.7935°), and Busan (N 35.1587°, E 129.1608°) in the Republic of Korea, where the threat of collision with ships was relatively low and the water temperature was relatively warm (16 - 28°C in SST).

To track sea turtles, we used the Argos satellite transmitters (Wildlife Computers Inc., Redmond, WA, USA), SPOT-352A (before September 2019, lifespan: approximately 920 days; 72 × 56 × 22 mm; 129 g), and SPOT-375A (after August 2021, lifespan: 915 days; 99 × 55 × 21 mm; 152 g). The Argos transmitter has been widely used for satellite tracking of sea turtles until recently [15, 16, 22, 44], although it could overestimate space use of sea turtles compared to more accurate approaches such as Fastloc-GPS [45,46]. We attached transmitters to the carapaces of each sea turtle using Aqua Mend Epoxy (PSI-Polymer Systems, NC, USA) and instant mix epoxy (Loctite, CT, USA). Before attaching transmitters, we cleaned the carapace using abrasive sandpaper to remove debris, wiped the carapace with isopropyl alcohol to remove greases [47,48]. To confirm the attachment stability of the transmitter to sea turtles, individuals were monitored for 24 h in an aquarium before release. The tracking period was defined as the period from the first tracking day after the release date to the last day of if the transmitter signal is not observed for two months from the date of the last signal received. Tracking data were downloaded from the ARGOS system (CLS America, http://www.clsamerica.com). The accuracy estimation of the ARGOS system was classified into six categories: class 0, with an error radius > 1,500 m; class 1, with > 1,000 m; class 2, with > 500 m; class 3, with > 150 m; and classes A and B, with no estimated accuracy. We only used the higher-accuracy data for a given day among the recorded daily points, excluding classes A and B. We excluded location data on land without a distinct signal from the transmitter or long-distance travel of over 100 km in a single day, which is impossible for sea turtles to travel [15,16]. Although LC 0, which has lower location quality, could overestimate home range size, the reduction in the number of location points due to the exclusion of LC 0 obscures the understanding the space use patterns of sea turtles [46]. Because the aim of this study was to focus on space use patterns in little-known areas rather than determining the detailed home range size of sea turtles, we included LC 0 for analysis. Additionally, reassessment of location accuracy is necessary considering studies showing that LC A has higher accuracy than LC 0 [45,49].

Travel pattern and home range

We measured the distance that sea turtles traveled as the shortest straight line between two consecutively tracked location points in order to study their travel habits. The daily distance traveled was calculated as the distance between the given point and the point on the previous day at the most similar time as possible. In addition, the total distance traveled by each turtle was calculated as the sum of the total distances traveled during the entire tracking period.

To define the home range size of individual sea turtles during the tracking periods, we used the minimum convex polygon (MCP) and 95 and 50 percentage volume contours (PVC) based on kernel density estimation, often used to calculate the home range of sea turtles [14,16,41,50]. The MCP and 95 PVC represent general home range sizes, while the 50 PVC indicates the core area of each individual. The distances traveled and home ranges were calculated, after which tracked turtle travel maps were generated using ArcGIS v. 10.1 (ESRI, Redlands, California, USA).

Habitat use

We compared the DDT of location data inside and outside 50 PVC, and defined the 50 PVC as a foraging area if the DDT inside was significantly smaller, based on the slow travel speed of sea turtles in their foraging areas [51,52]. On the other hand, we defined all areas outside 50 PVC and where DDT was not significantly small as transitional areas. ~~defined the 50 PVC as the area where turtles stayed for more than 15 days while foraging.~~ We also defined 50 PVC nesting areas for adult turtles, that stayed near areas where nesting was previously recorded during their known breeding period. We identified all location points as either foraging, nesting, or transitional~~veling~~ areas.

To understand the environmental characteristics of the habitat used by sea turtles, we collected and analyzed data regarding six environmental variables at each location, that are often used for sea turtle studies [14,50,53]: distance to shoreline (DTS, km), bathymetry (m), sea surface temperature (SST, °C), sea surface salinity (SSS, ‰), chlorophyll-a (Chla, mg/m^3), and particulate organic carbon (POC, mol/m^3) (Table 2). Distance to shore and bathymetry represent the topographical characteristics of the location where turtles were present, whereas SST and SSS are related to sea surface conditions at each location. Chlorophyll-a and POC, as indicators of the food source such as seagrass, are related to food abundance [29,54]. Also, we presented the whole tracking data and

following environmental variable changes together on the four longest-tracked sea turtles (two loggerhead and two green turtles) and on the one farthest-traveled green turtle to show the relationship between environmental variables along the travel and habitat use of sea turtles. The six environmental variables were extracted using SeaDAS v. 8.3.0 (https://seadas.gsfc.nasa.gov/) and ArcGIS v. 10.1 (ESRI, Redlands, California, USA). Graphs were created using the ggplot2 package [55] in R v. 4.0.5 software [56].

Statistical analysis

To distinguish between the foraging or nesting area and the transitional area, we used an independent t-test, a one-way ANOVA test, and the Kruskal-Wallis test. First, we conducted the Shapiro-Wilkinson test with DDT value of each sea turtle for normality. When only one 50 PVC was generated in a sea turtle individual, the inside and outside DDT were compared using a t-test. If a sea turtle has multiple 50 PVCs, we conducted one-way ANOVA or Kruskal-Wallis test on the inside and outside of each 50 PVC. Whenever significant differences were detected, we defined only 50 PVC that had significantly small DDT compared to the outside as the foraging or nesting area by the Bonferroni or Dunn post hoc test.

Based on the environmental variables obtained for the 50 PVC areas, we identified the preferred foraging environment characteristics of both sea turtle species. Additionally, we used an independent sample t-test to compare the interspecific differences in the variables between different foraging areas and the differences in variables between the foraging and transitional areas~~ and the travel~~ of loggerhead and green turtles. We considered it statistically significant when the p-value was under 0.05. Statistical analyses were conducted using SPSS v. 23.0 (IBM Corp., Armonk, NY, USA).

Results

Satellite telemetry

During the tracking period, 7 loggerhead turtles (6 adults and 1 subadult), with a mean CCL of 76.9 ± 6.9 cm and

BW of 52.6 ± 13.8 kg, were tracked for 149.5 ± 154.2 days, traveling between 25.1° N, 123.9° E to 39.3° N, 135.5° E (Table 1). The mean daily distance traveled by loggerhead turtles was 20.7 ± 9.5 km, while the total distance traveled was 3,083.4 ± 3,297.3 km (range: 129.8 – 8,964.8). The mean home range size of loggerhead turtles was 181,342 ± 205,355 km^2 by MCP and 838 ± 118,402 km^2 by 95 PVC, while the core habitat area was 11,703 ± 11,725 km^2 by 50 PVC. The mean values of the six environmental variables in the foraging areas of the loggerhead turtles were 54.3 km of DTS, 236.6 m of bathymetry, 22.0°C of SST, 33.2‰ of SSS, 0.64 mg/m^3 of Chla, and 138.4 mol/m^3 of POC (Table 3).

During the tracking duration, 8 green turtles (6 adults and 2 subadults), with a mean CCL of 74.2 ± 10.9 cm and BW of 59.7 ± 26.7 kg, were tracked for 187.2 ± 148.4 days, with movements ranging from 15.4° N, 106.9° E to 37.5° N, 131.2° E (Table 1). The mean daily distance traveled by green sea turtles was 23.5 ± 12.5 km, while the total distance traveled was 2,691.3 ± 2,004.0 km (range: 1,291.7 – 6,484.3). The mean home range size of green turtles was 243,329 ± 336,756 km^2 by MCP and 112,031 ± 96,626 km^2 by 95 PVC, and the core habitat area was 12,323 ± 10,466 km^2 by 50 PVC. The mean values of the environmental variables in the foraging areas of green turtles were 7.8 km of DTS, 54.8 m of bathymetry, 22.8°C of SST, 33.1‰ of SSS, 0.59 mg/m^3 of Chla, and 104.6 mol/m^3 of POC (Table 3).

Seasonal travel pattern

Seven loggerhead turtles were released in South Korea at 33.2° N – 34.6° N during summer and autumn, from June to September (Table 1). Five loggerhead turtles traveled southward from the release location and stayed below 35° N throughout the tracking period, whereas KOR0092 and KOR0151 traveled up to the East Sea at 39°N during the summer and autumn, between July and early December (Fig 1). Eight green turtles were released in South Korea at 33.2° N – 35.2° N during summer and autumn, from August to October (Table 1). Similar to the loggerhead turtles, seven green turtles moved southward from the release location and stayed below 35° N. Only KOR0009 traveled to the East Sea up to 37.5° N during the spring and summer, between April and July (Fig 1).

Habitat use and environmental variables

Loggerhead turtles used areas in the sea near Okinawa Island (KOR0008), Tsushima Islands (KOR0092), the East China Sea close to Jeju Island (KOR0091 and KOR0092~~ and KOR0149~~), and the east coastal sea of the Gangwon Province in South Korea (KOR0151) for foraging (Fig 2; S2 Table). No loggerhead turtles were presumed to have used the nesting areas during the tracking period because the areas they used had no previous nesting records. Okinawa Island, which is the only known nesting area, was only used during the non-breeding season. In foraging areas, loggerhead turtles traveled shorter distance per day (p-value < 0.05) and stayed closer to the shore than during transitional~~vel~~ periods (p-value < 0.001). In addition, the foraging sites of loggerhead turtles had lower SST (p-value < 0.05), higher SSS (p-value < 0.05), and lower POC (p-value < 0.05) values than of transitional~~veling~~ areas (Table 3).

The major foraging areas for four green turtles were identifi[illegible] KOR-2 used the sea close to Tanega Island, while KOR0003 used the sea close to Jeju Island. KOR0148 used the sea near Jeju Island in South Korea and Uji Island, and KOR0009 used three foraging areas in the sea near Yeosu and Ulsan, and the East China Sea, west of Kyushu in Japan (Fig 3; S2 Table). Furthermore, the nesting areas of [illegible]R0104 were located near Hainan Island, a historical nesting site for green turtles [57,58]. The daily distance traveled (p-value < 0.05) and DTS (p-value <0.05) of green turtles were larger in their transitional~~veling~~ areas than those in their foraging areas (Table 3). Considering the bathymetry values, green turtles used shallower areas for foraging than that used for travel (p-value < 0.05). There was no significant difference in SST between the transitional~~vel~~ and foraging areas (p-value < 0.05), whereas SSS was higher in transitional~~vel~~ areas than that in foraging areas (p-value < 0.05). Both Chla (p-value < 0.05) and POC (p-value < 0.05) were higher during travel than during foraging.

In the foraging areas used by loggerhead (n = 408) and green turtles (n = 545), the observed variables corresponding to loggerhead turtles showed a longer average daily distance traveled (p-value < 0.05) and larger DTS (p-value < 0.05), deeper bathymetry (p-value < 0.05), lower SST (p-value < 0.05), lower SSS (p-value < 0.05), and higher Chla (p-value < 0.05) and POC (p-value < 0.05; Table 4) than those corresponding to green turtles (Table 3). Also, we represented the monthly changes in foraging areas of the six variables for both sea turtles (Fig. 4).

Representative habitat use~~travel~~ pattern

KOR0092 (329 days) and KOR0151 (401 days), which were the longest-tracked loggerhead turtles, gave us the

most detailed information about the environment in the foraging area (Fig. 5). Loggerhead turtle KOR0092 first stayed in the sea south of Jeju Island, between December 10, 2018 and April 17, 2019. After release, KOR0092 moved to and stayed at the sea near Tsushima Island, from April 23, 2019 to May 23, 2019, and from June 7, 2019 to July 22, 2019. Its first major foraging area was located in the open East China Sea, more than 100 km away from land during winter, and with an average of 0.54 mg/m^3 of Chla and 129.2 mol/m^3 of POC. The second major foraging area of KOR0092 was located near Tsushima Island, mainly during the spring and summer (Fig 5). ~~KOR0149 used the sea near the east coast of Gangwon Province in South Korea as a foraging area from October 2, 2021, to December 12, 2021, and from later from July 4, 2022, to October 9, 2022.~~ Immediately after its release, KOR0151 moved to the coast sea east of the Gangwon Province between autumn and early winter. At the start of winter, KOR0151 traveled southward toward Kago Island and used the sea in southern Japan until the end of winter. KOR0151 stayed near the eastern sea of Jeju Island for two months in spring, after which it returned to the eastern coast sea of the Gangwon Province and used it for three months from summer until the end of tracking in autumn.

Compared to loggerhead turtles, green turtles showed wider travel ranges in the sea around South Korea, Japan, China, and Vietnam (Fig 1). Among the eight green turtles, KOR0009 was tracked for the longest period (365 days) and showed three seasonal habitats. Green turtle KOR0009 used three core areas: the coastal sea of Yeosu in South Korea, in autumn (from October 7, 2017 to October 30, 2017 and later from August 4, 2018 to September 28, 2018), the East China Sea in winter (from November 7, 2017 to December 8, 2017 and later from January 15, 2018 to February 22, 2018), and the coastal sea near Ulsan, Gyeongnam Province in South Korea, in spring and summer (from March 24, 2018 to April 10, 2018, from May 24, 2018 to June 5, 2018, and from July 9, 2018 to July 25, 2018). After its release at Jeju Island, KOR0009 used the coastal sea near Yeosu in autumn and traveled to the East China Sea, located at the lowest latitude for overwintering and where the Chla (0.28 mg/m^3) and POC (76.9 mol/m^3) were the lowest. After winter, KOR0009 traveled to the coastal sea of Ulsan, which had 1.26 mg/m^3 of Chla and 217.8 mol/m^3 of POC, where it stayed between spring and summer. Finally, KOR0009 returned to the coastal sea of Yeosu in autumn, where it stayed until the end of the tracking.

KOR0104 was tracked for 206 days and showed a distinct travel pattern reaching Vietnam and Hainan Island, which represents the farthest distance traveled during this study (Fig 6). Upon release in Yeosu, KOR0104 continuously traveled to the South Central Coast of Vietnam for 100 days until January 13, 2019, after which it traveled backward and arrived at Hainan Island, China. The core habitat of KOR0104 was located near the

northwest coast of Hainan Island, where it stayed from February 26, 2019, to April 3, 2019. Its core habitat had 7.2 m of bathymetry, 26°C of SST, 2.30 mg/m^3 of Chla, and 202.0 mol/m^3 of POC, and an average distance from the shoreline of 1.2 km (Fig 6).

Discussion

Habitat use of loggerhead turtle

In this study, loggerhead turtles used the open East China Sea, the coastal sea adjacent to the southern Korean Peninsula and western Japan, and the East (Japan) Sea as their main foraging areas, including the coastal seas of Okinawa Island, Jeju Island, Tsushima Island, and Gangwon Province. Japan has the largest nesting area for loggerhead turtles in the North Pacific Ocean. Major foraging areas adult turtles are located in the seas around Japan, including the East China Sea, the sea west of the Ryukyu Islands, the open North Pacific Ocean, and the southern coastal area of the Japanese Archipelago [24,59]. However, in the present study, the seven tracked loggerhead turtles mainly used the northern part of the East China Sea as foraging areas, which have higher latitudes than those of previously reported areas, such as those near Jeju Island, Tsushima Island, and the east coast of South Korea. These results indicate that the foraging range of loggerhead turtles in the western Northwest Pacific Ocean is wider than previously reported.

All seven loggerhead turtles mainly used the northern part of the East China Sea, which is a suitable habitat for the species owing to being shallow, with a sea depth of less than 100 m and having an annual SST over 20 °C [5,60]. Additionally, in this region, small islands such as Jeju Island, Tsushima Island, and Okinawa Island provide core foraging areas for loggerhead turtles [5,22,61,62]. Furthermore, despite the few records of loggerhead turtles in the East Sea, KOR0092 and KOR0151 periodically used the East Sea in this study. These two turtles showed a foraging loop behavior, using the East Sea in summer and autumn when the SST was higher and the southern East China Sea in winter when the SST was lower. KOR0092 and KOR0151 traveled south in late November and early December and back to the East Sea in the summer. Despite the East Sea having a depth of less than 1,000 m and a relatively low SST, its Chla and POC contents were high, possibly because of an abundance of food sources. Considering such environmental conditions, KOR0092 and KOR0151 might consume enough food in the East Sea during the warm summer and early autumn seasons and then travel to the southern East China Sea for overwintering in the cold winter when the SST drops. Loggerhead turtles use the East Sea near the eastern shoreline of South Korea to forage various seaweeds, jellyfish, and shellfish [63]. In a previous report, over 90% of the loggerhead turtles studied near the Korean Peninsula, with a higher latitude and a lower SST than those of southern Japan, were observed between summer and autumn, between June and November [34]. The main reason

for the observed pattern is thought to be related to low sea temperatures. The coastal sea of South Korea has an SST below 15 °C from December to May [64]; as loggerhead turtles are vulnerable to this sea temperature, they are rarely observed [22,36,65]. Currently, with the sea temperature of the Pacific Ocean continuously rising largely due to climate change, the amount of food sources in the traditional foraging areas of loggerhead turtles is decreasing, forcing them to exploit cooler foraging areas that have more available food sources [9]. As sea temperatures in the Northwest Pacific Ocean increase further, more loggerhead turtles are expected to stay longer in the East Sea and use it as a foraging area.

Habitat use of green turtles

Except for KOR0104, which traveled to Hainan Island, green turtles mainly used the East China Sea between South Korea and Japan, the coastal sea of the Korean Peninsula, and small islands in southern Japan as foraging areas. The major foraging areas of green turtles in the Northwest Pacific Ocean are the coastal sea of southern Japan and the Ryukyu Islands [37]; however, in this study, among the eight turtles, only KOR-1 and KOR0004 traveled to the Ryukyu Islands. The remaining five green turtles, except for KOR0104, used the northern East China Sea as their major foraging area. Tanega Island is a traditional foraging or nesting area for green turtles [60], which was used in the present study by the subadult KOR-2 as an immature foraging area. Jeju Island, which is also a major foraging area for green turtles [34,41], was used by two green turtles, KOR0003 and KOR0148, as their major foraging area in this study. Moreover, they seemed to also use it as an overwintering area considering they stayed there in autumn and winter. Comparable to our results, five of eight green turtles studied by Jang et al. (2018) mainly stayed near Jeju Island during the tracking period, including during winter [41]. These results suggest that green turtles use the upper East China Sea, including the seas near Jeju Island and the southern Korean Peninsula, as both foraging and overwintering areas.

Adult green turtles can have high habitat fidelity for more than one foraging area [4,26,66]. In particular, KOR0009 showed typical foraging loop behavior in three seasonal habitats. Immediately after release, KOR0009 stayed in the sea neighboring of Yeosu in South Korea, in autumn, used the East China Sea at the lowest latitude during winter, used the sea around Ulsan, Gyeongnam Province in South Korea, at the highest latitude during summer, and returned to Yeosu in autumn. Rather than the loggerhead turtles, green turtles have also been reported to use the East Sea near the eastern shoreline of the Korean Peninsula mainly to forage seaweeds [63]. Among the

three habitats, food-related Chla and POC were higher in the sea near Yeosu and Ulsan at high latitudes and relatively low in the East China Sea. Therefore, like loggerhead turtles KOR0092 and KOR0151, green turtle KOR0009 also maintained in the coastal area of South Korea, which provides abundant food sources despite its relatively low sea temperatures, and traveled to the southern East China Sea before winter, when its thermoregulation is limited. Additionally, green turtles consume animal matter more than seagrass at high latitudes or low SST areas under 25°C [67]. However, although green turtles generally used areas below SST of 23°C in our study, the most abundant food item was seaweed according to previous research about stomach contents [34]. This supports that green turtles use waters off South Korea as foraging areas in mainly warm season and travel to lower latitudes in the cold season.

KOR0104 was the only turtle that was presumed to have traveled to a nesting area, even though its destination, Hainan Island, has had no records of green turtle nesting in the past 37 years owing to coastal development [57,58]. Nevertheless, KOR0104 visited 12 of the 13 historical nesting areas on Hainan Island [58] and appeared to aim to nest regardless of its success. The Yaeyama and Ogasawara Islands, which are closer to the release site, are the largest nesting sites for green turtles in the Northwest Pacific Ocean [68], so the long travel of KOR0104 to Hainan Island suggests that this turtle may have originated in the southwestern Pacific. Previous genetic analyses using mtDNA have shown that green turtles in the Northwest Pacific Ocean are intermixed with turtles from Southeast Asian colonies [19,20,68]. Other studies have also reported the Taiwan Strait being used by other green turtles for travel [14,69]. Therefore, this route could be a major passage for green turtles, connecting the northern and southern parts of the western Pacific Ocean.

Comparison of the two sea turtle species

Both the loggerhead and green turtles mainly stayed in the East China Sea, while a few individuals used the East Sea, mainly in summer and autumn. Green turtles showed a wider distribution and more diverse travel patterns than loggerhead turtles, with one green turtle travelling to the South China Sea. Additionally, differences in the environmental variables between the foraging habitats of the two species were identified. Throughout the year, green turtles stayed in foraging areas within 26 km of the shoreline and used the areas with depths of less than 100 m, while loggerhead turtles used areas with depths of approximately 150 m from winter to spring and over

1,000 m in summer and autumn in the East Sea. In addition, loggerhead turtles stayed approximately 50 km away from the shoreline during winter and summer. Both adult loggerhead and green turtles are known to forage mainly in shallow coastal waters, where the depths reach between 20 and 50 m [50,66], although some turtles prefer open oceanic habitats [4,70]. In Korean waters, more loggerhead turtles are observed in the East Sea, whereas more green turtles are observed near Jeju Island [5,34,41]. Because two loggerhead turtles, KOR0092 and KOR0151, stayed in the East Sea for long periods, the values of Chla and POC in the foraging areas of loggerhead turtles were greater than those of green turtles. These interspecific differences suggest that loggerhead turtles are relatively better adapted to deeper waters and cooler sea temperatures than green turtles [52].

Conservation strategy

The information obtained through the tracked sea turtles expands our horizons on the travel patterns and habitat use of them in the north part of the Northwest Pacific Ocean, ~~the northern limit of their distribution range,~~ where little is known about the spatial ecology of sea turtles. The home range of sea turtles could be overestimated considering the adaptation to the sea and migration distances to suitable habitats caused by their rehabilitation period in artificial facilities. Nevertheless, the periodic behavior of tracked sea turtles and their response to environmental variables, especially SST, Chla, and POC, showed that the rescued turtles were rehabilitated well and are adapting to the sea. Our results revealed patterns of habitat use different from those reported in previous results [24,37,59]. Contrary to what has been previously reported, adult sea turtles did not migrate to the open Central Pacific Ocean in our study [19,41], and instead stayed in and used the East China Sea and East Sea around South Korea and Japan as their main foraging areas. Notably, this study marks the first time the East Sea (east coast of the Gangwon Province in South Korea) has been identified as a sea turtle foraging area. The low sea temperatures in the northern part of the East China Sea and East Sea have been reported to limit the habitat use of loggerhead and green sea turtles [34,37]. However, recent climate change causes a continuous increase in sea temperatures in the North Pacific Ocean, which could extend northward into the habitat [9,10,71]. During the past 50 years, the sea temperature around South Korea has increased by approximately 1.5°C, which is near three times greater than the global average increase [72,73]. Although some sea turtles were observed using the East Sea during warm summers and autumns in this study, the number of sea turtles that do so is expected to rapidly increase in following years due to climate change. The appearance of sea turtles in the coastal sea of South Korea and the

steady increase in the number of fishery bycatch records over the last 15 years [34,38,39] support this prediction. Therefore, it is necessary to continuously update the occurrence pattern of sea turtles in these areas and identify potential problems that sea turtles will face following population increases. Given that fishery bycatch is responsible for a global decline of sea turtles [7,74,75], protection policies for sea turtles and monitoring plans for fishing vessels should be prepared and applied at a national level. In conclusion, the increased interest in and implementation of management policies for sea turtle habitats in the northern part of the East China Sea and the East Sea near the Korean Peninsula and Japan could play a critical role in the conservation of sea turtles in the near future.

Acknowledgments

We thank Min-Woo Park and Hyerim Kwon for their assistance with data management.

References

1. Hudgins JA, Hudgins EJ, Köhnk S, Mohamed Riyad E, Stelfox MR. A brighter future? Stable and growing sea turtle populations in the Republic of Maldives. PLoS One 2023;18: e0283973. doi: 10.1371/journal.pone.0283973

2. Mazaris AD, Schofield G, Gkazinou C, Almpanidou V, Hays GC. Global sea turtle conservation successes. Sci Adv. 2017;3: e1600730. doi: 10.1126/sciadv.1600730.

~~1.~~3. Hamann M, Godfrey MH, Seminoff JA, Arthur K, Barata PCR, Bjorndal KA, et al. Global research priorities for sea turtles: informing management and conservation in the 21st century. Endang Species Res. 2010;11: 245-269. doi: 10.3354/esr00279.

~~2.~~4. Shimada T, Aoki S, Kameda K, Hazel J, Reich K, Kamezaki N. Site fidelity, ontogenetic shift and diet composition of green turtles Chelonia mydas in Japan inferred from stable isotope analysis. Endang Species Res. 2014;25: 151-164. doi: 10.3354/esr00616.

~~3.~~5. Oki K, Hamabata T, Arata T, Parker DM, Ng CKY, Balazs GH. Inferred adult foraging grounds of two marine turtle species nesting at Amami-Oshima, Japan. Chelonian Conserv Biol. 2019;18: 91-97. doi: 10.2744/CCB-1337.1.

~~4.~~6. Hatase H, Sato K, Yamaguchi M, Takahashi K, Tsukamoto K. Individual variation in feeding habitat use by adult female green sea turtles (*Chelonia mydas*): are they obligately neritic herbivores? Oecologia. 2006;149: 52-64. doi: 10.1007/s00442-006-0431-2.

~~5.~~7. Lewison RL, Crowder LB, Wallace BP, Moore JE, Cox T, Zydelis R, et al. Global patterns of marine mammal, seabird, and sea turtle bycatch reveal taxa-specific and cumulative megafauna hotspots. Proc Natl Acad Sci U S A. 2014;111: 5271-5276. doi: 10.1073/pnas.1318960111.

~~6.~~8. Putman NF, Hawkins J, Gallaway BJ. Managing fisheries in a world with more sea turtles. Proc Biol Sci. 2020;287: 20200220. doi: 10.1098/rspb.2020.0220.

~~7.~~9. Chaloupka M, Kamezaki N, Limpus C. Is climate change affecting the population dynamics of the endangered Pacific loggerhead sea turtle? J Exp Mar Biol Ecol. 2008;356: 136-143. doi: 10.1016/j.jembe.2007.12.009.

~~8.~~10. Abraham JP, Baringer M, Bindoff NL, Boyer T, Cheng LJ, Church JA, et al. A review of global ocean temperature observations: implications for ocean heat content estimates and climate change. Rev Geophys. 2013;51: 450-483. doi: 10.1002/rog.20022.

~~9.~~11. Garcia RA, Cabeza M, Rahbek C, Araújo MB. Multiple dimensions of climate change and their implications for biodiversity. Science. 2014;344: 1247579. doi: 10.1126/science.1247579.

12. Butt N, Whiting S, Dethmers K. Identifying future sea turtle conservation areas under climate change. Biol Conserv. 2016;204: 189-196. doi: 10.1016/j.biocon.2016.10.012.

13. Hays GC, Hawkes LA. Satellite tracking sea turtles: Opportunities and challenges to address key questions. Front Mar Sci. 2018;5: 432. doi: 10.3389/fmars.2018.00432.

~~10.~~14. Evans DR, Carthy RR, Ceriani SA. Migration routes, foraging behavior, and site fidelity of loggerhead sea turtles (*Caretta caretta*) satellite tracked from a globally important rookery. Mar Biol. 2019;166: 134. doi: 10.1007/s00227-019-3583-4.

~~11.~~15. Robinson DP, Hyland K, Beukes G, Vettan A, Mabadikate A, Jabado RW, et al. Satellite tracking of rehabilitated sea turtles suggests a high rate of short-term survival following release. PLoS One. 2021;16: e0246241. doi: 10.1371/journal.pone.0246241.

~~12.~~16. Kim IH, Park IK, Han DJ, Kim MS, Park D, Moon DY, et al. Movement patterns of juvenile loggerhead turtles (*Caretta caretta* L. 1758) and green turtles (*Chelonia mydas* L. 1758) hatched in captivity and released in the Korean Waters. Animals (Basel). 2022;12: 2157. doi: 10.3390/ani12162157.

~~13.~~17. Bowen BW. Tracking marine turtles with genetic markers. BioScience. 1995;45: 528-534. doi: 10.2307/1312697.

~~14.~~18. Hamabata T, Nishida S, Kamezaki NI, Koike H 2009. Genetic structure of populations of the green turtle (*Chelonia mydas*) in Japan using mtDNA control region sequences. *Bulletin of the graduate school of social and cultural studies. Kyushu University*, 15, 35-50.

~~15.~~19. Nishizawa H, Naito Y, Suganuma H, Abe O, Okuyama J, Hirate K, et al. Composition of green turtle feeding aggregations along the Japanese archipelago: implications for changes in composition with current flow. Mar Biol. 2013;160: 2671-2685. doi: 10.1007/s00227-013-2261-1.

~~16.~~20. Yang W, Wang Y, Chen M. Genetic structure and diversity of green sea turtle (Chelonia mydas) from South China Sea inferred by mtDNA control region sequence. Biochem Syst Ecol. 2015;60: 95-98. doi: 10.1016/j.bse.2015.04.007.

~~17.~~21. Loisier A, Savelli MP, Arnal V, Claro F, Gambaiani D, Sénégas JB, et al. Genetic composition, origin and conservation of loggerhead sea turtles (*Caretta caretta*) frequenting the French Mediterranean coasts. Mar Biol. 2021;168: 1-15. doi: 10.1007/s00227-020-03815-6.

~~18.~~22. Saito T, Kurita M, Okamoto H, Uchida I, Parker D, Balazs G. Tracking male loggerhead turtle migrations around southwestern Japan using satellite telemetry. Chelonian Conserv Biol. 2015;14: 82-87. doi: 10.2744/ccab-14-01-82-87.1.

~~19.~~23. Sato K. Decline of the loggerhead turtle, Caretta caretta, nesting on Senri Beach in Minabe, Wakayama, Japan. Chelonian Conserv Biol. 1997;2: 600-603.

~~20.~~24. Hatase H, Takai N, Matsuzawa Y, Sakamoto W, Omuta K, Goto K, et al. Size-related differences in feeding habitat use of adult female loggerhead turtles *Caretta caretta* around Japan determined by stable isotope analyses and satellite telemetry. Mar Ecol Prog Ser. 2002;233: 273-281. doi: 10.3354/meps233273.

~~21.~~25. Shimada T, Jones R, Limpus C, Groom R, Hamann M. Long-term and seasonal patterns of sea turtle home ranges in warm coastal foraging habitats: implications for conservation. Mar Ecol Prog Ser. 2016;562: 163-179. doi: 10.3354/meps11972.

~~22.~~26. Balazs GH, Parker DM, Rice MR. Ocean pathways and residential foraging locations for satellite tracked green turtles breeding at French Frigate Shoals in the Hawaiian Islands. Micronesica. 2017;4: 1-19.

~~23.~~27. Polovina J, Uchida I, Balazs G, Howell EA, Parker D, Dutton P. The Kuroshio Extension Bifurcation Region: a pelagic hotspot for juvenile loggerhead sea turtles. Deep Sea Res II. 2006;53: 326-339. doi: 10.1016/j.dsr2.2006.01.006.

~~24.~~28. Kobayashi DR, Polovina JJ, Parker DM, Kamezaki N, Cheng IJ, Uchida I, et al. Pelagic habitat characterization of loggerhead sea turtles, *Caretta caretta*, in the North Pacific Ocean (1997–

2006): insights from satellite tag tracking and remotely sensed data. J Exp Mar Biol Ecol. 2008;356: 96-114. doi: 10.1016/j.jembe.2007.12.019.

~~25.~~29. McCarthy AL, Heppell S, Royer F, Freitas C, Dellinger T. Identification of likely foraging habitat of pelagic loggerhead sea turtles (*Caretta caretta*) in the North Atlantic through analysis of telemetry track sinuosity. Prog Oceanogr. 2010;86: 224-231. doi: 10.1016/j.pocean.2010.04.009.

30. Mencacci R, De Bernardi E, Sale A, Lutjeharms JRE, Luschi P. Influence of oceanic factors on long-distance movements of loggerhead sea turtles displaced in the southwest Indian Ocean. Mar Biol. 2010;157: 339-349. doi: 10.1007/s00227-009-1321-z.

31. Hays GC, Bailey H, Bograd SJ, Bowen WD, Campagna C, Carmichael RH, et al. Translating marine animal tracking data into conservation policy and management. Trends Ecol. Evol. 2019;34: 459-473. doi: 10.1016/j.tree.2019.01.009.

~~26.~~32. Goris RC. Guide to the amphibian and reptiles of Japan. Florida: Krieger Publishing Company; 2004. pp. 131-136.

~~27.~~33. Chan SKF, Cheng IJ, Zhou T, Wang HJ, Gu HX, Song XJ. A comprehensive overview of the population and conservation status of sea turtles in China. Chelonian Conserv Biol. 2007;6: 185-198. doi: 10.2744/1071-8443(2007)6[185:ACOOTP]2.0.CO;2.

~~28.~~34. Kim IH, Moon DY, Cho IY, Kim MS, An YR, Han D, et al. Occurrence of sea turtles in the Korean Waters and the morphological characteristics of two major species. Korean J Fish Aquat Sci. 2017;50: 311-318. Doi: 10.5657/KFAS.2017.0311.

~~29.~~35. Hatase H, Matsuzawa Y, Sakamoto W, Baba N, Miyawaki I. Pelagic habitat use of an adult Japanese male loggerhead turtle *Caretta caretta* examined by the Argos satellite system. Fisheries Sci. 2002a;68: 945-947. doi: 10.1046/j.1444-2906.2002.00515.x.

~~30.~~36. Narazaki T, Sato K, Miyazaki N. Summer migration to temperate foraging habitats and active winter diving of juvenile loggerhead turtles *Caretta caretta* in the western North Pacific. Mar Biol. 2015;162: 1251-1263. doi: 10.1007/s00227-015-2666-0.

37. Hamabata T, Nishizawa H, Kawazu I, Kameda K, Kamezaki N, Hikida T. Stock composition of green turtles *Chelonia mydas* foraging in the Ryukyu Archipelago differs with size class. Mar Ecol Prog Ser. 2018;600: 151-163. doi: 10.3354/meps12657.

38. Moon DY, Jung MM, An YR, Choi SG, Oh BS, Kim ZG, et al. Distribution and strandings of endangered sea turtles in Korean Waters. Korean J Fish Aquat Sci. 2009;42: 657-663. doi: 10.5657/kfas.2009.42.6.657.

31.39. Jung MM, Moon DY, Kim SH, Kim HS, Kim JW. Observation and record of sea turtles in bycatch and stranding from Jeju Island of Korea. J Fish Mar Sci Educ. 2012;24: 662-669. doi: 10.13000/JFMSE.2012.24.5.662.

32.40. Saito T, Kurita M, Okamoto H, Kakizoe Y, Parker D, Briscoe D, et al. Satellite tracking immature loggerhead turtles in temperate and subarctic ocean habitats around the Sea of Japan. Micronesica. 2018;3: 1-20.

33.41. Jang S, Balazs GH, Parker DM, Kim BY, Kim MY, Ng CKY, et al. Movements of green turtles (*Chelonia mydas*) rescued from pound nets near Jeju Island, Republic of Korea. Chelonian Conserv Biol. 2018;17: 236-244. doi: 10.2744/CCB-1279.1.

34.42. Green D. Growth rates of wild immature green turtles in the Galapagos Islands, Ecuador. J Herpetol. 1993;27: 338-341. doi: 10.2307/1565159.

35.43. Ishihara T, Kamezaki N. Size at maturity and tail elongation of loggerhead turtles (Caretta caretta) in the North Pacific. Chelonian Conserv Biol. 2011;10: 281-287. doi: 10.2744/CCB-0893.1.

44. Robinson DP, Jabado RW, Rohner CA, Pierce SJ, Hyland KP, Baverstock WR. Satellite tagging of rehabilitated green sea turtles *Chelonia mydas* from the United Arab Emirates, including the longest tracked journey for the species. PLoS One. 2017;12: e0184286. doi: 10.1371/journal.pone.0184286.

45. Witt MJ, Åkesson S, Broderick AC, Coyne MS, Ellick J, Formia A, et al. Assessing accuracy and utility of satellite-tracking data using Argos-linked Fastloc-GPS. Anim Behav. 2010;80: 571. doi: 10.1016/j.anbehav2010.05.022.

46. Thomson JA, Börger L, Christianen MJA, Esteban N, Laloë JO, Hays GC. Implications of location accuracy and data volume for home range estimation and fine-scale movement analysis: comparing Argos and Fastloc-GPS tracking data. Mar Biol. 2017;164: 1-9. doi: 10.1007/BF00397131.

47. Balazs GH, Miya RK, Beavers S. Procedures to attach a satellite transmitter to the carapace of an adult green turtle, *Chelonia mydas*. NOAA Tech. Memorandum NMFS-SEFSC; 1996. pp. 21–26.

48. Seney, EE, Higgins BM & Landry Jr AM. Satellite transmitter attachment techniques for small juvenile sea turtles. J Exp Mar Biol Ecol. 2010;384: 61-67. doi: 10.1016/j.jembe.2010.01.002.

49. Hays GC, Åkesson S, Godley BJ, Luschi P, Santidrian P. The implications of location accuracy for the interpretation of satellite-tracking data. Anim Behav. 2001;61: 1035-1040.

~~36.~~50. Hart KM, Lamont MM, Fujisaki I, Tucker AD, Carthy RR. Common coastal foraging areas for loggerheads in the Gulf of Mexico: opportunities for marine conservation. Biol Conserv. 2012;145: 185-194. doi: 10.1016/j.biocon.2011.10.030.

51. Zbinden JA, Aebischer A, Margaritoulis D, Arlettaz R. Important areas at sea for adult loggerhead sea turtles in the Mediterranean Sea: satellite tracking corroborates findings from potentially biased sources. Mar Biol. 2008;153: 899-906. doi: 10.1007/s00227-007-0862-2.

52. Schofield G, Hobson VJ, Fossette S, Lilley MK, Katselidis KA, Hays GC. Fidelity to foraging sites, consistency of migration routes and habitat modulation of home range by sea turtles. Divers Distrib. 2010;16: 840-853. doi: 10.1111/j.1472-4642.2010.00694.x.

~~37.~~53. Coyne MS, Godley BJ. Satellite Tracking and Analysis Tool (STAT): an integrated system for archiving, analyzing and mapping animal tracking data. Mar Ecol Prog Ser. 2005;301: 1-7. doi: 10.3354/meps301001.

~~38.~~54. Evrard V, Kiswara W, Bouma TJ, Middelburg JJ. Nutrient dynamics of seagrass ecosystems: 15N evidence for the importance of particulate organic matter and root systems. Mar Ecol Prog Ser. 2005;295: 49-55. doi: 10.3354/meps295049.

~~39.~~55. Wickham H. ggplot2: elegant graphics for data analysis. Springer; 2016.

~~40.~~56. R Core Team. R: A language and environment for statistical computing. R Foundation for Statistical Computing; 2021.

~~41.~~57. Wang YM. Achievement and perspective of the researches on South China Sea turtle resources and protection in China. Chin J Ecol. 1993;12: 60-61.

~~42.~~58. Zhang T, Lin L, Li M, Kong L, Wang J, Shi HT. Investigation on beach debris on the historical nesting grounds of green turtles (*Chelonia mydas*) in Hainan Island, South China. Heliyon. 2023;9: e13400. doi: 10.1016/j.heliyon.2023.e13400.

~~43.~~59. Okuyama J, Watabe A, Takuma S, Tanaka K, Shirai K, Murakami- Sugihara N, et al. Latitudinal cline in the foraging dichotomy of loggerhead sea turtles reveals the importance of East China Sea for priority conservation. Divers Distrib. 2022;28: 1568-1581. doi: 10.1111/ddi.13531.

~~44.~~60. Kameda K, Wakatsuki M, Takase M, Nakanishi Y, Kamezaki N. Apparent survival probability and abundance of juvenile green turtles in the foraging ground at Kuroshima Island, Ryukyu Archipelago. Endang Species Res. 2023;50: 209-215. doi: 10.3354/esr01228.

~~45.~~61. Kikukawa A, Kamezaki N, Ota H. Current status of the sea turtles nesting on Okinawajima and adjacent islands of the central Ryukyus, Japan. Biol Conserv. 1999;87: 149-153. doi: 10.1016/S0006-3207(98)00047-0.

~~46.~~62. Kawazu I, Okabe H, Kobayashi N. Direct observation of mating behavior involving one female and two male loggerhead turtles in the wild. Curr Herpetol. 2017;36: 69-72. doi: 10.5358/hsj.36.69.

~~47.~~63. Kim J, Kim IH, Kim MS, Lee HR, Kim YJ, Park S, et al. Occurrence and diet analysis of sea turtles in Korean shore. J Ecol Environ. 2021;45: 1-15. doi: 10.1186/s41610-021-00206-w.

~~48.~~64. KHOA (Korea Hydrographic and Ocenographic Agency). Analysis of sea surface temperature in the coast of Korean Peninsula using satellite images; 2016. [Cited June 22, 2023]. Available from: http://www.khoa.go.kr/eng/on.

~~49.~~65. Kinoshita C, Fukuoka T, Niizuma Y, Narazaki T, Sato K. High resting metabolic rates with low thermal dependence induce active dives in overwintering Pacific juvenile loggerhead turtles. J Exp Biol. 2018;221: jeb175836. doi: 10.1242/jeb.175836.

66. Pilcher NJ, Antonopoulou MA, Rodriguez-Zarate CJ, Mateos-Molina D, Das HS, Bugla I, et al. Movements of green turtles from foraging areas of the United Arab Emirates: regional habitat connectivity and use of marine protected areas. Mar Biol. 2021;168: 1-15. doi: 10.1007/s00227-020-03815-6.

~~50.~~67. Esteban N, Mortimer JA, Stokes HJ, Laloë JO, Unsworth RK, Hays GC. A global review of green turtle diet: sea surface temperature as a potential driver of omnivory levels. Mar Biol 2020;167: 1-17. Doi: 10.1007/s00227-020-03786-8.

~~51.~~68. Nishizawa H, Abe O, Okuyama J, Kobayashi M, Arai N. Population genetic structure and implications for natal philopatry of nesting green turtles Chelonia mydas in the Yaeyama Islands, Japan. Endang Species Res. 2011;14: 141-148. doi: 10.3354/esr00355.

~~52.~~69. Ng CKY, Dutton PH, Gu HX, Li TH, Ye MB, Xia ZR, et al. Regional conservation implications of green turtle (*Chelonia mydas*) genetic stock composition in China. Chelonian Conserv Biol. 2017;16: 139-150. doi: 10.2744/CCB-1253.1.

~~53.~~70. McClellan CM, Read AJ. Complexity and variation in loggerhead sea turtle life history. Biol Lett. 2007;3: 592-594. doi: 10.1098/rsbl.2007.0355.

~~54.~~71. Overland JE, Wang M. Future climate of the north Pacific Ocean. Eos Trans AGU. 2007;88: 178-182. doi: 10.1029/2007EO160003.

~~55.~~72. Kim S, Woo S, Kim B, Hur S. Trends in sea surface temperature (SST) change near the Korean peninsula for the past 130 years. Ocean Polar Res. 2011;33: 281-290. doi: 10.4217/OPR.2011.33.3.281.

~~56.~~73. Han IS, Lee JS. Change the annual amplitude of sea surface temperature due to climate change in a recent decade around the Korean Peninsula. J Korean Soc Mar Environ Saf. 2020;26: 233-241. doi: 10.7837/kosomes.2020.26.3.233.

~~57.~~74. Finkbeiner EM, Wallace BP, Moore JE, Lewison RL, Crowder LB, Read AJ. Cumulative estimates of sea turtle bycatch and mortality in USA fisheries between 1990 and 2007. Biol Conserv. 2011;144: 2719-2727. doi: 10.1016/j.biocon.2011.07.033.

~~58.~~75. Wallace BP, Kot CY, DiMatteo AD, Lee T, Crowder LB, Lewison RL. Impacts of fisheries bycatch on marine turtle populations worldwide: toward conservation and research priorities. Ecosphere. 2013;4: 1-49. doi: 10.1890/ES12-00388.1.

Table 1. Physical characteristics and tracking details of the tracked loggerhead and green turtles tracked.

ID	CCL (cm)	BW (kg)	Tracking duration (days)	Distance traveled (km)		Home range (1,000 km^2)		
				Total	Daily	MCP	95 PVC	50 PVC
Loggerhead turtle								
KOR0001	74.0	55.0	18/06/2016 - 23/07/2016 (35)	129.8	5.8 ± 3.8	0.4	0.4	0.0
KOR0008	76.0	56.0	28/09/2017 - 24/12/2017 (87)	2,862.2	37.9 ± 37.2	417.7	15.4	1.4
KOR0091	79.9	52.6	29/08/2018 - 13/10/2018 (45)	1,055.2	24.0 ± 14.1	36.2	41.5	5.7
KOR0092	91.3	85.2	17/10/2018 - 11/09/2019 (329)	6,217.0	18.8 ± 15.7	253.9	164.3	30.7
KOR0149	73.2	82.2	26/08/2021 - 12/12/2021 (108)	1,756.5	18.4 ± 11.4	50.4	85.4	15.3
KOR0151	73.0	77.8	26/08/2021 - 09/10/2022 (409)	8,964.8	22.2 ± 14.4	489.9	333.5	23.2
KOR0155	71.0	69.7	25/08/2022 - 27/09/2022 (33)	598.5	18.1 ± 9.3	20.9	30.3	5.5
Mean	76.9 ± 6.9	68.4 ± 13.8	(149.5 ± 154.2)	3,083.4 ± 3,297.3	20.7 ± 9.5	181.3 ± 205.4	95.8 ± 118.4	11.7 ± 11.7
Green turtle								
KOR-1	65.6	31.8	29/10/2015 – 15/12/2015 (47)	1,601.5	32.8 ± 23.0	133.1	30.7	5.3
KOR-2	60.0	22.5	29/10/2015 - 29/10/2016 (366)	1,461.3	6.2 ± 11.9	41.8	15.8	6.0
KOR0003	95.8	103.0	12/08/2016 - 17/10/2016 (66)	1,291.7	25.5 ± 26.0	75.1	25.1	7.1
KOR0004	68.0	41.4	02/09/2016 - 26/10/2016 (54)	1,452.3	26.3 ± 17.4	162.1	145.4	20.7
KOR0009	78.8	58.9	28/09/2017 - 28/09/2018 (365)	6,484.3	18.7 ± 17.3	221.7	140.3	14.3
KOR0104	77.6	69.8	07/10/2018 - 01/05/2019 (206)	5,203.5	27.5 ± 22.1	1,056.8	246.9	4.3
KOR0129	77.3	70.9	28/08/2019 - 26/10/2019 (59)	2,412.4	43.5 ± 33.0	221.2	244.8	34.2
KOR0148	70.4	79.2	26/08/2021 - 26/07/2022 (334)	1,623.0	7.5 ± 12.5	34.7	47.3	6.6
Mean	74.2 ± 10.9	59.7 ± 26.7	(187.2 ± 148.4)	2,691.3 ± 2,004.0	23.5 ± 12.5	243.3 ± 336.8	112.0 ± 96.6	12.3 ± 10.5

CCL = curved carapace length; BW = body weight; MCP = minimum convex polygon; PVC = percentage volume contour.

Table 2. Summary of the six environmental variables measured in this study.

Environmental variable	Resolution	Frequency	Source
Distance to shore (km)			
Bathymetry (m)	~ 1 km		GEBCO (https://www.gebco.net/)
Sea surface temperature (°C)	~ 4 km	8-day mean	NASA OceanColor Web (https://oceancolor.gsfc.nasa.gov)
Sea surface salinity (‰)	~ 40 km	8-day mean	EarthData (https://earthdata.nasa.gov)
Chlorophyll-a (mg/m^3)	~ 4 km	8-day mean	NASA OceanColor Web (https://oceancolor.gsfc.nasa.gov)
Particulate organic carbon (mol/m^3)	~ 4 km	8-day mean	NASA OceanColor Web (https://oceancolor.gsfc.nasa.gov)

Table 3. Mean value ± standard deviation of the six environmental variables measured at the transitional~~veling~~ and foraging areas of each sea turtle.

ID	Type of area	Distance to shore (km)	Bathymetry (m)	SST (°C)	SSS (‰)	Chla (mg/m^3)	POC (mol/m^3)
Loggerhead turtle							
KOR0001	T	3.2 ± 3.3	21.2 ± 13.8	22.8 ± 1.2	31.4 ± 1.7	1.76 ± 0.94	230.8 ± 76.4
	F~~oraging~~	-	-	-	-	-	-
KOR0008	T~~ravel~~	129.4 ± 78.0	1,795.4 ± 2,110.3	26.4 ±1.7	33.5 ± 1.4	0.49 ± 0.7	112.7 ± 165.4
	F~~oraging~~	2.0 ± 1.3	13.6 ± 18.3	24.0 ± 1.1	34.8 ±1.0	0.22 ±0.10	77.4 ± 25.0
KOR0091	T~~ravel~~	43.8 ± 49.1	85.6 ± 22.3	24.6 ± 1.8	32.5 ± 1.4	0.77 ± 0.37	140.4 ± 41.5
	F~~oraging~~	-	-	-	-	-	-
KOR0092	T~~ravel~~	58.6 ± 38.3	363.6 ± 549.7	21.4 ± 2.6	33.1 ± 1.4	0.52 ± 0.41	113.1 ± 66.8
	F~~oraging~~	83.6 ± 55.5	119.3 ± 30.2	19.0 ± 2.1	34.0 ± 1.3	0.49 ± 0.25	118.0 ± 46.6
KOR0149	T~~ravel~~	124.7 ± 74.7	65.1 ± 19.8	21.4 ±3.4	31.2 ± 0.9	1.62 ± 0.70	232.1 ± 78.0
	F~~oraging~~	89.1 ± 22.9	79.5 ± 9.7	22.6 ± 2.9	31.5 ± 0.8	1.18 ± 0.63	195.1 ± 79.6
KOR0151	T~~ravel~~	32.4 ± 30.1	304.1 ± 502.3	20.0 ± 4.1	33.1 ± 2.1	0.44 ± 0.44	101.6 ± 61.1
	F~~oraging~~	42.4 ± 38.1	733.9 ± 557.0	22.4 ± 3.2	32.5 ± 1.6	0.69 ± 0.46	163.1 ± 112.4
KOR0155	T~~ravel~~	85.1 ± 67.7	87.8 ± 33.1	26.8 ± 2.2	31.5 ± 1.1	0.64 ± 0.47	122.8 ± 53.2
	F~~oraging~~	-	-	-	-	-	-
Mean	T~~ravel~~	68.2 ± 47.3	389.1 ± 633.5	23.4 ± 2.7	32.3 ± 0.9	0.89 ± 0.56	150.5 ± 56.5
	F~~oraging~~	54.3 ± 40.6	236.6 ± 334.4	22.0 ± 2.1	33.2 ± 1.5	0.64 ± 0.41	138.4 ± 51.5
Green turtle							
KOR-1	T~~ravel~~	9.7 ± 13.6	58.4 ± 42.8	21.1 ± 1.4	31.5 ± 3.1	1.60 ± 1.89	167.1 ± 81.8
	F~~oraging~~	-	-	-	-	-	-
KOR-2	T~~ravel~~	6.4 ± 8.2	71.3 ± 80.9	20.2 ± 1.5	33.0 ± 1.1	0.95 ± 0.88	166.0 ± 103.1
	F~~oraging~~	1.5 ± 1.8	23.5 ± 20.1	23.9 ± 3.7	33.6 ± 1.4	0.26 ± 0.11	69.1 ± 22.6
KOR0003	T~~ravel~~	9.0 ± 15.9	57.2 ± 47.2	26.9 ± 2.2	31.6 ± 3.1	0.7 ± 0.4	124.9 ± 39.5
	F~~oraging~~	1.3 ± 1.2	27.9 ± 17.7	23.8 ± 1.0	31.7 ± 0.4	0.63 ± 0.15	121.9 ± 27.6
KOR0004	T~~ravel~~	131.2 ± 93.0	189.7 ± 278.0	26.6 ± 0.9	32.4 ± 1.6	0.58 ± 0.34	104.8 ± 33.7
	F~~oraging~~	-	-	-	-	-	-
KOR0009	T~~ravel~~	58.0 ± 46.0	465.1 ± 563.8	19.6 ± 3.8	33.6 ± 1.3	0.68 ± 0.81	123.0 ± 97.1
	F~~oraging~~	26.9 ± 27.4	84.7 ± 80.9	21.7 ± 4.3	33.8 ± 1.4	0.95 ± 0.74	167.6 ± 94.2
KOR0104	T~~ravel~~	32.2 ± 41.4	33.0 ± 27.9	23.5 ± 2.2	32.9 ± 1.6	1.59 ± 1.02	211.4 ± 89.0
	F~~oraging~~	-	-	-	-	-	-
KOR0129	T~~ravel~~	81.0 ± 67.4	52.1 ± 40.1	25.2 ± 1.5	30.6 ± 2.9	1.84 ± 1.08	230.9 ±91.5
	F~~oraging~~	-	-	-	-	-	-
KOR0148	T~~ravel~~	17.1 ± 21.0	191.6 ± 207.2	18.5 ± 1.5	32.4 ± 1.6	0.46 ± 0.24	105.3 ± 36.7
	F~~oraging~~	1.5 ± 1.5	82.7 ± 156.4	21.8 ± 4.1	33.4 ± 1.6	0.50 ± 0.51	111.0 ± 59.9
Mean	T~~ravel~~	43.1 ± 44.5	139.8 ± 145.5	22.7 ± 3.3	32.3 ± 1.0	1.06 ± 0.54	154.2 ± 48.0
	F~~oraging~~	7.8 ± 12.7	54.8 ± 33.6	22.8 ± 1.2	33.1 ± 1.0	0.59 ± 0.29	104.6 ± 50.1

SST = sea surface temperature; SSS = sea surface salinity; Chla = Chlorophyll-a; POC = particulate organic carbon; T = transitional area; F = foraging area.

Table 4. Comparison of the six environmental variables within and between species. Interspecific differences were only compared in foraging areas corresponding to both species, while the columns with each species title indicate differences in the variables between the foraging and travel areas using t-test. Asterisk indicates significant differences (p-value < 0.05).

Variables	Interspecific foraging use	Loggerhead turtle	Green turtles
DDT	t = 15.314<br>p < 0.001*	t = 6.997<br>p < 0.01*	t = 21.294<br>p < 0.001*
DTS	t = 24.622<br>p < 0.001*	t = -2.104<br>p < 0.05*	t = 16.249<br>p < 0.001*
Bathy	t = -14.767<br>p < 0.001*	t = -0.587<br>p > 0.05	t = -8.840<br>p < 0.001*
SST	t = -6.281<br>p < 0.001*	t = 2.878<br>p < 0.01*	t = -1.516<br>p > 0.05
SSS	t = -4.208<br>p < 0.001*	t = -2.718<br>p < 0.01*	t = -8.013<br>p < 0.001*
Chla	t = 3.300<br>p = 0.001*	t = -0.210<br>p > 0.05	t = 11.307<br>p < 0.001*
POC	t = 6.411<br>p < 0.001*	t = - 3.479<br>p < 0.01*	t = 9.751<br>p < 0.001*

Fig 1. Summarized travel patterns of the sea turtles released at the coastal seas of South Korea. White circles and squares with each color indicate start and end points, respectively. (A) seven loggerhead and (B) eight green turtles. This map was generated based on GADM data (https://dadm.org/).

Fig 2. Travel patterns and core habitat of the seven satellite-tracked loggerhead turtles. **Green zones indicate foraging areas calculated by 50 PVC. Dotted line areas indicate 50 PVC zones that were excluded from the core habitat owing to short stay duration. Dates marked with asterisks correspond to the year following the year of release. The red and blue arrows indicate the start and end dates of the tracking period, respectively.** This map was generated based on GADM data (https://dadm.org/).

Fig 3. Travel patterns and core habitat of the eight satellite-tracked green turtles. Green and red zones indicate foraging and nesting areas calculated by 50 PVC, respectively. Dotted line areas indicate 50 PVC zones that were excluded from the core habitat owing to short stay duration. Dates marked with asterisks correspond to the year following the year of release. The red and blue arrows indicate the start and end dates of the tracking period, respectively. This map was generated based on GADM data (https://dadm.org/).

Fig 4. Monthly changes in latitude, daily distance traveled, and six environmental variables (mean ± standard error) measured in the foraging areas of the both sea turtle species.

Fig 5. Travel routes and environmental variables on the travel route of two representative loggerhead turtles. Green zones on the maps and the dotted sections in the graphs indicate core areas by 50 PVC. POC = particulate organic carbon. This map was generated based on GADM data (https://dadm.org/).

Fig 6. Travel routes and environmental variables on the travel route of two representative green turtles. Green and pink zones on the maps and the dotted sections in the graphs indicate core areas by 50 PVC. POC = particulate organic carbon. This map was generated based on GADM data (https://dadm.org/).

Dear Section Editor of PLOS ONE,

We appreciate your valuable edits and suggestions on our manuscript. Most comments were reasonable and made our research more rigorous and valuable. We have considered all of the reviewers' comments and have revised the manuscript accordingly. All our edits can be viewed in the "Track Changes". The response answers to the reviewers' comments are written in blue text below.

Response to Editor

When submitting your revision, we need you to address these additional requirements.

1. Please ensure that your manuscript meets PLOS ONE's style requirements, including those for file naming. The PLOS ONE style templates can be found at https://journals.plos.org/plosone/s/file?id=wjVg/PLOSOne_formatting_sample_main_body.pdf and https://journals.plos.org/plosone/s/file?id=ba62/PLOSOne_formatting_sample_title_authors_affiliations.pdf

A. We wrote the manuscript considering the author guideline and above details.

2. Thank you for stating the following financial disclosure:
"This study was supported by grants from the National Marine Biodiversity Institute of Korea (2023M00300 and 2023E00300) and the 'Development of Technology for Impact Assessment of Marine Plastic Debris on Marine Ecosystem (EA0114)" funded by the Korea Institute of Ocean Science and Technology."

   Please state what role the funders took in the study. If the funders had no role, please state: "The funders had no role in study design, data collection and analysis, decision to publish, or preparation of the manuscript." If this statement is not correct you must amend it as needed.
   Please include this amended Role of Funder statement in your cover letter; we will change the online submission form on your behalf.

A. The three directors who secured the Funders participated in the project administration, supervision and investigation. They contributed to the secure and release of sea turtles and data management.

3. PLOS requires an ORCID iD for the corresponding author in Editorial Manager on papers submitted after December 6th, 2016. Please ensure that you have an ORCID iD and that it is validated in Editorial Manager. To do this, go to 'Update my Information' (in the upper left-hand corner of the main menu), and click on the Fetch/Validate link next to the ORCID field. This will take you to the ORCID site and allow you to create a new iD or authenticate a pre-existing iD in Editorial Manager. Please see the following video for instructions on linking an ORCID iD to your Editorial Manager account: https://www.youtube.com/watch?v=_xcclfuvtxQ

A. Yes, corresponding author (An YR: 0000-0002-0218-6254) has joined ORCID and added information.

4. Please include your full ethics statement in the 'Methods' section of your manuscript file. In your statement, please include the full name of the IRB or ethics committee who approved or waived your study, as well as whether or not you obtained informed written or verbal consent. If consent was waived for your study, please include this information in your statement as well.

A. We've added it into the manuscript.

=> Permissions for the sea turtle conservation program were given by the Marine Animal Protection Committee of the Ministry of Oceans and Fisheries with whom this work was conducted.

5. We note that [Figures 1,2,3,5, and 6] in your submission contain [map/satellite] images which may be copyrighted. All PLOS content is published under the Creative Commons Attribution License (CC BY 4.0), which means that the manuscript, images, and Supporting Information files will be freely available online, and any third party is permitted to access, download, copy, distribute, and use these materials in any way, even commercially, with proper attribution. For these reasons, we cannot publish previously copyrighted maps or satellite images created using proprietary data, such as Google software (Google Maps, Street View, and Earth). For more information, see our copyright guidelines: http://journals.plos.org/plosone/s/licenses-and-copyright.

- PLOS ONE: Edits requested on your submission PONE-D-23-24778R1 - [EMID:645d75582986089b]

A. We made Figures 1,2,3,5, and 6 based on GADM (Database of Global Administrative Areas; https://gadm.org/) data. This data could be used for academic research, including PLos journal. We attached the licence from the GADM website (https://gadm.org/license.html).

6. Thank you for updating your data availability statement. You note that your data are available within the Supporting Information files, but no such files have been included with your submission. At this time we ask that you please upload your minimal data set as a Supporting Information file, or to a public repository such as Figshare or Dryad. Please also ensure that when you upload your file you include separate captions for your supplementary files at the end of your manuscript. As soon as you confirm the location of the data underlying your findings, we will be able to proceed with the review of your submission.

- PLOS ONE: Edits requested on your submission PONE-D-23-24778R1 - [EMID:645d75582986089b]

A. We have uploaded the sea turtle migration data to movebank as requested. We will compress the relevant files into a ZIP file and submit it to the Supporting Information - Compressed/ZIP File Archive section.

https://www.movebank.org/movebank/#page=studies,path=study3030282201

Response to Reviewer 1

1. Line 42. I think you need to be a little more honest and say that in many area turtle numbers are increasing, thanks to conservation efforts and so in the latest IUCN regional assessments not all populations are endangered. E.g. see: Mazaris AD et al. (2017). Global sea turtle conservation successes. Science Advances 3: e1600730. https://doi.org/10.1126/sciadv.1600730
   If you look on the IUCN website at the latest regional assessments, you will see that in some areas turtles are not listed as endangered.

A: As your advice, we changed the first sentence in Introduction. The IUCN assessment criteria is separated into three threatened grades: Vulnerable, Endangered, and Critically Endangered. Therefore, we changed "endangered" to "threatened". => "Although many regional sea turtle populations are increasing thanks to conservation efforts [1,2], still six out of all seven known sea turtle species are threatened worldwide …"

-1: Hudgins JA, Hudgins EJ, Köhnk S, Mohamed Riyad E, Stelfox MR. A brighter future? Stable and growing sea turtle populations in the Republic of Maldives. PLoS One 2023;18: e0283973. doi: 10.1371/journal.pone.0283973

- 2: Mazaris AD, Schofield G, Gkazinou C, Almpanidou V, Hays GC. Global sea turtle conservation successes. Sci Adv. 2017;3: e1600730. doi: 10.1126/sciadv.1600730.

2. Line 49. "expanding north". I think you mean "polewards". In the southern hemisphere species are heading south.

A: we agreed it and revised the sentence.

3. Line 51. "telemetry is a useful method for investigating the spatial ecology and habitat use of sea turtles …" I would simply cite a recent review: "Satellite tracking sea turtles: opportunities and challenges to address key questions. https://doi.org/10.3389/fmars.2018.00432"

A: Because the recommended paper supports this content properly, we cited this paper.

4. Lines 59. "Overall, the habitat information and environmental characteristics identified by satellite telemetry could contribute to the effective protection and restoration of sea turtle populations …"
   Perhaps the best synthesis of how satellite tracking has helped drive turtle conservation is within this review: "Translating marine animal tracking data into conservation policy and management. Trends in Ecology and Evolution 34, 459-473. https://doi.org/10.1016/j.tree.2019.01.009"

A: Because the recommended paper supports this content properly, we cited this paper.

5. Line 86. "Considering the minimum CCL of sexually mature sea turtles is known as 73 cm in loggerhead turtles and 67 cm in green turtles …"

   One of your green turtles was 96cm. I find it hard to believe there could be such a massive range of adult sizes from 67-96cm. I suggest you discuss whether any of these turtles were actually sub-adults.

A: In many studies, the criteria for determining whether a sea turtle is an adult or subadult are very diverse. In Green (1993), green turtles with a CCL of 67 cm or more were classified as adults because they could breed. Additionally, the growth rate of green turtles tends to decrease rapidly from a CCL of 65 cm (Limpus and Chaloupka 1997). We categorized growth (adult or subadult) to determine whether the individual used the habitat as a foraging or nesting site.

- Green D. 1993. Growth rates of wild immature green turtles in the Galapagos Islands, Ecuador. J Herpetol. 27: 338-341.
- Limpus C & Chaloupka M. 1997. Nonparametric regression modelling of green sea turtle growth rates (southern Great Barrier Reef). Mar Ecol Prog Ser. 149: 23-34.

6. Normally green turtles feed on seagrass or macroalgae. There are recent reviews on their diet (https://doi.org/10.1007/s00227-020-03786-8). In this context I would discuss how many of the tracked turtles foraged in shallow coastal areas versus being in deep oceanic areas.

A: We estimated feeding sites based on sea turtles' migration patterns and environmental characteristics, but since we did not conduct research directly related to feeding, we believe it is difficult to definitively determine feeding sites. Instead, since there was previous research on the food items of green turtles near South Korea, we added related content based on the research you provided.

- ⇨ Additionally, green turtles consume animal matter more than seagrass at high latitudes or low SST areas under 25°C [67]. However, although green turtles generally used areas below SST of 23°C in our study, the most abundant food item was seaweed according to previous research about stomach contents [34]. This supports that green turtles use waters off South Korea as foraging areas in mainly warm season and travel to lower latitudes in the cold season.

-67: Esteban N, Mortimer JA, Stokes HJ, Laloë JO, Unsworth RK, Hays GC. A global review of green turtle diet: sea surface temperature as a potential driver of omnivory levels. Mar Biol 2020;167: 1-17. Doi: 10.1007/s00227-020-03786-8.

-34: Kim IH, Moon DY, Cho IY, Kim MS, An YR, Han D, et al. Occurrence of sea turtles in the Korean Waters and the morphological characteristics of two major species. Korean J Fish Aquat Sci. 2017;50: 311-318. Doi: 10.5657/KFAS.2017.0311.

7. Table 3. You talk about "travelling" and "foraging". How did you distinguish these 2 different states?

A: We determined the foraging and nesting or transitional (previous travelling) areas using 50 PVC and speed difference. First, 50 PVC indicate a relatively high-density area. After that, we will define a 50 PVC area as a foraging area only if the location data within 50 PVCs has a movement speed that is significantly lower than the outside, considering the slower movement speed of sea turtles in the foraging area. We statistically compared the daily distance traveled (DDT) for 50 PVC and outside points. The related information is presented in S2 Table.

- ⇨ We compared the DDT of location data inside and outside 50 PVC, and defined the 50 PVC as a foraging area if the DDT inside was significantly smaller, based on the slow travel speed of sea turtles in their foraging areas [51,52]. On the other hand, we defined all areas outside 50 PVC and where DDT was not significantly small as transitional areas.
- ⇨ To distinguish between the foraging or nesting area and the transitional area, we used an independent t-test, a one-way ANOVA test, and the Kruskal-Wallis test. First, we conducted the Shapiro-Wilkinson test with DDT value of each sea turtle for normality. When only one 50 PVC was generated in a sea turtle individual, the inside and outside DDT were compared using a t-test. If a sea turtle has multiple 50 PVCs, we conducted one-way ANOVA or Kruskal-Wallis test on the inside and outside of each 50 PVC. Whenever significant differences were detected, we defined only 50 PVC that had significantly small DDT compared to the outside as the foraging or nesting area by the Bonferroni or Dunn post hoc test.

- 51: Zbinden JA, Aebischer A, Margaritoulis D, Arlettaz R. Important areas at sea for adult loggerhead sea turtles in the Mediterranean Sea: satellite tracking corroborates findings from potentially biased sources. Mar Biol. 2008;153: 899-906. doi: 10.1007/s00227-007-0862-2.

- 52: Schofield G, Hobson VJ, Fossette S, Lilley MK, Katselidis KA, Hays GC. Fidelity to foraging sites, consistency of migration routes and habitat modulation of home range by sea turtles. Divers Distrib. 2010;16: 840-853. doi: 10.1111/j.1472-4642.2010.00694.x.

8. Lines 102. "… classes A and B, with no estimated accuracy".

This is not true.
Class A and B accuracy has been assessed in various studies. See for example:

Witt MJ et al. (2010). Assessing accuracy and utility of satellite-tracking data using Argos-linked Fastloc-GPS. Animal Behaviour 80, 571-581. doi:10.1016/j.anbehav.2010.05.022
Hays GC et al. (2001). The implications of location accuracy for the interpretation of satellite tracking data. Animal Behaviour 61, 1035-1040.

These studies show that Class A locations are good.
The worst accuracy is class 0 locations.
So I think you need to omit class 0 locations and include class A locations.
Also note that now Argos can provide Kalman filtered locations. The users decides. So specify whether you used Kalman filtered locations or not.

A: Generally, the order of accuracy is known to be LC 0, A, and B, of which LC A and B has no estimate of spatial accuracy. However, the accuracy order was LC A, B, and 0 in Hays et al. (2001) and LC A, 0, and B in Witt et al. (2010). However, some studies still have used LC 0 (Hart et al. 2012; Saito et al. 2015), and although replacing LC 0 data with LC A will make little difference to the overall interpretation, it appears that almost all analyses will have to be performed from scratch. Therefore, we mentioned the used LC 0 in this study, instead mentioned about cautionary note about location class selection. We added the sentences as shown below.

- Hart et al. 2012 Common coastal foraging areas for loggerhead in the Gulf of Mexico. Biological Conservation 145: 185-194.
- Saito et al. 2015. Tracking male loggerhead turtle migrations around Southwestern Japan using satellite telemetry. Chelonian Conservation and Biology. 14: 82-87.

⇨ Although LC 0, which has lower location quality, could overestimate home range size, the reduction in the number of location points due to the exclusion of LC 0 obscures the understanding the space use patterns of sea turtles (Thomson et al. 2017). Because the aim of this study was to focus on space use patterns in little-known areas rather than determining the detailed home range size of sea turtles, we included LC 0 for analysis. Additionally, reassessment of location accuracy is necessary considering studies showing that LC A has higher accuracy than LC 0 ([45,49]Hay et al. 2001; Witt et al. 2010).

- 45: Witt MJ, Åkesson S, Broderick AC, Coyne MS, Ellick J, Formia A, et al. Assessing accuracy and utility of satellite-tracking data using Argos-linked Fastloc-GPS. Anim Behav. 2010;80: 571. doi: 10.1016/j.anbehav2010.05.022.

- 49: Hays GC, Åkesson S, Godley BJ, Luschi P, Santidrian P. The implications of location accuracy for the interpretation of satellite-tracking data. Anim Behav. 2001;61: 1035-1040.

9. Line 113. " … we used the minimum convex polygon (MCP) and 95 and 50 percent volume contours (PVC) based on kernel density estimation.
You need to discuss that you likely over-estimate home-range using low quality Argos tracking, compared to if you has used high-resolution Fastloc-GPS tracking. See:
Thomson JA et al. (2017). Implications of location accuracy and data volume for home range estimation and

fine-scale movement analysis: comparing Argos and Fastloc-GPS tracking data. Marine Biology 164, 204. DOI 10.1007/s00227-017-3225-7
This is not necessarily a problem but you need to acknowledge this issue in the discussion with words like: "We appreciate that we likely overestimated space use by turtles by using Argos locations which have low accuracy compared to other approaches such as Fastloc-GPS (ref)".

A: We just focused on using the Argos transmitter we had already purchased. We are very grateful for the information about the much higher accuracy transmitter, Fastloc-GPS. If Fastloc-GPS had been used, we could get more detailed and reliable data. Therefore, we acknowledged the shortcomings of our results and added the following sentences.

⇨ The Argos transmitter has been widely used for satellite tracking of sea turtles until recently [12, 13, 19, 41], although it could overestimate space use of sea turtles compared to more accurate approaches such as Fastloc-GPS (Witt et al. 2010; Thomson et al. 2017).

- Thomson JA et al. (2017). Implications of location accuracy and data volume for home range estimation and fine-scale movement analysis: comparing Argos and Fastloc-GPS tracking data. Marine Biology 164, 204. DOI 10.1007/s00227-017-3225-7

- Witt MJ et al. (2010). Assessing accuracy and utility of satellite-tracking data using Argos- linked Fastloc-GPS. Animal Behaviour 80, 571-581. doi:10.1016/j.anbehav.2010.05.022

10. The discussion is rather parochial which will limit readership. I would try and consider some wider issues. I think you need to discuss whether these turtles were behaving normally. Usually adult turtles travel back to the same foraging site to which them maintain fidelity. See:
Shimada T et al. (2020). Fidelity to foraging sites after long migrations. Journal of Animal Ecology 89, 1008–1016. https://doi.org/10.1111/1365-2656.13157
Other have tracked rehabilitated turtles. Are they behaving normally?

A1: Between 30 and 50 stranded or bycatch sea turtles are rescued every year. National Marine Biodiversity Institute of Korea tracked physically and behaviorally healthy turtles among all rehabilitated sea turtles.

⇨ From October 2015 to October 2022, we tracked 15 rehabilitated sea turtles (7 loggerhead and 8 green turtles), which were caught incidentally and rescued from fishing nets near the coastal areas of the Republic of Korea (Table 1; S1 Table).

A2: Because sea turtle research in this area is very limited due to the relatively small number of turtles and small research history, there is not much comparable data on habitat use by sea turtles. In addition, two previous studies in this area were conducted over a relatively short period of time or about juveniles (Kim et al. 2022; Jang et al. 2018). Nevertheless, because sea turtles in this study showed the responses to the sea temperature and seasonal habitat use patterns of some turtles were identified, we judged that they had recovered well and behaved normally.

- Kim et al. 2022. Movement patterns of juvenile loggerhead turtles (*Caretta caretta* L. 1758) and green turtles (*Chelonia mydas* L. 1758) hatched in captivity and released in the Korean Waters. Animals. 2022;12: 2157.
- Jang S et al. 2018 Movements of green turtles (*Chelonia mydas*) rescued from pound nets near Jeju Island, Republic of Korea. Chelonian Conserv Biol. 17: 236-244.

⇨ The periodic behavior of tracked sea turtles and their response to environmental variables, especially SST, Chla, and POC, showed that the rescued turtles were rehabilitated well and are adapting to the sea. The information obtained through them expands our horizons on the travel patterns and habitat use of loggerhead and green turtles in the Northwest Pacific Ocean, the northern limit of their distribution range, where little is known about the spatial ecology of sea turtles.

11. Linked to point 7, I would specify where turtles were originally captured and how far away these sites were from the release points.

A: we added S1 Table to specify the rescue data and release data, including the distance from the rescue location to the release location.

12. I was asked to comment on the data availability. It is not clear to me where the raw tracking data can be accessed (time stamps lat/longs for each turtle). I suggest you deposit in Movebank or a similar data bank.

A: We uploaded our tracking data about 15 sea turtles on Movebank (study name: Marine bioresource conservation and restoration research, Sensor type: Radio Transmitter). The Current data access setting is “Public” at the Visibility of study and tracks and “Data manager only” at the Data download. If you need access to data, we can provide it personally.

https://www.movebank.org/movebank/#page=studies,path=study3030282201

Response to Reviewer 2

Thank you for the opportunity to review "Habitat use of loggerhead (Caretta caretta) and green (Chelonia mydas) turtles in the Northwest Pacific Ocean, the northern limit of their distribution range." This is an important study theme and having more telemetry data for the region will be useful for expanding our knowledge of the biology of both species, and also provide information for conservation action.

In reading the paper, I had started to do line-by-line suggested edits (see below), but I only provide such edits until the Methods section. This is because as I was reading the paper, I discovered that had several fundamental comments and concerns about the paper, and thus this review has largely focused on conceptual themes relating to movement analysis and home range estimation.

First, for loggerheads, the statement that this is at the northern limit of their distributional range should always be clarified that you are talking about the waters of the western Pacific near Japan. Indeed, 39.3N is a northern limit for green turtles, and loggerheads near the Japanese prefecture, but ss per Kobayashi et al. (2008, JEMBE) and others, loggerheads in the Central North Pacific swim to higher than 45N. Thus the title of this paper is confusing needs to be slightly adjusted after the ‘,’ to make it clear you are referring to the NWP only. The same is true for all other mentions of this throughout the paper.

A: We agree with your suggestion. Thus, we changed the title to “in the northern limit of their distribution range of the Northwest Pacific Ocean”. As well as in the main text.

Second, I have major concerns about the authors' application of the term Home Range. This term is usually applied to movement data for turtles that set up residency in localized areas and remain inside clear patial boundaries for long periods of time (e.g. green turtle benthic foraging in coastal habitats). However, for animals living in offshore waters, home range is not the best term, as turtles in this study they were more nomadic, and moved throughout a very large areas. Indeed, the home range size you mention of up to 181,342 ha, is not reflective of what the term ‘home range’ is typically intended to describe. This is so because for the study turtles, when they stop to feed, the food patch does not stay in the same place all the time, thus turtles are not really inhabiting a fixed home range, but rather an ephemeral, spatially dynamic, food rich area. I encourage the authors to think about this and propose a different, perhaps novel term for these ‘high seas foraging ranges’. Adding such a term would be a valuable contribution to the science of sea turtle movement analysis.

A: We understand that MCP and 95 PVC could overestimate the home range of sea turtles as you indicated. After release, the rehabilitated turtles might have taken a lot of effort to adapt to the sea and wandered to find suitable habitats. For that reason, we focused on 50 PVC, which are generally defined as core habitat, although we presented MCP and 95 PVCs as the home range. Additionally, ‘high seas foraging ranges’ is a novel term, while the spatial range of sea turtles determined by MCP and 95 PVC is commonly mentioned as ‘home range’. We are concerned that using the new term may cause readers’ unacceptance or disturb their understanding. Therefore, we added caution against overestimating the home range in the Method section.

- ⇨ The home range of sea turtles could be overestimated considering the adaptation to the sea and migration distances to suitable habitats caused by their rehabilitation period in artificial facilities. Nevertheless, the periodic behavior of tracked sea turtles and their response to environmental variables, especially SST, Chla, and POC, showed that the rescued turtles were rehabilitated well and are adapting to the sea.

Third, relating to the above, for satellite tracked turtles, the home range (or in your case the high seas foraging range) of a turtle is only a subset of the entire track duration, except for when turtles are equipped with satellite transmitters after being captured within their foraging habitat. Otherwise, such as the case with turtles moving in the open ocean or in offshore, non-coastal waters, any given turtle track will usually have a portion of the track that is 'directional movement' as the turtle moved from point A to point B, and another portion of the track where they encounter food and slow their movements to forage in those localities.

Often researchers use turning angles or travel speed filters to depict when a turtle has slowed down and reached its foraging area; the track portion that occurs outside of these foraging areas is not part of its home range or foraging range, but rather a transitional area, or movement area/corridor.

In this paper, it is unclear in the methods which portion of the tracks were included in the home range analysis. Was it the entire track? Was it a subset of the track once the turtle slowed down? If the former, then inclusion of the entire track is not an accurate depiction of a home range or foraging range MCP. If you are going to include the entire track in the home range analysis then this approach needs to be thoroughly justified in the text.

Instead it appears to me that the 50% PVC is used to depict the core area of foraging. If using the PVC 50% area to determine ‘foraging range’, this needs to be justified, considering that the vast majority of spatial data analyses use some sort of data filter to determine the area on a track that includes foraging activity. With that said, if you can show that the daily movement speeds of turtles occupying the 50% PVC is significantly slower than turtles outside these areas, this would be compelling support of the use of 50% PVC areas for depicting areas of foraging for a turtle in offshore waters. That being said, I am highly skeptical of this approach because the 50% PVC--as in the case of KOR0149--can be high density points not because a turtle had stopped there and spent much time, but rather because it moved back and forth through an area, never slowing down, yet because the turtle crisscrossed the area, the satellite location density is high, and the area is calculated mathematically as a core area, when in fact it is simply the 'crossroads' where a turtle keeps passing through. For me, a true core area would be more like what is depicted for KOR0151. This is a very important consideration and this caveat needs to be presented in the text if keeping the 50%PVC approach.

A: 50 PVC is calculated based on the kernel density estimation. That is, a relatively high-density area of location data is included within a 50 PVC area. As you advised, it is not proper to simply define 50 PVC as a foraging area. Therefore, we will define a 50 PVC area as a foraging area only if the location data within 50 PVCs has a movement speed that is significantly lower than the outside, considering the slower movement speed of sea turtles in the foraging area. We statistically compared the daily distance traveled (DDT) for 50 PVC and outside points. The related information is presented in S2 Table.

- ⇨ We compared the DDT of location data inside and outside 50 PVC, and defined the 50 PVC as a foraging area if the DDT inside was significantly smaller, based on the slow travel speed of sea turtles in their foraging areas (Zbinden et al. 2008; Schofield et al. 2010). On the other hand, we defined all areas outside 50 PVC and where DDT was not significantly small as transitional areas.
- ⇨ To distinguish between the foraging or nesting area and the transitional area, we used an independent t-test, a one-way ANOVA test, and the Kruskal-Wallis test. First, we conducted the Shapiro-Wilkinson test with DDT value of each sea turtle for normality. When only one 50 PVC was generated in a sea turtle individual, the inside and outside DDT were compared using a t-test. If a sea turtle has multiple 50 PVCs, we conducted one-way ANOVA or Kruskal-Wallis test on the inside and outside of each 50 PVC. Whenever significant differences were detected, we defined only 50 PVC that had significantly small DDT compared to the outside as the foraging or nesting area by the Bonferroni or Dunn post hoc test.

A: Applying the new method, differences from previous results were found only in loggerhead turtles and not in green turtles. One 50 PVC of KOR0091 was newly determined as a foraging area and 50 PVC of KOR0149,

which was previously a foraging area, was excluded, as you guessed. Fig 2 was also modified to reflect the new result.

Fourth, and relating to PVC evaluations, the authors note that “We defined the 50 PVC as the area where turtles stayed for more than 15 days while foraging.”. Why was 15 days picked as a threshold? This seems arbitrary. It is quite possible that, due to the ephemeral nature of high seas food patches, that turtles could have encountered such areas an foraged for less than 15 days before moving to the next patch. Have the authors considered this? A much more robust technique would be to implement some sort of movement speed or turning angle filter to depict where on the movement path did turtles slow down, turn more, and likely forage.

A: We acknowledge that it is not objective to exclude 50 PVC areas turtles used for less than 15 days from foraging areas. Therefore, as explained above, if the daily distance traveled by turtles in an area within 50 PVC was significantly smaller than in areas outside, we determined that area as a foraging area.

Fifth, for the analysis of oceanographic variables, based on the paragraph starting on line 124, it is unclear if oceanography was measure for the entire track, or just the 50%PVC locations as is suggested in the following paragraph. If the latter, then this would be a great opportunity to further support the use of the 50% PVC area to depict foraging by comparing oceanographic variables in the 50% area vs. the remainder of the tracks.

A: We presented the related results in Table 3, distinguished between those included within the foraging area (50 PVCs) and those not. Also, we statistically compared the differences in environmental variables between foraging areas and traveling areas and presented them in Table 4. However, the value was modified by redefining the foraging area, according to the third question.

These are my main concerns with the paper that need to be addressed before this manuscript is acceptable for publication. I hope the authors find these comments constructive, and I do believe that the information in this study is highly publishable, as soon as the authors correctly frame what the spatial parameters are that they are analyzing.

In addition, here area few inline text edits that may be helpful.

46 – change ‘since’ to ‘because’

A: We replaced ‘since’ with ‘because’.

53 – insert ‘studying’ after ‘for’

A: We added ‘for’.

61-63 – it is unnecessary to list all the species of sea turtles in Japan, as this not relevant to this paper. Instead start with a statement that is true for both greens and loggerheads foraging and nesting in the region.

A: We removed the unnecessary mention of five turtles and modified it as flows. ‘In the Northwest Pacific Ocean, Loggerhead (*Caretta caretta*) and green (*Chelonia mydas*) turtles are the most commonly observed sea turtles and use it as foraging and nesting regions’

67 – change ‘on the other hand’ to ‘nevertheless’, which is a more appropriate term here and not common jargon.

A: We replaced ‘on the other hand’ with ‘nevertheless’.

70 – this line should read: “increased observation of loggerheads and green turtles at these distribution margins, studies on their habitat use are lacking.”

A: We revised it.

77 – add ‘subadult and adult’ in front of turtles names. This will build off their mention in the previous paragraph.

A: We added ‘subadult and adult’ in front of turtles names.

79 – this line should read: “provide essential information about their spatial biology, which can be useful…”

A: We revised the sentence: “this study is expected to provide essential information about their spatial biology, which can be useful for the effective management…”

88 – this line should read: “identified as adults, and three turtles as potential…”

A: We revised the sentence: “12 turtles were identified as adults, and three turtles as potential subadults.”

92 – give range in SST after mentioning temperature.

A: We added it.

93 – for methods of attachment please describe the carapace cleaning and sanding preparation process

A: We added it.

98 – last day ‘of’

A: We added ‘of’

103 – you should also mention which LCs were included specifically after “the high-accuracy data”

A: We revised the location accuracy we used and added an additional explanation as to why we used the relatively low LC 0. Additional explanation is as follows.

⇨ “Although LC 0, which has lower location quality, could overestimate home range size, the reduction in the number of location points due to the exclusion of LC 0 obscures the understanding the space use patterns of sea turtles [46]. Because the aim of this study was to focus on space use patterns in little-known areas rather than determining the detailed home range size of sea turtles, we included LC 0 for analysis. Additionally, reassessment of location accuracy is necessary considering studies showing that LC A has higher accuracy than LC 0 [45,49].”

- 46: Thomson JA et al. (2017). Implications of location accuracy and data volume for home range estimation and fine-scale movement analysis: comparing Argos and Fastloc-GPS tracking data. Marine Biology 164, 204.

- 45: Hays GC et al. (2001). The implications of location accuracy for the interpretation of satellite tracking data. Animal Behaviour 61, 1035-1040.

- 49: Witt MJ et al. (2010). Assessing accuracy and utility of satellite-tracking data using Argos- linked Fastloc-GPS. Animal Behaviour 80, 571-581.

156 – should be “with movements ranging from [lat long coordinates]

A: We revised the sentence.

Fig 1 legend, add “ ,respectively “ after “end points”

A: We added them.

Fig 1 – very hard to see the release circles. Can these be made bigger? Also, each map should be labeled loggerhead and green turtle at top of respective maps to make it easier to interpret the figure. Also OK to have in caption.

A: We have expanded the release points. Also, the species name was added to the upper left corner of the figure.
