# Supplementary material for: Habitat use of loggerhead (Caretta caretta) and green (Chelonia mydas) turtles at the northern limit of their distribution range of the Northwest Pacific Ocean
Source: PLoS One. 2024 Apr 4;19(4):e0290202. doi: 10.1371/journal.pone.0290202 (PMC10994308; doi:10.1371/journal.pone.0290202)
Supplement: Attachment — Submitted filename: Response letter.docx [file pone.0290202.s004.docx]

**Dear Section Editor of PLOS ONE,**

We appreciate your valuable edits and suggestions on our manuscript. Most comments were reasonable and made our research more rigorous and valuable. We have considered all of the reviewers’ comments and have revised the manuscript accordingly. All our edits can be viewed in the “Track Changes”. The response answers to the reviewers’ comments are written in blue text below.

**Response to Editor**

When submitting your revision, we need you to address these additional requirements.

1. Please ensure that your manuscript meets PLOS ONE's style requirements, including those for file naming. The PLOS ONE style templates can be found at 
https://journals.plos.org/plosone/s/file?id=wjVg/PLOSOne_formatting_sample_main_body.pdf and 
https://journals.plos.org/plosone/s/file?id=ba62/PLOSOne_formatting_sample_title_authors_affiliations.pdf

A. We wrote the manuscript considering the author guideline and above details.

2. Thank you for stating the following financial disclosure: 
"This study was supported by grants from the National Marine Biodiversity Institute of Korea (2023M00300 and 2023E00300) and the ‘Development of Technology for Impact Assessment of Marine Plastic Debris on Marine Ecosystem (EA0114)" funded by the Korea Institute of Ocean Science and Technology."

Please state what role the funders took in the study. If the funders had no role, please state: "The funders had no role in study design, data collection and analysis, decision to publish, or preparation of the manuscript." 
If this statement is not correct you must amend it as needed. 
Please include this amended Role of Funder statement in your cover letter; we will change the online submission form on your behalf.

A. The three directors who secured the Funders participated in the project administration, supervision and investigation. They contributed to the secure and release of sea turtles and data management.

3. PLOS requires an ORCID iD for the corresponding author in Editorial Manager on papers submitted after December 6th, 2016. Please ensure that you have an ORCID iD and that it is validated in Editorial Manager. To do this, go to ‘Update my Information’ (in the upper left-hand corner of the main menu), and click on the Fetch/Validate link next to the ORCID field. This will take you to the ORCID site and allow you to create a new iD or authenticate a pre-existing iD in Editorial Manager. Please see the following video for instructions on linking an ORCID iD to your Editorial Manager account: <https://www.youtube.com/watch?v=_xcclfuvtxQ>

A. Yes, corresponding author (An YR: 0000-0002-0218-6254) has joined ORCID and added information.

4. Please include your full ethics statement in the ‘Methods’ section of your manuscript file. In your statement, please include the full name of the IRB or ethics committee who approved or waived your study, as well as whether or not you obtained informed written or verbal consent. If consent was waived for your study, please include this information in your statement as well.

A. We've added it into the manuscript.

=> Permissions for the sea turtle conservation program were given by the Marine Animal Protection Committee of the Ministry of Oceans and Fisheries with whom this work was conducted.

5. We note that [Figures 1,2,3,5, and 6] in your submission contain [map/satellite] images which may be copyrighted. All PLOS content is published under the Creative Commons Attribution License (CC BY 4.0), which means that the manuscript, images, and Supporting Information files will be freely available online, and any third party is permitted to access, download, copy, distribute, and use these materials in any way, even commercially, with proper attribution. For these reasons, we cannot publish previously copyrighted maps or satellite images created using proprietary data, such as Google software (Google Maps, Street View, and Earth). For more information, see our copyright guidelines: <http://journals.plos.org/plosone/s/licenses-and-copyright>.

- PLOS ONE: Edits requested on your submission PONE-D-23-24778R1 - [EMID:645d75582986089b]

A. We made Figures 1,2,3,5, and 6 based on GADM (Database of Global Administrative Areas; <https://gadm.org/>) data. This data could be used for academic research, including PLos journal. We attached the licence from the GADM website (<https://gadm.org/license.html>).

6. Thank you for updating your data availability statement. You note that your data are available within the Supporting Information files, but no such files have been included with your submission. At this time we ask that you please upload your minimal data set as a Supporting Information file, or to a public repository such as Figshare or Dryad. Please also ensure that when you upload your file you include separate captions for your supplementary files at the end of your manuscript. As soon as you confirm the location of the data underlying your findings, we will be able to proceed with the review of your submission.

- PLOS ONE: Edits requested on your submission PONE-D-23-24778R1 - [EMID:645d75582986089b]

A. We have uploaded the sea turtle migration data to movebank as requested. We will compress the relevant files into a ZIP file and submit it to the Supporting Information - Compressed/ZIP File Archive section.

https://www.movebank.org/movebank/#page=studies,path=study3030282201

**Response to Reviewer 1**

1. Line 42. I think you need to be a little more honest and say that in many area turtle numbers are increasing, thanks to conservation efforts and so in the latest IUCN regional assessments not all populations are endangered. E.g. see: Mazaris AD et al. (2017). Global sea turtle conservation successes. Science Advances 3: e1600730. https://doi.org/10.1126/sciadv.1600730
If you look on the IUCN website at the latest regional assessments, you will see that in some areas turtles are not listed as endangered.

A: As your advice, we changed the first sentence in Introduction. The IUCN assessment criteria is separated into three threatened grades: Vulnerable, Endangered, and Critically Endangered. Therefore, we changed “endangered” to “threatened”. => “Although many regional sea turtle populations are increasing thanks to conservation efforts [1,2], still six out of all seven known sea turtle species are threatened worldwide …”

-1: Hudgins JA, Hudgins EJ, Köhnk S, Mohamed Riyad E, Stelfox MR. A brighter future? Stable and growing sea turtle populations in the Republic of Maldives. PLoS One 2023;18: e0283973. doi: 10.1371/journal.pone.0283973

- 2: Mazaris AD, Schofield G, Gkazinou C, Almpanidou V, Hays GC. Global sea turtle conservation successes. Sci Adv. 2017;3: e1600730. doi: 10.1126/sciadv.1600730.

2. Line 49. “expanding north”. I think you mean “polewards”. In the southern hemisphere species are heading south.

A: we agreed it and revised the sentence.

3. Line 51. “telemetry is a useful method for investigating the spatial ecology and habitat use of sea turtles …” I would simply cite a recent review: “Satellite tracking sea turtles: opportunities and challenges to address key questions. <https://doi.org/10.3389/fmars.2018.00432>”

A: Because the recommended paper supports this content properly, we cited this paper.

4. Lines 59. “Overall, the habitat information and environmental characteristics identified by satellite telemetry could contribute to the effective protection and restoration of sea turtle populations …”
Perhaps the best synthesis of how satellite tracking has helped drive turtle conservation is within this review: “Translating marine animal tracking data into conservation policy and management. Trends in Ecology and Evolution 34, 459-473. <https://doi.org/10.1016/j.tree.2019.01.009>”

A: Because the recommended paper supports this content properly, we cited this paper.

5. Line 86. “Considering the minimum CCL of sexually mature sea turtles is known as 73 cm in loggerhead turtles and 67 cm in green turtles …”

One of your green turtles was 96cm. I find it hard to believe there could be such a massive range of adult sizes from 67-96cm. I suggest you discuss whether any of these turtles were actually sub-adults.

A: In many studies, the criteria for determining whether a sea turtle is an adult or subadult are very diverse. In Green (1993), green turtles with a CCL of 67 cm or more were classified as adults because they could breed. Additionally, the growth rate of green turtles tends to decrease rapidly from a CCL of 65 cm (Limpus and Chaloupka 1997). We categorized growth (adult or subadult) to determine whether the individual used the habitat as a foraging or nesting site.

- Green D. 1993. Growth rates of wild immature green turtles in the Galapagos Islands, Ecuador. J Herpetol. 27: 338-341.
- Limpus C & Chaloupka M. 1997. Nonparametric regression modelling of green sea turtle growth rates (southern Great Barrier Reef). Mar Ecol Prog Ser. 149: 23-34.

6. Normally green turtles feed on seagrass or macroalgae. There are recent reviews on their diet (https://doi.org/10.1007/s00227-020-03786-8). In this context I would discuss how many of the tracked turtles foraged in shallow coastal areas versus being in deep oceanic areas.

A: We estimated feeding sites based on sea turtles' migration patterns and environmental characteristics, but since we did not conduct research directly related to feeding, we believe it is difficult to definitively determine feeding sites. Instead, since there was previous research on the food items of green turtles near South Korea, we added related content based on the research you provided.

- Additionally, green turtles consume animal matter more than seagrass at high latitudes or low SST areas under 25°C [67]. However, although green turtles generally used areas below SST of 23°C in our study, the most abundant food item was seaweed according to previous research about stomach contents [34]. This supports that green turtles use waters off South Korea as foraging areas in mainly warm season and travel to lower latitudes in the cold season.

-67: Esteban N, Mortimer JA, Stokes HJ, Laloë JO, Unsworth RK, Hays GC. A global review of green turtle diet: sea surface temperature as a potential driver of omnivory levels. Mar Biol 2020;167: 1-17. Doi: 10.1007/s00227-020-03786-8.

-34: Kim IH, Moon DY, Cho IY, Kim MS, An YR, Han D, et al. Occurrence of sea turtles in the Korean Waters and the morphological characteristics of two major species. Korean J Fish Aquat Sci. 2017;50: 311-318. Doi: 10.5657/KFAS.2017.0311.

7. Table 3. You talk about “travelling” and “foraging”. How did you distinguish these 2 different states?

A: We determined the foraging and nesting or transitional (previous travelling) areas using 50 PVC and speed difference. First, 50 PVC indicate a relatively high-density area. After that, we will define a 50 PVC area as a foraging area only if the location data within 50 PVCs has a movement speed that is significantly lower than the outside, considering the slower movement speed of sea turtles in the foraging area. We statistically compared the daily distance traveled (DDT) for 50 PVC and outside points. The related information is presented in S2 Table.

- We compared the DDT of location data inside and outside 50 PVC, and defined the 50 PVC as a foraging area if the DDT inside was significantly smaller, based on the slow travel speed of sea turtles in their foraging areas [51,52]. On the other hand, we defined all areas outside 50 PVC and where DDT was not significantly small as transitional areas.
- To distinguish between the foraging or nesting area and the transitional area, we used an independent t-test, a one-way ANOVA test, and the Kruskal-Wallis test. First, we conducted the Shapiro-Wilkinson test with DDT value of each sea turtle for normality. When only one 50 PVC was generated in a sea turtle individual, the inside and outside DDT were compared using a t-test. If a sea turtle has multiple 50 PVCs, we conducted one-way ANOVA or Kruskal-Wallis test on the inside and outside of each 50 PVC. Whenever significant differences were detected, we defined only 50 PVC that had significantly small DDT compared to the outside as the foraging or nesting area by the Bonferroni or Dunn post hoc test.

- 51: Zbinden JA, Aebischer A, Margaritoulis D, Arlettaz R. Important areas at sea for adult loggerhead sea turtles in the Mediterranean Sea: satellite tracking corroborates findings from potentially biased sources. Mar Biol. 2008;153: 899-906. doi: 10.1007/s00227-007-0862-2.

- 52: Schofield G, Hobson VJ, Fossette S, Lilley MK, Katselidis KA, Hays GC. Fidelity to foraging sites, consistency of migration routes and habitat modulation of home range by sea turtles. Divers Distrib. 2010;16: 840-853. doi: 10.1111/j.1472-4642.2010.00694.x.

8. Lines 102. “… classes A and B, with no estimated accuracy”.

This is not true.
Class A and B accuracy has been assessed in various studies. See for example:

Witt MJ et al. (2010). Assessing accuracy and utility of satellite-tracking data using Argos-linked Fastloc-GPS. Animal Behaviour 80, 571-581. doi:10.1016/j.anbehav.2010.05.022
Hays GC et al. (2001). The implications of location accuracy for the interpretation of satellite tracking data. Animal Behaviour 61, 1035-1040.

These studies show that Class A locations are good.
The worst accuracy is class 0 locations.
So I think you need to omit class 0 locations and include class A locations.
Also note that now Argos can provide Kalman filtered locations. The users decides. So specify whether you used Kalman filtered locations or not.

A: Generally, the order of accuracy is known to be LC 0, A, and B, of which LC A and B has no estimate of spatial accuracy. However, the accuracy order was LC A, B, and 0 in Hays et al. (2001) and LC A, 0, and B in Witt et al. (2010). However, some studies still have used LC 0 (Hart et al. 2012; Saito et al. 2015), and although replacing LC 0 data with LC A will make little difference to the overall interpretation, it appears that almost all analyses will have to be performed from scratch. Therefore, we mentioned the used LC 0 in this study, instead mentioned about cautionary note about location class selection. We added the sentences as shown below.

- Hart et al. 2012 Common coastal foraging areas for loggerhead in the Gulf of Mexico. Biological Conservation 145: 185-194.
- Saito et al. 2015. Tracking male loggerhead turtle migrations around Southwestern Japan using satellite telemetry. Chelonian Conservation and Biology. 14: 82-87.
- Although LC 0, which has lower location quality, could overestimate home range size, the reduction in the number of location points due to the exclusion of LC 0 obscures the understanding the space use patterns of sea turtles (Thomson et al. 2017). Because the aim of this study was to focus on space use patterns in little-known areas rather than determining the detailed home range size of sea turtles, we included LC 0 for analysis. Additionally, reassessment of location accuracy is necessary considering studies showing that LC A has higher accuracy than LC 0 ([45,49]Hay et al. 2001; Witt et al. 2010).

- 45: Witt MJ, Åkesson S, Broderick AC, Coyne MS, Ellick J, Formia A, et al. Assessing accuracy and utility of satellite-tracking data using Argos-linked Fastloc-GPS. Anim Behav. 2010;80: 571. doi: 10.1016/j.anbehav2010.05.022.

- 49: Hays GC, Åkesson S, Godley BJ, Luschi P, Santidrian P. The implications of location accuracy for the interpretation of satellite-tracking data. Anim Behav. 2001;61: 1035-1040.

9. Line 113. “ … we used the minimum convex polygon (MCP) and 95 and 50 percent volume contours (PVC) based on kernel density estimation.
You need to discuss that you likely over-estimate home-range using low quality Argos tracking, compared to if you has used high-resolution Fastloc-GPS tracking. See:
Thomson JA et al. (2017). Implications of location accuracy and data volume for home range estimation and fine-scale movement analysis: comparing Argos and Fastloc-GPS tracking data. Marine Biology 164, 204. DOI 10.1007/s00227-017-3225-7
This is not necessarily a problem but you need to acknowledge this issue in the discussion with words like: “We appreciate that we likely overestimated space use by turtles by using Argos locations which have low accuracy compared to other approaches such as Fastloc-GPS (ref)”.

A: We just focused on using the Argos transmitter we had already purchased. We are very grateful for the information about the much higher accuracy transmitter, Fastloc-GPS. If Fastloc-GPS had been used, we could get more detailed and reliable data. Therefore, we acknowledged the shortcomings of our results and added the following sentences.

- The Argos transmitter has been widely used for satellite tracking of sea turtles until recently [12, 13, 19, 41], although it could overestimate space use of sea turtles compared to more accurate approaches such as Fastloc-GPS (Witt et al. 2010; Thomson et al. 2017).

- Thomson JA et al. (2017). Implications of location accuracy and data volume for home range estimation and fine-scale movement analysis: comparing Argos and Fastloc-GPS tracking data. Marine Biology 164, 204. DOI 10.1007/s00227-017-3225-7

- Witt MJ et al. (2010). Assessing accuracy and utility of satellite-tracking data using Argos- linked Fastloc-GPS. Animal Behaviour 80, 571-581. doi:10.1016/j.anbehav.2010.05.022

10. The discussion is rather parochial which will limit readership. I would try and consider some wider issues. I think you need to discuss whether these turtles were behaving normally. Usually adult turtles travel back to the same foraging site to which them maintain fidelity. See:
Shimada T et al. (2020). Fidelity to foraging sites after long migrations. Journal of Animal Ecology 89, 1008–1016. https://doi.org/10.1111/1365-2656.13157
Other have tracked rehabilitated turtles. Are they behaving normally?

A1: Between 30 and 50 stranded or bycatch sea turtles are rescued every year. National Marine Biodiversity Institute of Korea tracked physically and behaviorally healthy turtles among all rehabilitated sea turtles.

- From October 2015 to October 2022, we tracked 15 rehabilitated sea turtles (7 loggerhead and 8 green turtles), which were caught incidentally and rescued from fishing nets near the coastal areas of the Republic of Korea (Table 1; S1 Table).

A2: Because sea turtle research in this area is very limited due to the relatively small number of turtles and small research history, there is not much comparable data on habitat use by sea turtles. In addition, two previous studies in this area were conducted over a relatively short period of time or about juveniles (Kim et al. 2022; Jang et al. 2018). Nevertheless, because sea turtles in this study showed the responses to the sea temperature and seasonal habitat use patterns of some turtles were identified, we judged that they had recovered well and behaved normally.

- Kim et al. 2022. Movement patterns of juvenile loggerhead turtles (*Caretta caretta* L. 1758) and green turtles (*Chelonia mydas* L. 1758) hatched in captivity and released in the Korean Waters. Animals. 2022;12: 2157.
- Jang S et al. 2018 Movements of green turtles (*Chelonia mydas*) rescued from pound nets near Jeju Island, Republic of Korea. Chelonian Conserv Biol. 17: 236-244.
- The periodic behavior of tracked sea turtles and their response to environmental variables, especially SST, Chla, and POC, showed that the rescued turtles were rehabilitated well and are adapting to the sea. The information obtained through them expands our horizons on the travel patterns and habitat use of loggerhead and green turtles in the Northwest Pacific Ocean, the northern limit of their distribution range, where little is known about the spatial ecology of sea turtles.

11. Linked to point 7, I would specify where turtles were originally captured and how far away these sites were from the release points.

A: we added S1 Table to specify the rescue data and release data, including the distance from the rescue location to the release location.

12. I was asked to comment on the data availability. It is not clear to me where the raw tracking data can be accessed (time stamps lat/longs for each turtle). I suggest you deposit in Movebank or a similar data bank.

A: We uploaded our tracking data about 15 sea turtles on Movebank (study name: Marine bioresource conservation and restoration research, Sensor type: Radio Transmitter). The Current data access setting is “Public” at the Visibility of study and tracks and “Data manager only” at the Data download. If you need access to data, we can provide it personally.

https://www.movebank.org/movebank/#page=studies,path=study3030282201

**Response to Reviewer 2**

Thank you for the opportunity to review "Habitat use of loggerhead (Caretta caretta) and green (Chelonia mydas) turtles in the Northwest Pacific Ocean, the northern limit of their distribution range." This is an important study theme and having more telemetry data for the region will be useful for expanding our knowledge of the biology of both species, and also provide information for conservation action.

In reading the paper, I had started to do line-by-line suggested edits (see below), but I only provide such edits until the Methods section. This is because as I was reading the paper, I discovered that had several fundamental comments and concerns about the paper, and thus this review has largely focused on conceptual themes relating to movement analysis and home range estimation.

First, for loggerheads, the statement that this is at the northern limit of their distributional range should always be clarified that you are talking about the waters of the western Pacific near Japan. Indeed, 39.3N is a northern limit for green turtles, and loggerheads near the Japanese prefecture, but ss per Kobayashi et al. (2008, JEMBE) and others, loggerheads in the Central North Pacific swim to higher than 45N. Thus the title of this paper is confusing needs to be slightly adjusted after the ‘,’ to make it clear you are referring to the NWP only. The same is true for all other mentions of this throughout the paper.

A: We agree with your suggestion. Thus, we changed the title to “in the northern limit of their distribution range of the Northwest Pacific Ocean”. As well as in the main text.

Second, I have major concerns about the authors' application of the term Home Range. This term is usually applied to movement data for turtles that set up residency in localized areas and remain inside clear patial boundaries for long periods of time (e.g. green turtle benthic foraging in coastal habitats). However, for animals living in offshore waters, home range is not the best term, as turtles in this study they were more nomadic, and moved throughout a very large areas. Indeed, the home range size you mention of up to 181,342 ha, is not reflective of what the term ‘home range’ is typically intended to describe. This is so because for the study turtles, when they stop to feed, the food patch does not stay in the same place all the time, thus turtles are not really inhabiting a fixed home range, but rather an ephemeral, spatially dynamic, food rich area. I encourage the authors to think about this and propose a different, perhaps novel term for these ‘high seas foraging ranges’. Adding such a term would be a valuable contribution to the science of sea turtle movement analysis.

A: We understand that MCP and 95 PVC could overestimate the home range of sea turtles as you indicated. After release, the rehabilitated turtles might have taken a lot of effort to adapt to the sea and wandered to find suitable habitats. For that reason, we focused on 50 PVC, which are generally defined as core habitat, although we presented MCP and 95 PVCs as the home range. Additionally, ‘high seas foraging ranges’ is a novel term, while the spatial range of sea turtles determined by MCP and 95 PVC is commonly mentioned as ‘home range’. We are concerned that using the new term may cause readers’ unacceptance or disturb their understanding. Therefore, we added caution against overestimating the home range in the Method section.

- The home range of sea turtles could be overestimated considering the adaptation to the sea and migration distances to suitable habitats caused by their rehabilitation period in artificial facilities. Nevertheless, the periodic behavior of tracked sea turtles and their response to environmental variables, especially SST, Chla, and POC, showed that the rescued turtles were rehabilitated well and are adapting to the sea.

Third, relating to the above, for satellite tracked turtles, the home range (or in your case the high seas foraging range) of a turtle is only a subset of the entire track duration, except for when turtles are equipped with satellite transmitters after being captured within their foraging habitat. Otherwise, such as the case with turtles moving in the open ocean or in offshore, non-coastal waters, any given turtle track will usually have a portion of the track that is 'directional movement' as the turtle moved from point A to point B, and another portion of the track where they encounter food and slow their movements to forage in those localities.

Often researchers use turning angles or travel speed filters to depict when a turtle has slowed down and reached its foraging area; the track portion that occurs outside of these foraging areas is not part of its home range or foraging range, but rather a transitional area, or movement area/corridor.

In this paper, it is unclear in the methods which portion of the tracks were included in the home range analysis. Was it the entire track? Was it a subset of the track once the turtle slowed down? If the former, then inclusion of the entire track is not an accurate depiction of a home range or foraging range MCP. If you are going to include the entire track in the home range analysis then this approach needs to be thoroughly justified in the text.

Instead it appears to me that the 50% PVC is used to depict the core area of foraging. If using the PVC 50% area to determine ‘foraging range’, this needs to be justified, considering that the vast majority of spatial data analyses use some sort of data filter to determine the area on a track that includes foraging activity. With that said, if you can show that the daily movement speeds of turtles occupying the 50% PVC is significantly slower than turtles outside these areas, this would be compelling support of the use of 50% PVC areas for depicting areas of foraging for a turtle in offshore waters. That being said, I am highly skeptical of this approach because the 50% PVC--as in the case of KOR0149--can be high density points not because a turtle had stopped there and spent much time, but rather because it moved back and forth through an area, never slowing down, yet because the turtle crisscrossed the area, the satellite location density is high, and the area is calculated mathematically as a core area, when in fact it is simply the 'crossroads' where a turtle keeps passing through. For me, a true core area would be more like what is depicted for KOR0151. This is a very important consideration and this caveat needs to be presented in the text if keeping the 50%PVC approach.

A: 50 PVC is calculated based on the kernel density estimation. That is, a relatively high-density area of location data is included within a 50 PVC area. As you advised, it is not proper to simply define 50 PVC as a foraging area. Therefore, we will define a 50 PVC area as a foraging area only if the location data within 50 PVCs has a movement speed that is significantly lower than the outside, considering the slower movement speed of sea turtles in the foraging area. We statistically compared the daily distance traveled (DDT) for 50 PVC and outside points. The related information is presented in S2 Table.

- We compared the DDT of location data inside and outside 50 PVC, and defined the 50 PVC as a foraging area if the DDT inside was significantly smaller, based on the slow travel speed of sea turtles in their foraging areas (Zbinden et al. 2008; Schofield et al. 2010). On the other hand, we defined all areas outside 50 PVC and where DDT was not significantly small as transitional areas.
- To distinguish between the foraging or nesting area and the transitional area, we used an independent t-test, a one-way ANOVA test, and the Kruskal-Wallis test. First, we conducted the Shapiro-Wilkinson test with DDT value of each sea turtle for normality. When only one 50 PVC was generated in a sea turtle individual, the inside and outside DDT were compared using a t-test. If a sea turtle has multiple 50 PVCs, we conducted one-way ANOVA or Kruskal-Wallis test on the inside and outside of each 50 PVC. Whenever significant differences were detected, we defined only 50 PVC that had significantly small DDT compared to the outside as the foraging or nesting area by the Bonferroni or Dunn post hoc test.

A: Applying the new method, differences from previous results were found only in loggerhead turtles and not in green turtles. One 50 PVC of KOR0091 was newly determined as a foraging area and 50 PVC of KOR0149, which was previously a foraging area, was excluded, as you guessed. Fig 2 was also modified to reflect the new result.

Fourth, and relating to PVC evaluations, the authors note that “We defined the 50 PVC as the area where turtles stayed for more than 15 days while foraging.”. Why was 15 days picked as a threshold? This seems arbitrary. It is quite possible that, due to the ephemeral nature of high seas food patches, that turtles could have encountered such areas an foraged for less than 15 days before moving to the next patch. Have the authors considered this? A much more robust technique would be to implement some sort of movement speed or turning angle filter to depict where on the movement path did turtles slow down, turn more, and likely forage.

A: We acknowledge that it is not objective to exclude 50 PVC areas turtles used for less than 15 days from foraging areas. Therefore, as explained above, if the daily distance traveled by turtles in an area within 50 PVC was significantly smaller than in areas outside, we determined that area as a foraging area.

Fifth, for the analysis of oceanographic variables, based on the paragraph starting on line 124, it is unclear if oceanography was measure for the entire track, or just the 50%PVC locations as is suggested in the following paragraph. If the latter, then this would be a great opportunity to further support the use of the 50% PVC area to depict foraging by comparing oceanographic variables in the 50% area vs. the remainder of the tracks.

A: We presented the related results in Table 3, distinguished between those included within the foraging area (50 PVCs) and those not. Also, we statistically compared the differences in environmental variables between foraging areas and traveling areas and presented them in Table 4. However, the value was modified by redefining the foraging area, according to the third question.

These are my main concerns with the paper that need to be addressed before this manuscript is acceptable for publication. I hope the authors find these comments constructive, and I do believe that the information in this study is highly publishable, as soon as the authors correctly frame what the spatial parameters are that they are analyzing.

In addition, here area few inline text edits that may be helpful.

46 – change ‘since’ to ‘because’

A: We replaced ‘since’ with ‘because’.

53 – insert ‘studying’ after ‘for’

A: We added ‘for’.

61-63 – it is unnecessary to list all the species of sea turtles in Japan, as this not relevant to this paper. Instead start with a statement that is true for both greens and loggerheads foraging and nesting in the region.

A: We removed the unnecessary mention of five turtles and modified it as flows. ‘In the Northwest Pacific Ocean, Loggerhead (Caretta caretta) and green (Chelonia mydas) turtles are the most commonly observed sea turtles and use it as foraging and nesting regions’

67 – change ‘on the other hand’ to ‘nevertheless’, which is a more appropriate term here and not common jargon.

A: We replaced ‘on the other hand’ with ‘nevertheless’.

70 – this line should read: “increased observation of loggerheads and green turtles at these distribution margins, studies on their habitat use are lacking.”

A: We revised it.

77 – add ‘subadult and adult’ in front of turtles names. This will build off their mention in the previous paragraph.

A: We added ‘subadult and adult’ in front of turtles names.

79 – this line should read: “provide essential information about their spatial biology, which can be useful…”

A: We revised the sentence: “this study is expected to provide essential information about their spatial biology, which can be useful for the effective management…”

88 – this line should read: “identified as adults, and three turtles as potential…”

A: We revised the sentence: “12 turtles were identified as adults, and three turtles as potential subadults.”

92 – give range in SST after mentioning temperature.

A: We added it.

93 – for methods of attachment please describe the carapace cleaning and sanding preparation process

A: We added it.

98 – last day ‘of’

A: We added ‘of’

103 – you should also mention which LCs were included specifically after “the high-accuracy data”

A: We revised the location accuracy we used and added an additional explanation as to why we used the relatively low LC 0. Additional explanation is as follows.

- “Although LC 0, which has lower location quality, could overestimate home range size, the reduction in the number of location points due to the exclusion of LC 0 obscures the understanding the space use patterns of sea turtles [46]. Because the aim of this study was to focus on space use patterns in little-known areas rather than determining the detailed home range size of sea turtles, we included LC 0 for analysis. Additionally, reassessment of location accuracy is necessary considering studies showing that LC A has higher accuracy than LC 0 [45,49].”

- 46: Thomson JA et al. (2017). Implications of location accuracy and data volume for home range estimation and fine-scale movement analysis: comparing Argos and Fastloc-GPS tracking data. Marine Biology 164, 204.

- 45: Hays GC et al. (2001). The implications of location accuracy for the interpretation of satellite tracking data. Animal Behaviour 61, 1035-1040.

- 49: Witt MJ et al. (2010). Assessing accuracy and utility of satellite-tracking data using Argos- linked Fastloc-GPS. Animal Behaviour 80, 571-581.

156 – should be “with movements ranging from [lat long coordinates]

A: We revised the sentence.

Fig 1 legend, add “ ,respectively “ after “end points”

A: We added them.

Fig 1 – very hard to see the release circles. Can these be made bigger? Also, each map should be labeled loggerhead and green turtle at top of respective maps to make it easier to interpret the figure. Also OK to have in caption.

A: We have expanded the release points. Also, the species name was added to the upper left corner of the figure.
