# Supplementary material for: Habitat use of loggerhead (Caretta caretta) and green (Chelonia mydas) turtles at the northern limit of their distribution range of the Northwest Pacific Ocean
Source: PLoS One. 2024 Apr 4;19(4):e0290202. doi: 10.1371/journal.pone.0290202 (PMC10994308; doi:10.1371/journal.pone.0290202)
Supplement: Attachment — Submitted filename: Response letter_PONE-D-23-24778R1.docx [file pone.0290202.s006.docx]

**Dear Section Editor of PloS One**

We greatly appreciate your efforts in improving the quality of our manuscript. According to the suggestions, we revised it and all our edits can be viewed in the “track change” in the newly attached manuscript.

Q1. Please define DDT here on first use.

- A: We added the definition of DDT (daily distance traveled) in line 128. Also, the ‘daily distance traveled’ below has been unified as DDT.

Q2. Line2: Changed “in” to “at”

- A: We changed it.

Q3. Line 50: remove “north”

- A: We changed “polewardnorth” to “poleward”

Q4. Line 63: Should loggerhead be capitalized, but not green turtle?

- A: We changed “Loggerhead” to “loggerhead”.

Q5. Line 64: replace “and using it as foraging and nesting regions” with “using the region for foraging and nesting”.

- A: We replaced it.

Q6. Line 91: change “as” to “to be”.

- A: We changed it.

Q7. Line 93: Change ‘at the coasts” to “along the coast”.

- A: We changed it.

Q8. Line 101: Delete “the”.

- A: We deleted it.

Q9. Line 110: change "after the release date to the last day of if the transmitter signal is not observed for two months from the date of the last signal received" to: "following the release date until the last day of transmission, which was determined to be the last day of transmission following a period of two months when no transmissions were received."

- A: We replaced it.

Q10. Line 117: Insert “data in Classes A and B”.

- A: We inserted it.

Q11. Line 119: Is LC location class? Need to define if using as an abbreviation.

- A: We defined LC as “location class” in line 115 first. According, all classes were replaced with LC.

Q12. Line 164: Add “in order” to the beginning of this sentence.

- A: We added it.

Q13. Line 167: change “has” to “had”

- A: We changed it.

Q14. Line 169: This sentence isn’t clear to me.

- A: To improve the understanding it, we changed the sentence as follow (Line 167 – 170). => If a sea turtle had multiple 50 PVCs, we conducted one-way ANOVA with the Bonferroni post hoc test or Kruskal-Wallis test with the Dunn post hoc test on the inside and outside of each 50 PVC. We defined the inside of 50 PVC that turtles had significantly shorter DDT compared to the outside as the foraging or nesting area.

Q15. Line 190: please label the variance as standard deviation or standard error.

- A: We added the "results are presented as mean ± standard deviation unless otherwise stated" in the first paragraph of the Results section (Line 182).

Q16. Line 233: add “as follows” to the end of this sentence.

- A: We added it.

Q17. Line 236: As this relates to a single turtle, change “areas” to “area” and “were” to “was”.

- A: We changed it.

Q18. Line 321: is it correct to say that Okinawa and Jeju Island are small?

- A: Yes. Since these islands are small compared to Hainan Island, Taiwan, or Japan's Kyushu Island, we judged them as small islands.

Q19. Line 335: change “to” to “at”.

- A: We changed it.

Q20. Line 339: I would change "are expected" to "can be expected." I think it is a reasonable expectation, but it is not a certainty.

- A: We agreed it and changed the sentence.

Q21. Line 349. since you have already said that this turtle is a sub-adult, you can delete "immature" here. It is not clear if it refers to the turtle or the foraging area.

- A: We deleted it.

Q22. Line 361: insert “on” between forage and seaweed.

- A: We inserted it.

Q23. Line 364: change “maintained” to “remained”

- A: We changed it.

Q25. Line 366: use “seagrasses” instead of “seagrass”. It sounds more natural.

- A: We changed it.

Q26. Line 367: change "below SST of 23°C in our study" to "with a SST below 23°C in our study"

- A: We changed it.

Q27. Line 369: change “supports” to “indicates”.

- A: We changed it.

Q28. Line 370: change "in mainly warm season" to "mainly in the warm season".

- A: We changed it.

Q29. Line 400-1: change “them” to “turtles”.

- A: We changed it.

Q30. Line400-2: I would delete the "north part"---just say the Northwest Pacific Ocean. The north part of the Northwest Pacific Ocean implies points north of Japan, perhaps as far north as Kamchatka.

- A: We deleted it.

Q31. Line 401: Change "could be underestimated considering the adaptation to the sea and migration distances to suitable habitats caused by the rehabilitation period in artificial facilities" to "could be inaccurate given the status of the studied animals as rescued".

- A: We replaced it.

Q32. Line 405: change “adapting” to “re-adapting”.

- A: We changed it.

Q33. Line 413: insert “surface” before sea temperature.

- A: We inserted it.
